# Computational repurposing of therapeutic small molecules from cancer to pulmonary hypertension

**DOI:** 10.1126/sciadv.abh3794

**Published:** 2021-10-20

**Authors:** Vinny Negi, Jimin Yang, Gil Speyer, Andres Pulgarin, Adam Handen, Jingsi Zhao, Yi Yin Tai, Ying Tang, Miranda K. Culley, Qiujun Yu, Patricia Forsythe, Anastasia Gorelova, Annie M. Watson, Yassmin Al Aaraj, Taijyu Satoh, Maryam Sharifi-Sanjani, Arun Rajaratnam, John Sembrat, Steeve Provencher, Xianglin Yin, Sara O. Vargas, Mauricio Rojas, Sébastien Bonnet, Stephanie Torrino, Bridget K. Wagner, Stuart L. Schreiber, Mingji Dai, Thomas Bertero, Imad Al Ghouleh, Seungchan Kim, Stephen Y. Chan

**Affiliations:** 1 Center for Pulmonary Vascular Biology and Medicine, Pittsburgh Heart, Lung, Blood, and Vascular Medicine Institute, Division of Cardiology, Department of Medicine, University of Pittsburgh School of Medicine and University of Pittsburgh Medical Center, Pittsburgh, PA, USA.; 2 Research Computing, Arizona State University, Tempe, AZ, USA.; 3 Department of Cardiovascular Medicine, Tohoku University of Graduate School of Medicine, 1-1 Seiryomachi, Aoba-ku, 980-8574 Sendai, Japan.; 4 Division of Pulmonary and Critical Care Medicine, Department of Medicine, University of Pittsburgh School of Medicine and University of Pittsburgh Medical Center, Pittsburgh, PA, USA.; 5 Pulmonary Hypertension and Vascular Biology Research Group, Faculty of Medicine, Laval University, Quebec, QC, Canada.; 6 Department of Chemistry, Center for Cancer Research, Institute for Drug Discovery, Purdue University, West Lafayette, IN, USA.; 7 Department of Pathology, Boston Children’s Hospital, MA, USA.; 8 Division of Pulmonary, Critical Care, and Sleep Medicine, Department of Medicine, Ohio State University College of Medicine, Columbus, OH, USA.; 9 Université Côte d’Azur, CNRS, IPMC, Sophia-Antipolis, France.; 10 Department of Chemistry and Chemical Biology, Harvard University; Chemical Biology and Therapeutics Science Program, Broad Institute of MIT and Harvard, Cambridge, MA, USA.; 11 Prairie View A&M Univ, Prairie View, TX, USA.

## Abstract

Cancer therapies are being considered for treating rare noncancerous diseases like pulmonary hypertension (PH), but effective computational screening is lacking. Via transcriptomic differential dependency analyses leveraging parallels between cancer and PH, we mapped a landscape of cancer drug functions dependent upon rewiring of PH gene clusters. Bromodomain and extra-terminal motif (BET) protein inhibitors were predicted to rely upon several gene clusters inclusive of galectin-8 (LGALS8). Correspondingly, LGALS8 was found to mediate the BET inhibitor–dependent control of endothelial apoptosis, an essential role for PH in vivo. Separately, a piperlongumine analog’s actions were predicted to depend upon the iron-sulfur biogenesis gene ISCU. Correspondingly, the analog was found to inhibit ISCU glutathionylation, rescuing oxidative metabolism, decreasing endothelial apoptosis, and improving PH. Thus, we identified crucial drug-gene axes central to endothelial dysfunction and therapeutic priorities for PH. These results establish a wide-ranging, network dependency platform to redefine cancer drugs for use in noncancerous conditions.

## Teaser

A network dependency platform was generated to define a landscape of cancer drug mechanisms in pulmonary hypertension.

## INTRODUCTION

The repurposing of small molecules for disease therapy has gained traction, given the potential to reduce cost and time necessary for de novo drug development. Specifically, computational drug repurposing is emerging as a viable method, leveraging available large-scale clinical and molecular profiling and combining those with in silico methodologies of machine learning, network modeling, and clinical text mining to define novel drug activities (*1*). However, the vast majority of such strategies have depended upon identification of differentially expressed genes (DEGs) (*2*), which can define some but not all key intergenic relationships. Molecular network mapping using connections among genes with a tendency to be regulated together (i.e., gene regulatory dependencies) addresses some of the DEG-based analysis limitations. However, because of the large amount of data required for calculating differential dependencies across gene networks, such analytics are often not feasible across the limited -omics datasets of rare or emerging diseases.

Pulmonary hypertension (PH) represents such an enigmatic vascular disease that consists of five World Symposium of Pulmonary Hypertension (WSPH) groups (*3*). In particular, WSPH group 1 (pulmonary arterial hypertension, PAH) and group 3 (due to hypoxic lung disease) PH subtypes are driven by shared triggers of hypoxia and inflammation, and mortality is high. Current medications primarily vasodilate, are mostly used to treat group 1 PAH, and are not curative. Thus, an unmet need exists for new drug discovery. In particular, endothelial pathobiology is a characteristic and pathogenic feature of PH contributing to the inflammation and aberrant vascular remodeling observed in this disease (*4*). However, because of complex spatio-temporal manifestations that balance critical processes such as apoptosis and proliferation (*5*, *6*) during disease progression, therapeutic targeting of endothelial dysfunction in PH has been challenging. The advancing appreciation of broad molecular parallels between PH and cancer in general (*7*), as well as the direct link between developing PH in the setting of lung cancer specifically (*8*), have increased enthusiasm for repurposing existing small-molecule inhibitors from cancer to PH (*9*). This may be particularly relevant for precise therapeutic targeting of dysregulated endothelial survival—a process also crucial for hypoxic and inflammatory-driven tumorigenesis (*10*). To date, however, the broad molecular profiling existing in cancer datasets has yet to be leveraged for such PH drug discovery. Hence, mapping gene regulatory dependency networks relevant to PH and investigating “rewiring” of these networks in connection to cancer drug activity present a unique opportunity. Here, we hypothesized that deep analysis of the relationship between drug response and molecular rewiring in cancer cells of pathways implicated both in PH and cancer will offer insight into how vascular cells in PH will respond to specific drugs and, in turn, support repurposing of these drugs for PH.

To investigate this notion, we sought to design a computational strategy identifying differential dependency networks (DDNs) of genes in cancer cells associated with drug response and overlapping with a rare disease such as PH. Namely, we applied the capabilities of EDDY (Evaluation of Differential DependencY) (*11*), a prior-knowledge–assisted algorithm that defines DDNs based on the rewiring of dependency interactions among genes in a network under different conditions, for example, cancer cell’s response to drug. EDDY has been used in the study of human diseases (*11*) and transcriptomic analyses from human PH lung tissue (*12*) to identify DDNs in disease. EDDY was applied to a dataset derived from the Cancer Cell Line Encyclopedia [CCLE; encompassing a catalog of RNA sequencing data from 810 cancer cell lines (*13*)] and the Cancer Therapeutics Response Portal [CTRP; surveying the response of those cell lines to 368 small molecules (*14*, *15*)]. In doing so, for each cancer drug surveyed, this EDDY-CTRP identified DDNs that define drug response by virtue of their specific rewiring in sensitive versus resistant cells (*16*).

Leveraging those principles, we developed a computational platform (EDDY-CTRP-PH) to predict the landscape of cancer drug functions that rely upon rewired DDNs of genes common to cancer and PH as well as have shared links to hypoxia and inflammation—thus exerting robust activity in controlling multiple PH subtypes. We identified and experimentally tested two highly ranked candidate drugs and their predicted gene network effectors. First, bromodomain and extra-terminal motif (BET) protein inhibitors, which target the epigenetic modifiers bromodomain containing 2/4 (Brd2/4) and are already being tested in PH (*17*–*19*), were predicted to selectively affect a PH gene cluster encompassing galectin-8 (LGALS8). LGALS8, a member of the galectin family that regulates inflammation (*20*) and apoptosis (*21*), has not been previously implicated in PH nor connected to BET inhibitors. Second, an analog of the alkaloid piperlongumine, BRD-K34222889 or BRD2889 (*22*), was predicted to selectively target a PH cluster dependent on the iron-sulfur biogenesis gene ISCU. While deficiency of endothelial ISCU is known to drive PH (*23*), and piperlongumine and its analogs are reported to inhibit glutathione *S*-transferase pi 1 (GSTP1) (*24*), any functional connections among BRD2889 and GSTP1 to ISCU and to PH have yet to be reported. Thus, by coupling in vitro and in vivo experimentation with our in silico findings, we sought to define a computational-to-empirical pipeline for identifying and ranking the most robust actions of specific cancer therapeutics, revealing their disease-relevant downstream targets in an example of a rare noncancerous disease such as PH.

## RESULTS

### EDDY-CTRP-PH: In silico mapping of small molecules that depend upon rewired PH-specific DDNs for activity

**Fig. 1.**
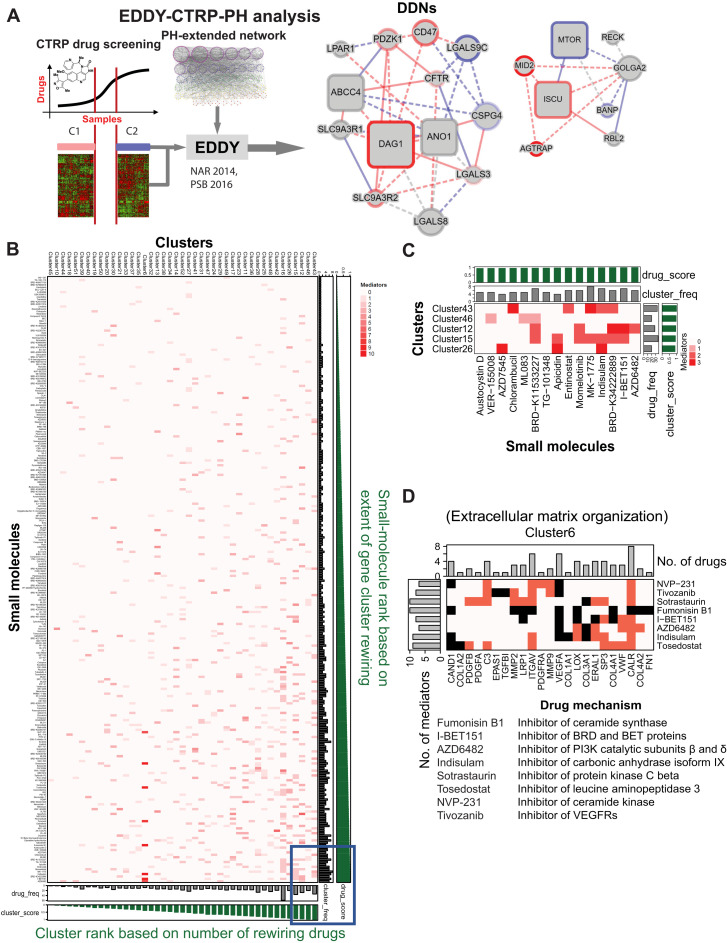
EDDY-CTRP-PH provides in silico predictions of small molecules that depend upon rewired PH-specific DDNs for activity. (**A**) In silico workflow: EDDY-CTRP-PH identifies relationships between nodes (genes) in DDNs where each characteristic line indicates the identified relationship: drug-sensitive (red), drug-resistant (blue), and both (gray) and known interactions (solid) and previously unknown statistical dependencies (dashed). Size and shape of the nodes indicated the role of a given gene in the structural network integrity of the DDN—large nodes reflected the degree of betweenness centrality, and square nodes represented essentiality or specificity mediators of the DDN, as described in the Supplementary Materials. (**B**) EDDY-CTRP-PH data landscape: Clusters and small molecules were sorted according to their score and represented as a heatmap, where increasing red intensity denotes the number of mediators involved in a particular cluster-drug interaction. Green bar graphs along *x* and *y* axes: score of each small molecule and cluster, respectively; Gray bar graphs: frequency of significantly rewired clusters for a given small molecule and frequency of small molecules linked to rewiring of a given cluster. (**C**) High-activity hotspot linking small molecules with PH clusters: Visualization representing the bottom right portion (blue box) of heatmap in (B) containing top 5 clusters and 15 small molecules. (**D**) Predictions of small molecules affecting cluster 6: Visualization of cluster 6, enriched in extracellular matrix (ECM) genes, indicates convergence of eight small molecules with known and previously unknown (e.g., for fumonosin B1 and indisulam) associations with ECM biology. Black cells: predicted gene (column)–drug (row) interaction; red cells: literature support of interaction. Bar plots on *x* and *y* axes: frequency of drugs and mediators, respectively. Listed below the figure are the drug mechanisms. Information on PH clusters, drugs, and cluster scores is provided in tables S1 and S2. VEGFRs, vascular endothelial growth factor receptors.

To identify PH-specific DDNs that mediate crucial cellular responses to specific small molecules, a catalog of gene networks integral to PH pathogenesis was necessary for initial input. Building upon our prior methodology (*25*), 55 PH-relevant gene clusters were identified (table S1) and analyzed by EDDY in the context of the CCLE and CTRP datasets (EDDY-CTRP-PH workflow; Fig. 1A) to discover PH gene clusters strongly associated with a cancer cell’s response to drugs and mediators for each PH gene cluster. Namely, for each cancer drug tested, cell lines were categorized into two groups: drug-sensitive and drug-resistant, as described (*16*). For each drug, transcriptomic profiles were analyzed by EDDY to define PH-relevant gene clusters that displayed significant rewiring of DDNs between sensitive versus resistant cell lines. Then, for each DDN, genes important to the network connectivity, denoted as mediators, were identified by network analysis as those that have most control over the network. Two types of mediators were defined. “Condition-specific” mediators were genes with a significant proportion of condition-specific edges (i.e., drug sensitivity versus resistance), emphasizing their unique importance in controlling specific drug responses. “Essentiality” mediators were those genes that depended upon the betweenness centrality metric—a measure of how often network information will pass through that node. Essentiality mediators were those genes meeting a betweenness-centrality difference cutoff between the condition-specific (i.e., drug sensitivity versus resistance) networks—thus, “essential” to DDN rewiring. In total, such DDN rewiring and mediator identification predicted previously unknown roles of these clusters and dependencies in mediating the actions of each drug and, consequently, the direct relevance to molecular PH pathogenesis. These results are available at the following website (https://chan.vmi.pitt.edu/eddy-ctrp-ph/).

To assess the landscape of functional connections cataloged by EDDY-CTRP-PH, small molecules and clusters were sorted according to their rewiring scores as defined in Materials and Methods (Fig. 1B and tables S1 and S2). The top 5 clusters and top 15 small molecules represented candidates for further in-depth study (Fig. 1C). These predictive outputs offered a number of wide-reaching insights. First, 60% of small molecules were predicted to be selective for rewired gene dependencies across at least two or more PH clusters, indicating substantial overlap of activity of the cancer drug landscape with PH pathogenic processes (full website listing under EDDY-CTRP-PH: Individual Drugs). Second, among the gene mediators identified by EDDY as essential for orchestrating PH DDN rewiring, 53.6% (165 of 308 mediators identified) have not previously been linked to PH pathogenesis. Third, among the gene dependencies mapped by EDDY within the PH gene clusters, 72.3% represented functional connections not previously described (1,230 of 1,700 wiring connections).

The EDDY-CTRP-PH platform also offered granular molecular information via either defining previously unknown PH pathways targeted by known PH drugs or identifying connections linking novel drugs to known PH pathways. As an example of the former, in addition to being selectively sensitive to a DDN enriched for oxidative metabolism genes via well-established links (cluster 23) (*26*), the pyruvate dehydrogenase kinase (PDK) inhibitor AZD7545 was predicted to be associated with the DDNs of immune response cluster 37 (tumor necrosis factor signaling) and cluster 26 (CD4 lymphocyte signaling). Similarly, while canonically PDE5 inhibitors are used clinically to treat PH in the context of nitric oxide signaling and regulation of vasomotor tone, EDDY-CTRP-PH predicted sildenafil as dependent upon rewiring of PH gene cluster 28, a cluster enriched particularly with genes involved in apoptosis. Evidence of PDE5 activity in apoptosis and cell survival has more recently been reported (*27*), offering validation of such predictions. Moreover, DDNs with previously unknown functions were uncovered for other drugs with already existing PH connections, including the histone deacetylase (HDAC) inhibitor apicidin and the carbonic anhydrase inhibitor indisulam, among others (tables S1 and S2, full website listing under EDDY-CTRP-PH: Individual Drugs). Alternatively, EDDY offered an ability to define complementary drugs that converge upon a single PH pathway (full website listing under EDDY-CTRP-PH: Cluster View). For example, eight small molecules were found to converge upon cluster 6, a network enriched for extracellular matrix (ECM) organization genes (Fig. 1D). While some of these drugs already carried known associations with ECM biology, EDDY revealed this functional connection for other drugs, such as fumonosin B1 and indisulam, that had not previously been linked to matrix organization.

### EDDY-CTRP-PH identifies a functional connection between BET inhibitors and cluster 15

**Fig. 2.**
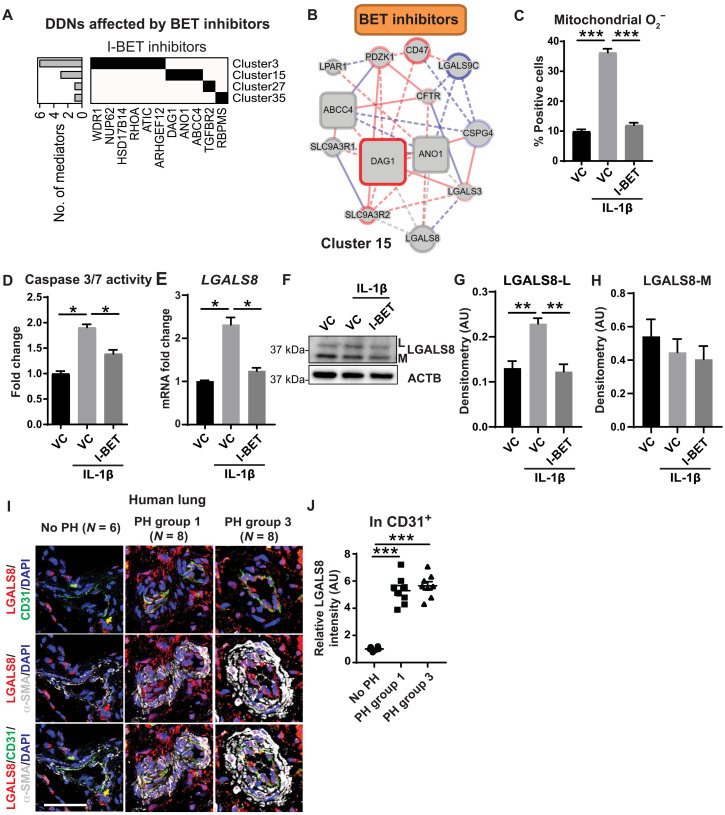
I-BET protects against apoptosis and alters C15 gene expression in cultured PAECs. (**A**) Schematic representation of clusters and relevant mediator genes demonstrated rewiring across four PH DDNs (clusters 3, 15, 27, and 35) by all three BET inhibitor drugs represented in CTRP (I-BET151, I-BET762, and JQ-1). Black cells: the cluster to which each mediator belongs. Bar graph on *y* axis: number of BET inhibitor–associated mediators for each cluster. (**B**) DDN of cluster 15 representing rewiring associated with the collective actions of all three BET inhibitors; red, drug-sensitive interactions; blue, drug resistant; gray, both. Solid lines, known interactions; dotted lines, new statistically determined dependencies; square boxes, critical mediators. (**C** to **E**) In PAECs ± IL-1β exposure, I-BET762 (I-BET), when compared with vehicle control (VC), reversed the IL-1β–induced increases of (C) mitochondrial superoxide (O_2_^−^) levels as determined by flow cytometery of MitoSOX Red staining (*n* = 5 per group), (D) apoptosis as assessed by caspase-3/7 activity (*n* = 6 per group), and (E) expression of cluster 15 (C15) gene galectin-8 (*LGALS8*) as determined by quantitative reverse transcription polymerase chain reaction (RT-qPCR) (*n* = 3 per group). (**F** to **H**) By representative immunoblot (F) and densitometry of LGALS8-L (G) and LGALS8-M (H) in PAECs (*n* = 3 per group), I-BET reversed the IL-1β–induced increase of the L isoform, but not the M isoform, of LGALS8, as compared with VC. ACTNB, b-actin. (**I** and **J**) Using immunofluorescence staining (I) and respective quantification, expression of LGALS8 was increased in CD31^+^ pulmonary arteriolar endothelium (J) of human patients with WSPH group 1 (*n* = 8) and group 3 (*n* = 8) PH as compared to non-PH controls (*n* = 6). Scale bars, 50 μm. Data from (C) to (H) are represented as fold change with respect to Un and plotted as means ± SEM. Statistical significance is indicated using one-way analysis of variance (ANOVA) with Bonferroni’s multiple comparisons testing (*P < 0.05, **P < 0.01, and ***P < 0.001). AU, arbitrary units. DAPI, 4′,6-diamidino-2-phenylindole.

We sought to experimentally validate key predictions by EDDY-CTRP-PH, linking as-of-yet undiscovered PH pathways to drugs currently under therapeutic development. An example included the epigenetic drug class of BET inhibitors being tested for PH treatment (*19*). Notably, BET inhibitors have mainly been studied in the context of smooth muscle cells in PH (*17*, *18*), with only a partial identification of BET activity in controlling endothelial processes in this disease (*19*). Three BET inhibitors (JQ-1, I-BET151, and I-BET762) were included in EDDY-CTRP-PH analyses, with I-BET151 emerging as one of the top-ranked drugs (Fig. 1C). In recalculating DDNs relevant across all drugs modulating the same target (i.e., DDNs shared across the same drug class), EDDY found four rewired PH DDNs associated with all three BET inhibitors (Fig. 2A; full website listing under EDDY-CTRP-PH: Drug Class). The predominant actions of some of these PH DDNs, such as Rho guanosine triphosphatase (cluster 3) (*28*) and transforming growth factor–β signaling (cluster 27) (*29*), are known to be controlled by epigenetic BET functions. However, EDDY also identified previously unknown functional connections of BET inhibition to previously unannotated DDNs, such as clusters 15 and 35. We further deemed cluster 15 (C15) a “hotspot” gene cluster, since nearly half of the top small molecules (7 of 15, including I-BET151) were predicted to depend upon C15 rewiring for activity (Fig. 1C). Validation of predictions relating C15 to BET inhibitors therefore offered the greatest opportunity for identifying novel insights. Given the alteration in expression of a majority of C15 genes in response to PH triggers and I-BET inhibitors in endothelial cells (fig. S1, A and D), we focused on this cell type.

### I-BET protects against apoptosis and alters C15 gene expression in PAECs

To predict functional convergence of BET inhibition on specific C15 genes, we reconstructed the C15 DDN for the collective actions of all BET inhibitors (Fig. 2B; full website listing under EDDY-CTRP-PH: Drug Class). Using this DDN as a guide, we sought to define experimentally the predicted roles of these BET inhibitors. Given the known mechanistic connections of BET inhibitors to interleukin-1β (IL-1β) (*30*) for modulating inflammatory phenotypes, an IL-1β–induced model of endothelial dysfunction was used to recapitulate PH features in vitro (*31*). We chose to study I-BET762 (labeled as I-BET hereafter), because I-BET762 and I-BET151 exhibited similar control of C15 genes (fig. S1A), and I-BET762 exhibited more favorable characteristics in clinical trials compared with either I-BET151 or JQ-1 (*32*). To determine the global transcriptomic effects of I-BET in pulmonary artery endothelial cells (PAECs), microarray profiling was performed after chronic exposure to IL-1β with or without I-BET (fig. S1B and table S3). Gene set enrichment analysis of 524 DEGs revealed specific enrichment of pathways relevant to cell death, metabolism, and endothelial function, altered by IL-1β but reversed by I-BET (fig. S1B and table S4). Consistent with these transcriptomic results and with the known importance of endothelial redox alterations and apoptosis in PH (*33*), we found that I-BET reduced the IL-1β–dependent increase of mitochondrial superoxide (O_2_^−^) and apoptosis in PAECs (Fig. 2, C and D, and fig. S1C).

To determine the relevance of C15 genes in such endothelial function, expression of C15 genes was measured under the same conditions. Seven of 11 C15 genes were expressed in human PAECs, and six of those—*LGALS3*, *LGALS8*, *ABCC4*, *CD47*, *SLC9A3R1*, and *DAG1*—were reversed by I-BET (Fig. 2E and fig. S1, A and D). Of those six C15 genes, only four (*LGALS3*, *LGALS8*, *DAG1*, and *SLC9A3R1*) displayed near complete reversal by I-BET, with *LGALS8* transcript (galectin-8) showing the largest fold change alteration with IL-1β. Thus, these findings suggested an as-of-yet undescribed importance for *LGALS8* in this regulatory axis that we pursued further experimentally. In pulmonary artery smooth muscle cells (PASMCs) in the presence of IL-1β, alterations and reversals by IL-1β and I-BET were not observed across the same C15 genes including *LGALS8* (fig. S1E), indicating the cell type specificity of these I-BET-C15 connections and further guiding a focus on PAECs. In addition, on the basis of lung staining data from the Human Protein Atlas (www.proteinatlas.org), most vascular galectin-8 is localized in endothelial cells, supporting the notion of a connection between I-BET and this C15 gene in this cell type.

Of the two major isoforms of LGALS8, LGALS8-M and LGALS8-L (*20*), we found that *LGALS8-L* transcript in cultured PAECs was increased by IL-1β and reversed by I-BET, whereas LGALS8-M was reduced by IL-1β but not altered by I-BET (fig. S1D). LGALS8-L protein followed its mRNA expression, but LGALS8-M showed no significant difference (Fig. 2, F to H). Next, phenocopying I-BET, knockdown of either the canonical targets of I-BET, *BRD2*, or *BRD4* [small interfering RNA (siRNA) efficacy confirmed in fig. S1F], blunted IL-1β–specific increases of *LGALS8-L* at the transcript and protein levels (fig. S1, F to H). Thus, I-BET depends upon Brd2/4 to regulate *LGALS8* and LGALS8-L in endothelial cells.

To demonstrate the translational relevance of these findings, LGALS8 was stained in pulmonary arterioles (<100 μm in diameter) of two WSPH subtypes (table S5): those with severe group 1 PAH and those group 3 PH due to hypoxic lung disease. LGALS8 was up-regulated in the pulmonary vasculature, consistent with its known intracellular and extracellular forms and with notable increased expression CD31^+^ endothelial cells (Fig. 2, I and J) but no change observed in circulating venous plasma levels (fig. S2A). Moreover, consistent with our findings in cultured PAECs, LGALS8 was concurrently up-regulated in three separate rodent models of PH including chronically hypoxic mice (fig. S2, B to K), along with IL-1β in both humans and rodents with PH (fig. S2, L to S), thus emphasizing the inherent inflammatory component of PH and direct relevance to LGALS8.

**Fig. 3.**
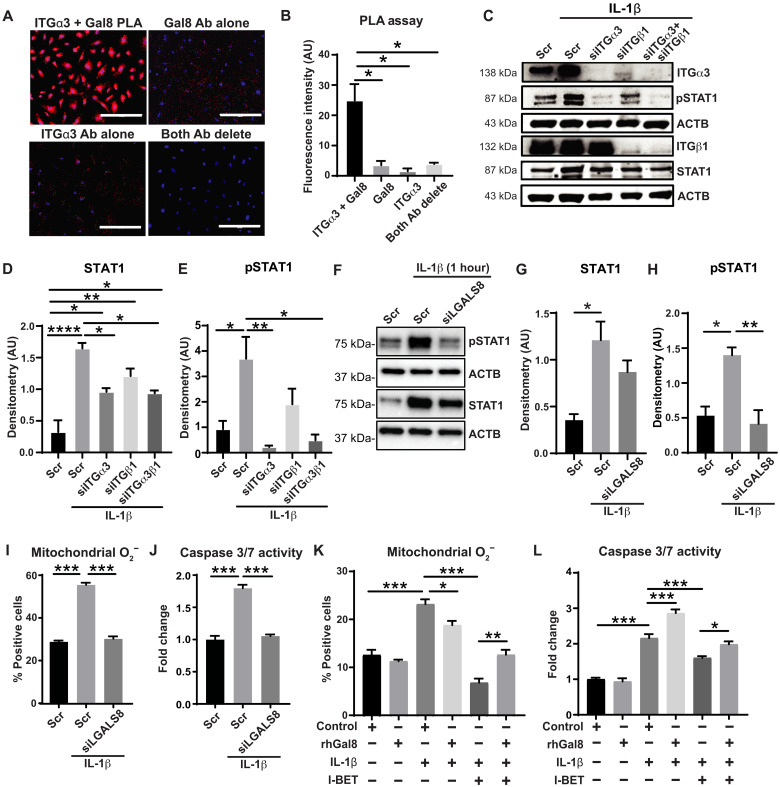
LGALS8 is a major effector of C15 controlling endothelial cell apoptosis via STAT1 signaling. (**A** and **B**) Interaction of LGALS8 and the α3 subunit of integrin α3β1 was demonstrated by proximity ligation assay (PLA) in PAECs. Positive interaction was depicted by Texas Red signal; blue, DAPI. Controls include deletion of either antibody or both (*n* = 3 per group). Scale bars, 200 μm. Ab, antibody. (**C** to **E**) In PAECs, representative immunoblot (C) and densitometry demonstrated increased STAT1 (D) and pSTAT1 (E) levels with IL-1β exposure (1 hour); these levels were attenuated by knockdown of integrin α3 (siITGα3), integrin β1 (siITGβ1), or both (*n* = 3 per group). (**F** to **H**) By representative immunoblots (F) and densitometry of total STAT1 (G) and phosphorylated STAT1 (pSTAT1, H) in PAECs, knockdown of LGALS8 (siLGALS8) attenuated the IL-1β (1 hour)–induced increase of pSTAT1 (*n* = 3 per group). (**I** and **J**) Similarly, siLGALS8 reduced the IL-1β (48 hours)–dependent increases of mitochondrial O_2_^−^ as assessed by MitoSOX staining and flow cytometry (I) and apoptosis as assessed by caspase-3/7 activity (J) (*n* = 6 per group). (**K** and **L**) In IL-1β–exposed (48 hours) PAECs treated with I-BET and recombinant galectin-8 (rhGal8; 24 hours), rhGal8 reversed the I-BET–induced attenuation of mitochondrial O_2_^−^ (K) and caspase 3/7 activity (L) (*n* = 3 to 6 per group). Data plotted as means ± SEM. Statistical significance is indicated using one-way ANOVA with Bonferroni’s multiple comparisons testing (*P < 0.05, **P < 0.01, ***P < 0.001, and ****P < 0.0001).

Consistent with the known binding of integrin receptor α3β1 to LGALS8 in other contexts (*21*), we demonstrated integrin α3 (ITGα3) binding to LGALS8 in PAECs using a proximity ligation assay (Fig. 3, A and B). Furthermore, given the actions of signal transducer and activator of transcription 1 (STAT1) in integrin signaling and its importance in generating mitochondrial reactive oxygen species and apoptosis (*34*), we examined its activation status downstream of the LGALS8–integrin α3β1 interaction. Knockdown of integrin α3β1 (siITGα3/siITGβ1; fig. S3, A and B) or integrin α3 alone reduced IL-1β–induced STAT1 phosphorylation (Fig. 3, C to E), phenocopying *LGALS8* (siLGALS8; Fig. 3, F to H, and fig. S3C). Next, revealing the role of LGALS8 in endothelial pathobiology, siLGALS8 decreased IL-1β–induced mitochondrial superoxide and apoptosis (Fig. 3, I and J, and fig. S3D) and phenocopied the effects of I-BET. To determine whether I-BET depends critically upon LGALS8 to alter mitochondrial superoxide and apoptosis, recombinant human LGALS8 (rhGal8) was used to supplement LGALS8 function. While rhGal8 alone had no effect, its presence reduced the ability of I-BET to decrease IL-1β–specific mitochondrial superoxide (Fig. 3K and fig. S3E) and apoptosis (Fig. 3L). Collectively, these data demonstrate the critical role of LGALS8, via integrin α3 and STAT1 activation, in mediating I-BET action on IL-1β–driven PAEC dysfunction.

### I-BET reduces endothelial LGALS8 and improves existing PAH in rats

**Fig. 4.**
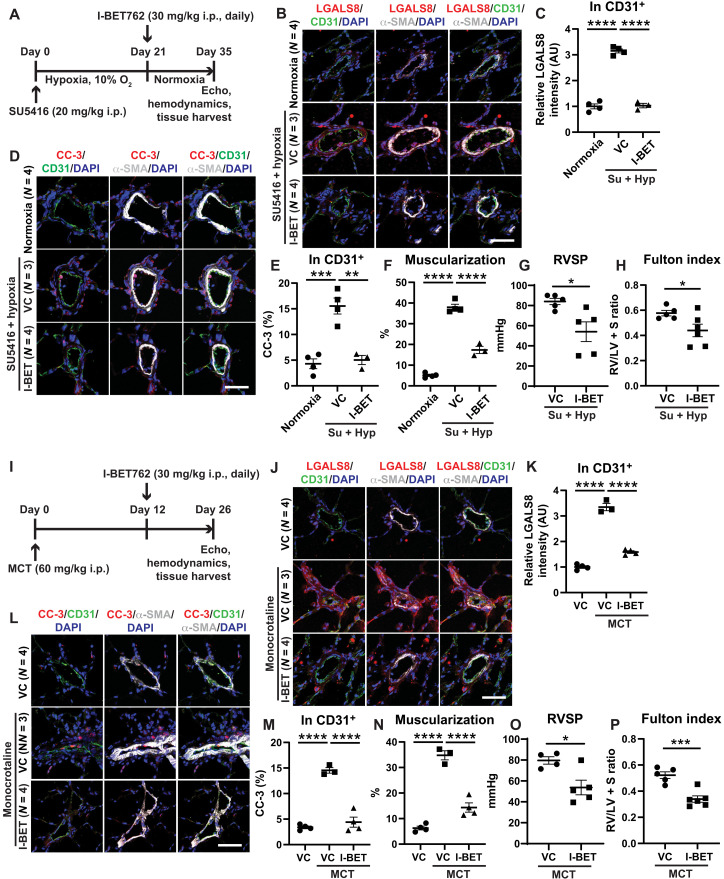
I-BET762 reduces LGALS8, endothelial apoptosis, and improves existing PAH in multiple PAH rat models. (**A**) Sprague-Dawley rats were administered SU5416 intraperitoneally (i.p.) (20 mg/kg) followed by hypoxia for 21 days to promote PAH. Rats were then treated with I-BET762 versus vehicle control by daily intraperitoneal injection (30 mg/kg) at days 21 to 35 in normoxia (*n* = 3 to 6 per group). (**B** to **E**) By immunofluorescence staining and quantification of LGALS8 (B to E) and cleaved caspase-3 (CC-3) expression (D and E) in pulmonary arterioles, I-BET decreased LGALS8 and apoptotic CC-3, notably in CD31^+^ endothelium. (**F** to **H**) I-BET reduced arteriolar muscularization (F), right ventricular systolic pressure (RVSP) (G), and Fulton index [right ventricle (RV)/left ventricle + septum (LV + S) mass ratio; H]. (**I**) Sprague-Dawley rats were administered MCT intraperitoneally (60 mg/kg) to promote PAH within 3 weeks. Rats were then treated with I-BET762 versus vehicle control by daily intraperitoneal injection (30 mg/kg) at days 12 to 26 after MCT injection (*n* = 3 to 6 per group). (**J** to **M**) By immunofluorescence staining and quantification of LGALS8 (J and K) and cleaved caspase-3 (CC-3) expression (L and M) in pulmonary arterioles, I-BET decreased LGALS8 and apoptotic CC-3, notably in CD31^+^ endothelium. (**N** to **P**) I-BET reduced arteriolar muscularization (N), RVSP (O), and Fulton index (RV/LV + S; P). Data are plotted as means ± SEM. Scale bars, 50 μm. Statistical significance is indicated using one-way ANOVA with Bonferroni’s multiple comparisons testing (*P < 0.05, **P < 0.01, ***P < 0.001, and ****P < 0.0001).

To investigate whether I-BET controls LGALS8 and PAH in vivo, I-BET was intraperitoneally administered daily in two separate models of group 1 PAH in rats—monocrotaline (MCT) exposure followed by 26 days in normoxia and SU5416-hypoxia exposure (3 weeks, days 0 to 21) followed by 2 weeks of normoxia (days 21 to 35). In both exposures, a disease-reversal protocol was used, whereby I-BET was administered only after disease manifested (at days 12 to 26 after MCT and at days 21 to 35 after 3 weeks of SU5416-hypoxia) (Fig. 4, A and I). Echocardiographic assessment after I-BET762 dosing in SU5415-hypoxic rats demonstrated no alteration of heart rate, left ventricular function, or aortic pressure after drug dosing (fig. S4). Consistent with our in vitro findings in cultured PAECs, I-BET decreased pulmonary vascular LGALS8, including in endothelial cells (Fig. 4, B, C, J, and K). Consequently, I-BET reduced downstream apoptosis, as reflected by reduced cleaved caspase-3 (Fig. 4, D, E, L, and M). As with prior studies (*19*) of other BET inhibitors in PAH rats, in both rat models, we observed a reduction of indices of disease, including reduced pulmonary vascular muscularization [via alpha-smooth muscle actin (α-SMA) stain], right ventricular systolic pressure (RVSP), and Fulton index (Fig. 4, F to H and N to P).

### I-BET and genetic deficiency of LGALS8 protect against hypoxic PH in mice

**Fig. 5.**
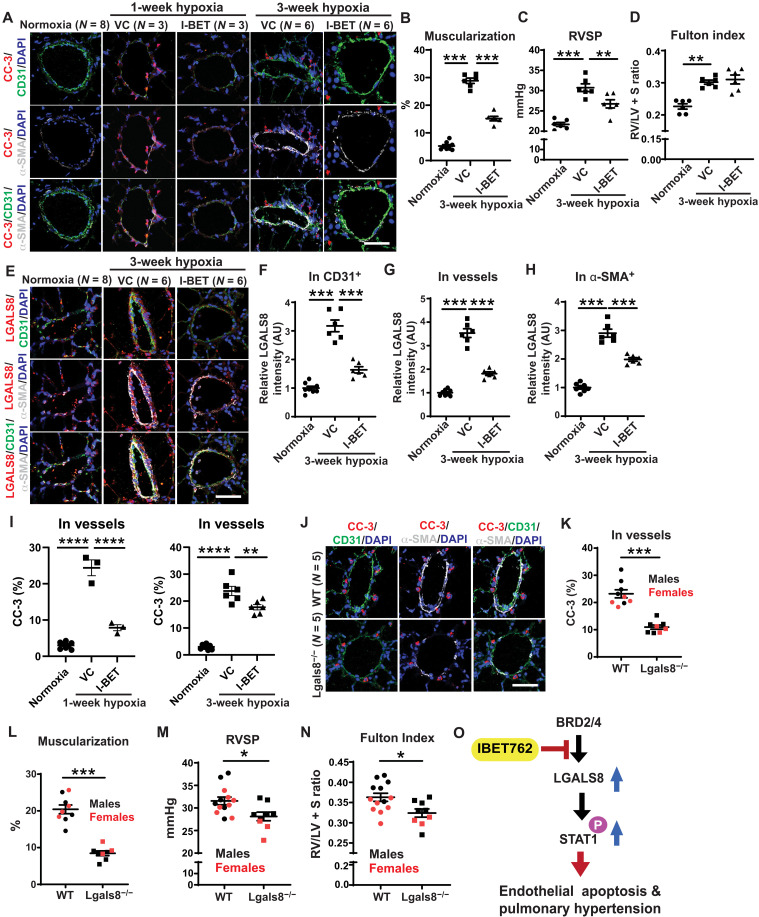
I-BET and genetic deficiency of LGALS8 independently protect against hypoxia-induced PH in mice. (**A** to **I**) Wild-type (WT) mice were exposed to 1 or 3 weeks of hypoxia and treated with daily I-BET versus VC. VC-treated normoxic mice were used as comparators (*n* = 3 to 6 per group). With the exception of Fulton index, I-BET reversed the 3-week hypoxia-dependent increases of these indices: muscularization as indicated by α-SMA^+^ staining (A and B); RVSP (C); Fulton index (RV/[LV + S] mass ratio) (D); LGALS8 expression (E) in CD31^+^ endothelial cells (F), whole arterioles (G), or α-SMA^+^ smooth muscle cells (H); and cleaved caspase-3 (CC-3, I). Consistent with the fact that endothelial apoptosis in PH is more readily observed early in disease (*66*), the reduction of endothelial CC-3 by I-BET was more prominent at the earlier 1-week hypoxia time point. (**J** to **N**) In parallel, as compared with hypoxic WT mice, hypoxic *Lgals8^−/−^* mice displayed reductions in vascular cleaved caspase-3 (J and K), muscularization (L), RVSP (M), and Fulton index (N) (*n* = 8 to 9 *Lgals8^−/−^* and 8 to 13 WT; black, male; red, female). (**O**) Cartoon representing effect of I-BET on Lgals8 expression, controlling downstream STAT signaling pathway, endothelial apoptosis, and PH. Data are plotted as means ± SEM. Statistical significance is indicated using one-way ANOVA with Bonferroni’s multiple comparisons testing for (A) to (I) and Student’s *t* test for (J) to (N) (*P < 0.05, **P < 0.01, ***P < 0.001, and ****P < 0.0001). Scale bars, 50 μm.

Stemming from the known link between hypoxia and inflammatory activation (*35*), we reasoned that IBET-762 and LGALS8 may also be relevant to inflammatory pathways activated in hypoxia and thus in group 3 PH due to hypoxic lung disease. Hence, in cultured PAECs, we found that IBET-762 and LGALS8 control inflammatory and apoptotic endothelial pathways driven by hypoxia (fig. S5). Correspondingly, we studied the effects of daily and orally administered I-BET for 2 weeks in a group 3 PH model—chronically hypoxic mice. As in PAH rats, we observed an amelioration of PH indices, including reduced pulmonary vascular muscularization (via α-SMA stain; Fig. 5, A and B) and RVSP (Fig. 5C), without significant effect on Fulton index (Fig. 5D) or heart rate (fig. S6A). Consistent with our in vitro findings in cultured PAECs and known intracellular and extracellular forms of this protein, I-BET robustly decreased pulmonary vascular LGALS8, including in endothelial cells (Fig. 5, E to H). Notably, similar dosing of I-BET in normoxic mice did not alter LGALS8 [1 ± 0.02 fold change with vehicle control versus 1.08 ± 0.03 fold change with I-BET, means ± SEM, *N* = 3 to 4 per group, *P* = not significant (NS)], consistent with the known principle that such inhibitors offer the most robust endothelial effects under inflammatory conditions (*36*). Consequently, I-BET reduced downstream apoptosis, as reflected by reduced cleaved caspase-3 most notably seen at early stages of disease when endothelial apoptosis is highest (Fig. 5, A and I) (*4*). Next, to define the role of LGALS8 in PH, male and female *Lgals8*^−/−^ versus wild-type (WT) mice (fig. S6, B to D) were exposed to chronic hypoxia (3 weeks). Echocardiography showed no significant difference in left ventricular functional indices or heart rate (fig. S6, E to H) in *Lgals8*^−/−^ versus WT mice under hypoxia. Furthermore, *Lgals8*^−/−^ mice did not display altered IL-1β expression compared with WT mice (fig. S6, I and J), indicating consistent upstream inflammatory stimulus in both groups. However, apoptosis, as quantified by cleaved caspase-3 immunoblot in whole lung lysate and by pulmonary arteriolar immunofluorescent stain, and pulmonary arteriolar muscularization were significantly reduced in *Lgals8*^−/−^ mice (Fig. 5, J to L, and fig. S6, K and L). *Lgals8*^−/−^ mice were protected from hemodynamic manifestations of PH, evidenced by lower RVSP and Fulton index versus WT mice (Fig. 5, M and N). There was no difference in RVSP between *Lgals8*^−/−^ versus WT mice under normoxia (18.51 ± 0.45 mmHg WT mice versus 19.24 ± 1.68 mmHg *Lgals8*^−/−^ mice, means ± SEM, *N* = 3 to 4 per group, *P* = NS). Together, as guided by EDDY-CTRP-PH predictions, in vitro and in vivo experimentation defined the regulation of endothelial Lgals8 and its downstream control of cellular apoptosis as a crucial mediator of I-BET’s therapeutic effects of PH (Fig. 5O).

### EDDY-CTRP-PH identifies a functional connection between BRD2889, its target GSTP1, and the cluster 43 gene ISCU

**Fig. 6.**
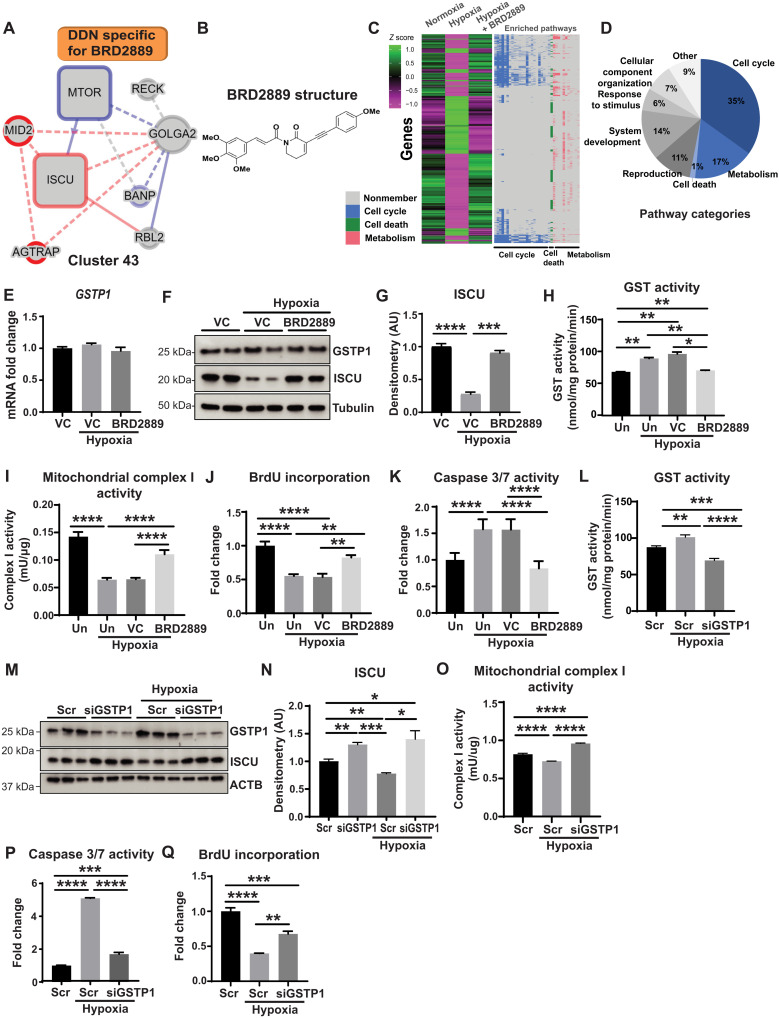
EDDY-CTRP-PH identifies a connection among BRD2889, its target GSTP1, and cluster 43 gene ISCU. (**A**) DDN for cluster 43 specific for BRD2889 predicted ISCU as a BRD2889-sensitive mediator. DDN annotations by colors, edges, and boxes are defined in Fig. 1A. (**B**) Structure of BRD2889. (**C**) PAEC expression array (*n* = 3 per group) identified genes significantly altered by hypoxia but reversed by BRD2889 (left heatmap). Heatmap (right) depicts gene membership in Gene Ontology (GO) processes relevant to ISCU-related activity. (**D**) Percentages of enriched GO terms from (C) with ISCU-related activity in blue. (**E** to **H**) As assessed by GSTP1 levels (E and F), GST activity (H) and ISCU immunoblot (F and G) in hypoxic PAECs (*n* = 3 per group), BRD2889 reversed hypoxic alterations of ISCU and GST activity. Untreated, Un. (**I** to **K**) In PAECs treated as in (H) (*n* = 4 per group), BRD2889 reversed hypoxic alterations of mitochondrial complex I activity (I), proliferation [via 5-bromo-2′-deoxyuridine (BrdU) incorporation] (J), and apoptotic caspase 3/7 activity (K). (**L** to **Q**) Compared with control (Scr) in hypoxic PAECs, GSTP1 knockdown (siGSTP1) phenocopied BRD2889 and reversed hypoxic changes in GST activity (L), GSTP1/ISCU protein (M and N), complex I activity (O), caspase 3/7 activity (P), and BrdU incorporation (Q) (*n* = 3 per group). Data are plotted as means ± SEM. Statistical significance is indicated using one-way ANOVA with Bonferroni’s multiple comparisons testing (*P < 0.05, **P < 0.01, ***P < 0.001, and ****P < 0.0001).

In addition to predictions of previously unknown pathways that mediate actions of drugs already under study for PH, EDDY-CTRP-PH also offered central insights into small molecules never before investigated in this disease and into their activities that have never before been connected to known PH pathways. To identify the most robust candidate drug-pathway axes, we focused on cluster 43 (C43), which had the highest level of rewiring across all small molecules tested (Fig. 1C). A novel analog of the anti-inflammatory and senolytic drug piperlongumine, BRD2889, known to inhibit the *S*-glutathionylation enzyme GSTP1, was the drug with the highest rewiring score predicted to target C43. In particular, EDDY-CTRP-PH defined the iron-sulfur (Fe-S) biogenesis gene *ISCU* as a specific, hypoxia-dependent mediator sensitive to this drug (Fig. 6A). Together, these predictions converge on the notion of a functional BRD2889-GSTP1-ISCU axis in hypoxia and PH.

In PAECs, we found that BRD2889 directly altered two C43 gene transcripts, partially reversing the decrease of *mTOR* and fully reversing the hypoxic decrease of *ISCU* (fig. S7A). In contrast, in PASMCs, the hypoxia-induced reduction in *ISCU* was unaffected by BRD2889 with no change in *mTOR* (fig. S7, B and C). Because *ISCU* was predicted as a central mediator in C43 rewiring by BRD2889 and prior studies have demonstrated that hypoxia-dependent endothelial ISCU deficiency promotes PH via repressing iron sulfur–dependent mitochondrial metabolism (*23*, *37*), we focused on endothelial cells exposed to hypoxia to define this putative BRD2889-GSTP1-ISCU axis. To determine the landscape of activities of BRD2889 in PAECs, we identified 3830 genes by transcriptional array altered by hypoxia but reversed by BRD2889 (molecular structure of BRD2889 in Fig. 6B, heatmap in Fig. 6C and table S6). By gene set enrichment analysis (table S7), a majority of these genes belonged to pathways of cell cycle, cell death, and metabolism—all relevant to ISCU biology (Fig. 6D).

In PAECs, consistent with known inhibition of GSTP1 by piperlongumine (*22*), while BRD2889 did not affect GSTP1 expression (Fig. 6, E and F), it reduced the hypoxic increase in GST enzyme activity (Fig. 6H). In parallel, BRD2889 reversed hypoxia-dependent reduction of ISCU protein levels (Fig. 6, F and G). Corresponding with the importance of ISCU deficiency in control of iron sulfur–dependent electron transport, endothelial redox state, and apoptosis in PH (*23*, *38*), in hypoxic PAECs, BRD2889 reversed the decrease in iron sulfur–dependent mitochondrial complex I activity and proliferation and reversed the increase in apoptosis (Fig. 6, I to K). Similarly, *GSTP1* knockdown in PAECs (fig. S7D) decreased GST activity (Fig. 6L), increased ISCU (Fig. 6, M and N), increased mitochondrial complex I activity (Fig. 6O), decreased apoptosis (Fig. 6P), and increased proliferation (Fig. 6Q), reversing these parameters in hypoxia. Moreover, in normoxia, GSTP1 knockdown increased oxygen consumption rate and mitochondrial respiration (fig. S7, E and F). Conversely, in PAECs, forced expression of GSTP1 (fig. S7, G to I) reduced ISCU (fig. S7, H and J), phenocopying hypoxic reduction of ISCU. Forced GSTP1 expression also increased apoptosis, reduced proliferation, and mitochondrial respiration (fig. S7, K to N), consistent with the increases of ISCU driven by BRD2889 (fig. S7, O to Q). Collectively, these observations demonstrate that PH-related up-regulation of GSTP1 promotes metabolic and mitochondrial endothelial dysfunction via control of ISCU, a process reversed by BRD2889-induced GSTP1 inhibition.

### GSTP1 promotes and depends upon ISCU glutathionylation for ameliorating metabolic endothelial dysfunction

**Fig. 7.**
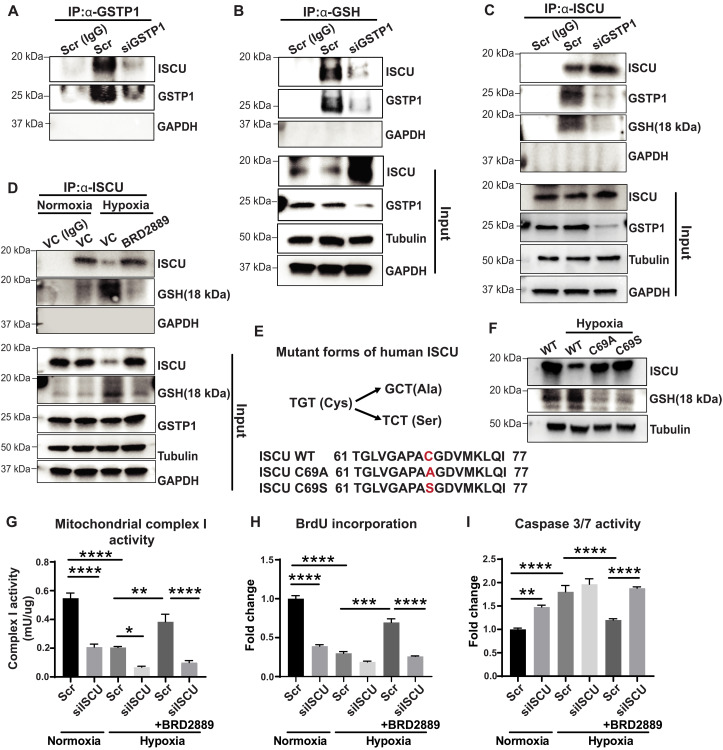
GSTP1 binds and glutathionylates ISCU to control protein stability. (**A** and **B**) PAECs treated with siGSTP1 or siRNA control (Scr) were subjected to immunoprecipitation (IP) for immunoglobulin G (IgG) control, GSTP1 (A), or glutathione (α-GSH) (B) and immunoblotted for ISCU and GSTP1. Pulldown of ISCU with GSTP1 and pulldown of ISCU and GSTP1 with GSH were observed. Such pulldown was inhibited by siGSTP1. Input for both (A and B) is shown at the bottom; glyceraldehyde phosphate dehydrogenase (GAPDH) = negative control. (**C**) Similarly treated PAECs were subjected to immunoprecipitation for IgG or ISCU followed by immunoblots of total ISCU, GSTP1, and glutathionylated ISCU (18 kDa). Pulldown of GSTP1 resulted with glutathionylated ISCU, but GSTP1 knockdown abrogated ISCU glutathionylation and this interaction. (**D**) PAECs were exposed to vehicle versus BRD2889 in normoxia or hypoxia; immunoprecipitation and immunoblotting were performed as in (C). Hypoxia reduced total ISCU but increased relative levels of glutathionylated ISCU; BRD2889 reversed these findings. (**E**) Amino acid sequences of WT and mutant forms of ISCU at Cys-69. (**F**) In human embryonic kidney (HEK) 293 cells transfected with expression plasmids encoding WT and mutant ISCU, immunoblots revealed that mutant ISCU isoforms displayed less glutathionylation. Correspondingly, hypoxia reduced WT ISCU but not C69A or C69S ISCU. (**G** to **I**) PAECs were treated with BRD2889 in hypoxia, along with siISCU versus Scr control siRNA. The actions of BRD2889 to reverse hypoxic changes of mitochondrial complex I activity (G), proliferation by BrdU incorporation (H), and apoptotic caspase 3/7 activity (I) were abolished by siISCU (*n* = 4 per group). In (A) to (F), blots are representative for *n* = 3 per group. Data are plotted as means ± SEM. Statistical significance is indicated using one-way ANOVA with Bonferroni’s multiple comparisons testing (*P < 0.05, **P < 0.01, ***P < 0.001, and ****P < 0.0001).

Given the connection of BRD2889 and GSTP1 to the control over ISCU and the known action of protein glutathionylation to regulate protein expression and activity (*39*), we hypothesized that GSTP1 controls ISCU via direct protein S-glutathionylation. In PAEC lysate, α-GSTP1 immunoprecipitation revealed that ISCU was specifically pulled down with GSTP1 (Fig. 7A) demonstrating a biochemical interaction between these proteins. Similarly, ISCU and GSTP1 were detected after immunoprecipitation with an anti-glutathione antibody (α-GSH) (Fig. 7B), indicating glutathionylation of at least one of these protein partners. Knockdown of GSTP1 resulted in a decrease of coimmunoprecipitated ISCU, suggesting control of ISCU glutathionylation by GSTP1. To garner direct evidence of these interactions, α-ISCU immunoprecipitation was performed, again demonstrating specific GSTP1 pulldown with ISCU (Fig. 7C). In this case, a glutathionylated form of ISCU was prominently detected (α-GSH immunoblot after pulldown). However, with GSTP1 knockdown, α-ISCU immunoprecipitation revealed a concomitant reduction of GSTP1 pulldown and glutathionylated ISCU in favor of nonglutathionylated ISCU. Together, these data prove that GSTP1 interacts with ISCU to control its level of protein glutathionylation.

We sought to define the functional role of BRD2889 on ISCU glutathionylation (Fig. 7D and fig. S8, A to C). In hypoxic PAECs when ISCU levels were decreased, α-ISCU immunoprecipitation revealed an increase of ISCU glutathionylation (α-GSH immunoblot after pulldown) as compared with normoxic cells (Fig. 7D). However, with BRD2889 treatment, ISCU levels were increased, accompanied by a converse reduction of glutathionylation, phenocopying the results of GSTP1 knockdown and indicating that glutathionylation controls ISCU expression. Inhibition of proteasomal degradation using MG132 reversed the hypoxic down-regulation of ISCU without affecting GSTP1 or glutathionylation (fig. S8, A to C), suggesting that ISCU degradation at least partially controls steady state levels in hypoxia and is dependent upon glutathionylation. Using the dbPTM-protein posttranslational modification tool (*40*), the cysteine residue Cys-69 in human ISCU was predicted as a specific site of S-glutathionylation. To explore the role of Cys-69 in ISCU glutathionylation, two ISCU mutants were generated converting this residue to serine (ISCU 69C/S) or alanine (ISCU 69C/A) (Fig. 7E) and thus abolish any putative glutathionylation at this site. After transfection and forced expression of either WT of mutant ISCU in human embryonic kidney (HEK) 293 cells (Fig. 7F), WT ISCU was decreased, but ISCU glutathionylation was increased in hypoxia as compared with normoxia. Conversely, in comparison to WT ISCU, both mutant ISCU proteins were increased in hypoxia, while glutathionylation was decreased, thus offering direct evidence that glutathionylation at Cys-69 controls ISCU expression particularly in hypoxia. Last, to determine whether ISCU up-regulation is essential for BRD2889’s endothelial actions, hypoxic PAECs were treated with BRD2889 during forced siRNA knockdown of *ISCU* (fig. S7, O to Q). BRD2889 did not reverse the hypoxia-induced decrease in mitochondrial complex I activity and proliferation and did not reverse the hypoxic increase in apoptosis (Fig. 7, G to I). Thus, these results define the crucial role of ISCU in mediating the activity of BRD2889 in rescuing endothelial dysfunction in PH.

Beyond pure hypoxic exposure alone, an appreciation is advancing of the mechanistic connections of ISCU specifically with IL-6 (*41*), suggesting the importance of hypoxia and IL-6 together in controlling ISCU-dependent pathophenotypes. Thus, in PAECs exposed to a combination of recombinant IL-6/soluble IL-6 receptor (sIL-6R) and hypoxia, BRD2889 reversed the increase in GSTP1 activity and reversed the decrease in ISCU expression (fig. S8, D to F). IL-6/sIL-6R + hypoxia treatment also induced PH-related inflammatory gene transcripts; BRD2889 normalized this up-regulation in PAECs (fig. S8G). Consistent with findings under hypoxia, BRD2889 also partially rescued mitochondrial complex I activity, reduced apoptosis, and increased proliferation in IL-6/sIL-6R + hypoxia–exposed PAECs (fig. S8, H to J). In contrast, in IL-6/sIL-6R + hypoxia–exposed PASMCs, BRD2889 failed to rescue ISCU or alter GST activity (fig. S8, K to M). BRD2889 did not affect the IL-6/sIL-6R + hypoxia–induced alterations of PASMC mitochondrial complex I activity and proliferation, and the modest alterations of PASMC apoptosis were only subtly changed by BRD2889 (fig. S8, N to P). Consistent with these cell type–specific differences, in PASMCs, GSTP1 knockdown (fig. S8, Q and R) also did not alter GST activity (fig. S8S). Together, in endothelial but not smooth muscle cells, GSTP1 primarily controls GST activity and ISCU and is particularly active across inflammatory and hypoxic triggers of PH.

### The GSTP1-ISCU axis is active in human PH, and BRD2889 improves existing PAH across multiple PAH rodent models

**Fig. 8.**
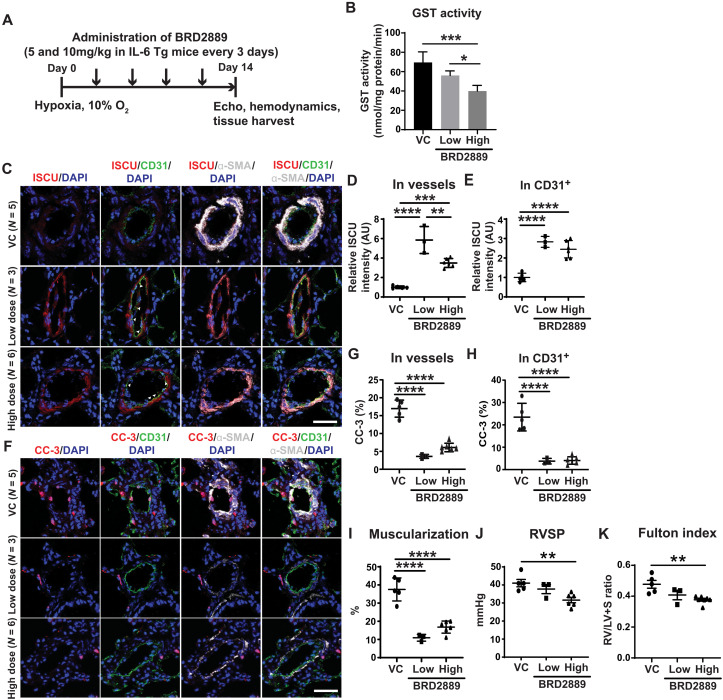
BRD2889 reverses PAH in chronically hypoxic IL-6 Tg mice. (**A**) Transgenic IL-6 Tg mice were exposed to chronic hypoxia for 14 days, and BRD2889 or VC was administered (5 mg/kg, low and 10 mg/kg, high) every 3 days by IP from day 0 (*n* = 3 to 8 per group). (**B**) BRD2889 decreased lung GST activity in a dose-dependent manner. (**C** to **H**) By immunofluorescence staining and quantification of ISCU (C to E) and cleaved caspase-3 (CC-3) expression (F to H) in whole vessels, BRD2889 increased ISCU and decreased CC-3, particularly in CD31^+^ endothelium (white arrowheads) (C). (**I** to **K**) BRD2889 reduced arteriolar muscularization (I), RVSP (J), and Fulton index (RV/LV + S; K). Data are plotted as means ± SEM. Scale bars, 50 μm. Statistical significance is indicated using one-way ANOVA with Bonferroni’s multiple comparisons testing (*P < 0.05, **P < 0.01, ***P < 0.001, and ****P < 0.0001).

To go beyond the limitations of cultured cell data and determine the relevance of the GSTP1-ISCU axis in human PH, in situ staining of pulmonary arterioles of WSPH groups 1 and 3 PH patients revealed a reduction of ISCU and increase of GSTP1 in CD31^+^ endothelial cells compared with non-PH patients (fig. S9, A to D, and table S5). Total GST enzyme activity from whole lung of both WSPH groups 1 and 3 PH patients was also increased compared with non-PH patients (fig. S9E). To determine the effects of BRD2889 on this axis and on PH in vivo, low (5 mg/kg) and high (10 mg/kg) doses of BRD were administered in a disease reversal protocol to a group 1 PAH mouse model (hypoxic IL-6 Tg mice), which also demonstrated increased lung GST activity with PAH (fig. S9F). Serial drug dosing was initiated after IL-6 Tg mice were manifesting disease but before hypoxic exposure (Fig. 8A). No differences in left ventricular function or heart rate (fig. S9, G to J) were observed in BRD2889 versus vehicle-treated mice. A dose-dependent reduction of lung total GST enzyme activity was observed across low to high BRD2889 (Fig. 8B). Via in situ staining of arterioles, both BRD2889 doses rescued ISCU, particularly in CD31^+^ endothelium (Fig. 8, C to E). In response, PAH manifestations were improved, including a reduction of downstream endothelial apoptosis (Fig. 8, F to H), pulmonary arteriolar remodeling (Fig. 8, C, F, and I), and a dose-dependent reduction of RVSP and Fulton index (Fig. 8, J and K).

**Fig. 9.**
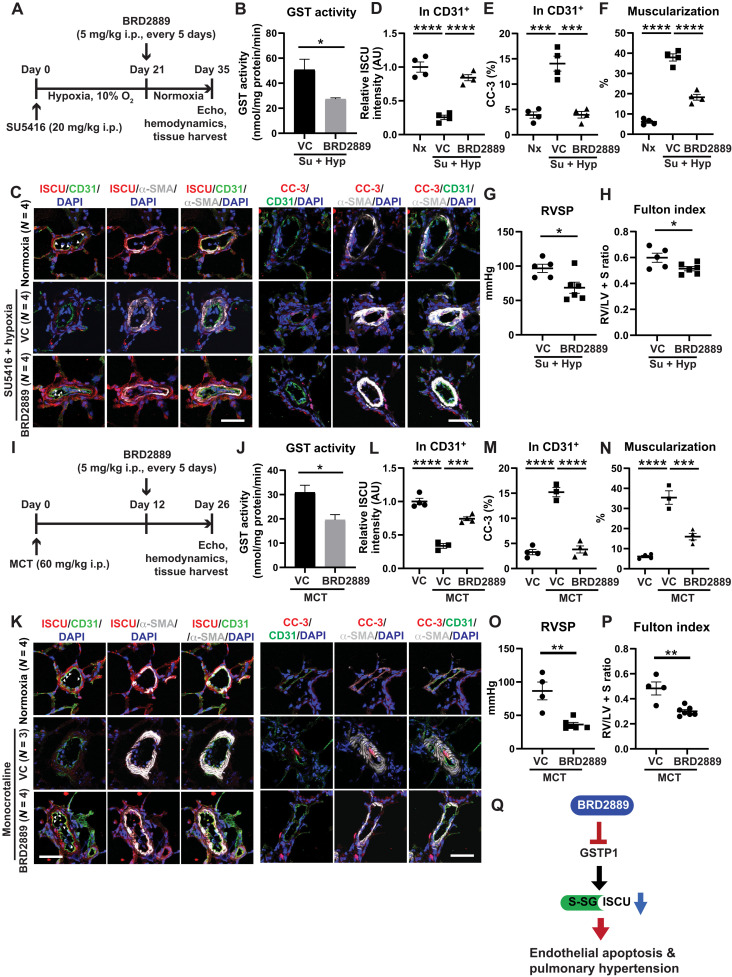
BRD2889 increases ISCU, reduces endothelial apoptosis, and reverses PAH in multiple PAH rat models. (**A**) Sprague-Dawley rats were administered SU5416 intraperitoneally (20 mg/kg) followed by hypoxia for 21 days to promote PAH. Rats were then treated with BRD2889 versus vehicle control by intraperitoneal injection (5 mg/kg) every 5 days for the next 2 weeks in normoxia (*n* = 4 to 6 per group). (**B**) BRD2889 decreased lung GST activity. (**C** to **E**) By immunofluorescence staining and quantification of ISCU (C and D) and cleaved caspase-3 (CC-3) expression (C and E) in CD31^+^ endothelium, BRD2889 increased ISCU and decreased apoptotic CC-3, notably in CD31^+^ endothelium (white arrowheads). (**F** to **H**) BRD2889 reduced arteriolar muscularization (F), RVSP (G), and Fulton index (RV/LV + S; H). (**I**) Sprague-Dawley rats were administered MCT intraperitoneally (60 mg/kg) to promote PAH within 26 days. Rats were treated with BRD2889 versus vehicle control by intraperitoneal injection (5 mg/kg) every 5 days at days 12 to 26 after MCT injection (*n* = 3 to 7 per group). (**J**) BRD2889 decreased lung GST activity. (**K** to **M**) By immunofluorescence staining and quantification of ISCU (K and L) and cleaved caspase-3 (CC-3) expression (K and M), BRD2889 increased endothelial ISCU (white arrowheads) and decreased apoptotic CC-3. (**N** to **P**) BRD2889 reduced arteriolar muscularization (N), RVSP (O), and Fulton index (RV/LV + S; P). Data are plotted as means ± SEM. Scale bars, 50 μm. Statistical significance is indicated using Student’s *t* test for (B) and (J) and one-way ANOVA with Bonferroni’s multiple comparisons testing in the remaining panels (*P < 0.05, **P < 0.01, ***P < 0.001, and ****P < 0.0001). (**Q**) Cartoon summarizing model of actions of BRD2889 on GSTP1, ISCU glutathionylation (S-SG) and expression, endothelial apoptosis, and PH.

Similarly, BRD2889 was administered in a disease-reversal dosing protocol in the same two PAH rat models tested for I-BET762—MCT and SU5416-hypoxic rats (Fig. 9, A and I). In both rat models, such dosing reduced total lung GST enzyme activity (Fig. 9, B and J) without significant alterations of heart rate (fig. S10, A and J) or aortic pressure (fig. S10, E and K). Echocardiographic assessment after BRD2889 dosing in SU5415-hypoxic rats demonstrated no alteration of left ventricular function after drug dosing (fig. S10, B to D). Notably, three SU5416-hypoxic PAH rats treated with drug displayed accumulation of mild ascites. However, by quantitative reverse transcription polymerase chain reaction (RT-qPCR) transcript screening, there was no indication of overt tissue toxicities in either model (fig. S10, F to I and L to P). In both models, BRD2889 increased endothelial ISCU (Fig. 9, C, D, K, and L) while reducing endothelial apoptosis (Fig. 9, C, E, K, and M), pulmonary vascular muscularization (via α-SMA stain; Fig. 9, F and N), RVSP (Fig. 9, G and O), and Fulton index (Fig. 9, H and P). Therefore, guided by EDDY-based predictions linking BRD2889 to ISCU, these findings establish BRD2889 as a potent repurposed therapy that reduces endothelial metabolic dysfunction, thus driving improvements of histologic and hemodynamic manifestations of across multiple PAH rodent models (Fig. 9Q).

## DISCUSSION

**Fig. 10.**
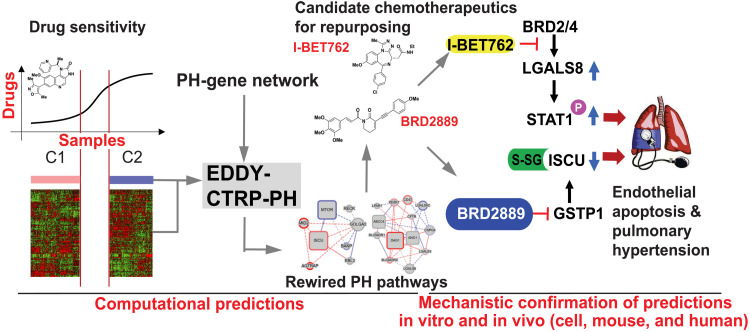
EDDY-CTRP-PH: An in silico tool to map a landscape of cancer drug mechanisms in rare noncancerous conditions such as PH. Cancer therapies are considered for rare noncancerous diseases like PH, but effective computational screening is lacking. Via transcriptomic differential dependency analyses leveraging parallels between cancer and PH, we mapped a landscape of cancer drug functions dependent upon rewiring of PH gene clusters. Experimental confirmation across independent predictions identified drug-gene axes central to endothelial dysfunction and therapeutic priorities for PH. These results establish a network dependency platform to redefine cancer drugs for use in rare and emerging noncancerous conditions such as PH.

In this study, the computational strengths of differential dependency analysis were leveraged to develop EDDY-CTRP-PH as a platform to predict the landscape of cancer drug functions controlling rare noncancerous conditions such as PH. BET inhibitors and BRD2889 separately were predicted and demonstrated experimentally to modulate endothelial LGALS8 and GSTP1-ISCU, respectively, under hypoxic and inflammatory conditions, thus controlling PH in vivo. The results pinpoint specific compounds for future therapeutic repurposing in endothelial pathobiology across multiple PH subtypes. More broadly, these findings offer wide-ranging implications for the advancement of computational network pharmacology and repurposing of drugs from cancer to other rare and often neglected conditions of health and disease (Fig. 10).

Drug repurposing has been viewed as an attractive method for reducing the cost and time of drug development, particularly for rare diseases like PH where investment is lower than other prevalent diseases (*42*). Relevant to the COVID-19 pandemic, repurposing has also been pursued for emerging diseases where prior knowledge of pathogenic target genes or pathways can be used as a linchpin for system-wide predictions of therapeutic drug activity (*43*). Here, the computational strengths in gene dependency analyses of EDDY-CTRP-PH coupled with the vast cancer cell data answer an unmet need for a rapid and system-wide method to identify therapies for rare diseases, such as PH, without prior knowledge of the molecular drug target of interest. As deep sequencing projects mature for PH and are continually applied to EDDY-CTRP-PH, broader predictions of drug-pathway interactions will be possible, extending beyond the existing PH gene clusters derived from curated literature searches and the known gene interactome. This may be particularly relevant for epigenetic and pleiotropic regulators such as BET inhibitors. Given the increasing appreciation of links of lung cancer specifically to PH (*9*), EDDY-CTRP-PH predictions could also be further honed by concentrating only on lung cancer cell responses to various tested compounds. Extension of advancing deep learning methods using scientific literature with computational linguistics and graph theory (*44*), epidemiologic data (*45*), and advanced network theory (*46*) with EDDY-CTRP-PH would be attractive. Such endeavors could offer predictions of cancer drug activity in diseases beyond PH, the cell type and context specificity of drugs, a pharmacologic differentiation of therapeutic versus toxic drug activities across disease contexts, and the synergistic responses to specific small molecules. Moreover, with the statistical power to map DDNs within a single blood or tissue sample via single-cell sequencing, future applications of EDDY-CTRP-PH to precision medicine could be envisioned via identification of individuals and/or disease subtypes with specific DDN profiles who are most likely to respond to repurposed cancer drugs.

Our experimental validation of EDDY-CTRP-PH also advances our understanding of complex cellular pathways in PH and offers guidance for translation of both I-BET762 and BRD2889 to PH. First, for both drugs, their therapeutic roles emphasized the importance of endothelial pathobiology in PH, with both long-term and immediate translational implications, as early human clinical trials are underway for BET inhibitors in PAH (NCT03655704). Second, EDDY-CTRP-PH identified multiple rewiring events for these molecules, particularly in the hotspot and extensively altered clusters C15 and C43. Notably, some C15 genes were previously implicated in PH supporting the accuracy of our predictions; for example, inhibition of *ABCC4* improved PH in mice (*47*) and identification of *LGALS3* (galectin-3) as a pathogenic factor in PH (*48*) and right ventricular fibrosis (*49*). In parallel, certain C43 genes have also been implicated in PH, namely, *MTOR* and its control over proliferative and survival programs (*50*). However, the large majority of functional drug-PH gene axes implicated here by EDDY have never been identified previously, offering a different scale of predictions and advancing our understanding of the layers of interconnections among seemingly disparate mechanisms.

Separately, our computational predictions and experimental work implicate LGALS8 and GSTP1 as crucial effectors of endothelial dysfunction in PH. In regard to LGALS8, prior studies characterized proinflammatory activities of this molecule in endothelium of other vascular beds (*20*), consistent with our findings of its dependence on inflammation-relevant BRD2/4. Our findings uncovered a key undiscovered role for LGALS8 as a mediator of BRD2/4 and I-BET controlling endothelial pathophysiology in PH. In delineating the connections between BET inhibitors with LGALS8, our findings specifically define a BRD-specific regulation of the LGALS8-L isoform in human PAECs, potentially reflecting the emerging role of BRD4 in splicing (*28*) and with previous reports on differential regulation of different isoforms of LGALS8 under different stimuli (*20*). Moreover, while our data implicated LGALS8 as essential for I-BET’s control of endothelial apoptosis and PH, LGALS8 did not reverse all I-BET effects (fig. S1B). This suggests the importance of other connected genes in mediating this pleiotropic drug’s actions and will be the focus of future iterations of our pipeline tailored to garner precision medicine predictions of individualized and heterogeneous responses to BET inhibitors. A putative cell type specificity and context specificity of BET inhibitors may be particularly relevant to our findings of an antiapoptotic role for I-BET762—consistent with prior results in endothelial cells (*51*) but distinct from the proapoptotic actions of other BET inhibitors in PASMCs and other PAH models (*19*). Our findings regarding LGALS8 also offer therapeutic opportunities beyond I-BET. Namely, the activity of extracellular LGALS8 to modulate the effects of I-BET762 indicates the potential of therapeutic antibodies in this space. However, unlike LGALS3 that is increased in peripheral plasma of patients with PAH (*52*), extracellular plasma LGALS8 was poorly expressed in peripheral vascular plasma of patients with PH (fig. S2A). These findings suggest the importance of paracrine, rather than endocrine, processes for LGALS8 in PH and thus the need for specific delivery to the pulmonary circulation of any putative therapy. Along those lines, our data in groups 1 and 3 PH rodent models coupled with two lines of investigation using hypoxia or IL-1β in cultured cells emphasize that LGALS8’s role transcends the subgroup heterogeneity of PH.

In parallel, the EDDY-based predictions that define GSTP1’s role in regulating ISCU also advance our understanding of protein S-glutathionylation in PH and the pulmonary endothelium, particularly in relation to oxidative stress (*39*). The hypoxia-dependent (*37*) and endocrine (*38*) activity of microRNA-210 is known to potently down-regulate ISCU transcript in PH (*23*), but our findings here reveal a more complex regulatory schema for this scaffolding protein. While S-glutathionylation has been reported in PH extensively (*53*), key regulator proteins have not been comprehensively identified. In cancer, GSTP1 has been found to be a tumor suppressor (*54*) or oncogene, depending upon the tumor of interest. Single-nucleotide variants in this gene have been associated with susceptibility to hypobaric hypoxia and high altitude pulmonary edema (*55*, *56*), often thought to be driven by compromise of the endothelial barrier function. GSTP1 mutations have also been linked to chronic obstructive pulmonary disease (*57*), a disease with clear etiologic connections to PH. Pulmonary GSTP1 is known to carry a predominant role in detoxification of toxic compounds and pollutants (*39*). While the exact relation of pollution exposure to PH is emerging (*58*), our findings of increased GSTP1 in PH may suggest a molecular mechanism for such a link.

Last, the identification of BRD2889 as a robust modulator of the GSTP1-ISCU axis in PH offers an intriguing compound and target pathway for therapeutic development. Differences between doses of BRD2889 in mice revealed differential effects on ISCU levels likely owing to cell-specific effects of the two doses used. Dosing sensitivity protocols should clarify this issue and pave the way for clinical therapeutic development. BRD2889’s parent compound piperlongumine has been tested as an anti-inflammatory and senolytic drug in select cancers (*59*) but carries distinct roles in other contexts and nontransformed cells. Notably, our EDDY-based predictions found specific PH pathway rewiring responsible only to BRD2889 but not the parent drug or other analogs, also indicating the context-specific activity of this drug class and potentially its interactions with its target GSTP1. Thus, even among drug analogs, these distinctions emphasize the power of EDDY-CTRP-PH via its efficiency and granular detail to map and compare downstream molecular drug responses. While the piperlongumine parent drug has minimal toxicity to normal, nontransformed cells, its derivatives have displayed low levels of reversible liver and kidney toxicity when administered systemically (*60*). Given the presence of ascites in some BRD2889-dosed rats, future therapeutic development of BRD2889 should assess for toxicity closely and may benefit from localized delivery to the lung, as we have described recently with poly(lactic-co-glycolic) acid (PLGA) microparticles (*61*), to avoid any putative systemic side effects. In addition, future work to integrate EDDY-CTRP-PH with a structural analytic pipeline would be appealing to define potential biophysical mechanisms by which modifications of piperlongumine can be mapped to downstream pathway rewiring. Tailored development of EDDY will be valuable to determine whether the combinatorial effects of I-BET762 and BRD2889 in PAECs can be predicted and tuned.

In summary, we leveraged a computational approach with experimental validation to identify system-level molecular relationships between PH and existing cancer small-molecule drugs, resulting in predictions and proof of their therapeutic potential. These results not only offer key insights into the endothelial pathobiology in PH but also establish the validity of leveraging cancer-based transcriptomics for identifying the hidden activities of therapeutic small molecules in this disease. Hence, this work establishes the validity for a platform of computational repositioning of cancer drugs in other rare and emerging diseases that has not yet been possible.

## MATERIALS AND METHODS

### Experimental design

The goal of this study was to generate a computational-to-empirical pipeline for identifying and ranking the most robust actions of specific cancer therapeutics and revealing their disease-relevant downstream targets in an example of a rare noncancerous disease such as PH. Data sources for EDDY-CTRP-PH included CCLE (*13*), CTRP (*14*, *15*), and a specific PH gene network (table S1). Following identification of I-BET151/762 with convergent actions on PH gene cluster 15 (including the gene LGALS8), gene expression, mitochondrial redox levels, and cellular apoptosis were measured in primary human PAECs. To determine the effect of this drug on PH in vivo, C57BL/6 mice suffering from hypoxia-induced group 3 PH and two models of Sprague-Dawley rats suffering from group 1 PH (MCT exposure and SU5416-hypoxia) were treated with I-BET762. To determine the pathogenic actions of LGALS8 in PH, male and female *Lgals8^−/−^* mice were also exposed to chronic hypoxia. *Lgals8^−/−^* mice and their littermate controls were limited on the availability by breeding. Following identification of BRD2889 with actions on PH gene cluster 43 (including the gene ISCU), gene expression, ISCU glutathionylation, GSTP1-ISCU binding, and downstream phenotypes were measured in PAECs and PA smooth muscle cells. To determine the effect of this drug on group 1 PH in vivo, IL-6 transgenic C57BL/6 mice exposed to hypoxia and MCT rats and SU5416-hypoxic rats were treated with drug versus vehicle control. Hemodynamic and histologic indices were evaluated in murine models. Sample size and statistical analyses for each experiment are described below and in the figure legends; rodent studies were performed via random assignment to various experimental groups, and hemodynamic and histologic analyses were performed in a blinded fashion. Human groups 1 and 3 PH lung (table S5), nondiseased lung, and peripheral plasma were studied (*62*). Rodent numbers were chosen to achieve 0.80 power for detecting at least a 25% difference among means with an SD of 20%. The number of recruited patients was determined primarily by availability of clinically validated samples. All experimental procedures involving human tissue and blood were approved by institutional review boards at the University of Pittsburgh. Ethical approval for this study and informed consent conformed to the standards of the Declaration of Helsinki. All animal experiments were approved by the University of Pittsburgh. Key resources are summarized in table S8.

### Development of EDDY-CTRP-PH

Details of PH gene network and clustering and EDDY-CTRP-PH are described in the Supplementary Materials.

### Data and code availability

All the microarray data have been submitted to GEO (accession nos. GSE125508 and GSE160255 for I-BET and BRD2889, respectively). EDDY software is available at GitHub repository (https://github.com/dolchan/eddy-gpu). The EDDY-CTRP-PH analysis for all the clusters and small molecule is available at https://chan.vmi.pitt.edu/eddy-ctrp-ph/.

### Cell culture

Primary human PAECs and human PASMCs were purchased from Lonza (302-05A, CC-2581). Notably, the same two male donors were the source of all PAECs in the experiments shown. Two additional male donors were the source for all PASMCs in the experiments shown. Cells from these and any donors were characterized by flow cytometry for consistent expression of cell surface markers and by RNA (RT-qPCR) analysis of endothelial and smooth muscle gene expression. PAECs were cultured in EGM-2 media (CC-3121) along with supplements (CC-4133), and PASMCs were cultured in SmGM-2 culture media (Lonza, CC-3182) at 5% CO_2_ in a humidified incubator.

To assess the effect of I-BET under inflammatory conditions, cells were treated with recombinant human IL-1β (10 ng/ml; PeproTech) at about 70 to 80% confluency for 48 hours in complete media along with vehicle control [dimethyl sulfoxide (DMSO); Sigma-41639], I-BET151 (500 nM), and I-BET762 (500 nM) (Selleckchem-S2780/S7189), as indicated. Human recombinant galectin-8 (1305-GA-050) was purchased from R&D Systems and was used at a working concentration of 30 nM.

For hypoxia exposure, cells were plated in six-well cell culture plates at 1 × 10^5^ cells per well, grown for 24 hours, and placed into a normobaric hypoxia chamber (1% O_2_) for 24 hours under specific treatment conditions. Namely, for I-BET and siLGALS8 exposures, cells were either pretreated with I-BET/vehicle control or transfected with siLgals8/Scr for 24 hours. Then, they were cultured in basal media at 1% O_2_. After 24 hours, caspase activity and mitochondrial superoxide levels were quantified. To detect activation status of pSTAT1/STAT1 by immunoblot, cells were exposed to 8 hours of hypoxia.

In IL-6/sIL-6R + hypoxia experiments, a human recombinant IL-6/IL-6R alpha protein chimera (25 ng/ml; Millipore) was administered at 70 to 80% confluency for 48 hours in complete media along with vehicle control (DMSO, Sigma-41639). BRD2889 (1 μM) versus vehicle control were added, as indicated, and placed into a hypoxia chamber for 24 hours. The hypoxia chamber (modular incubator chamber) was obtained from Billups-Rothenberg Inc. (Del Mar, Calif) and placed in regulated CO_2_ incubator at 37°C.

### Animal models

For the hypoxia-induced PH mice, male C57BL/6J mice (8 weeks old) (RRID:IMSR_JAX:000664) were purchased from the Jackson Laboratory and acclimatized for 3 to 4 days in our facility. Subsequently, mice scheduled for nomoxic versus hypoxic exposure were maintained in either normoxia or a normobaric hypoxia chamber for 1 or 3 weeks (OxyCycler, Biospherix Ltd.), where consistent exposure to 10% oxygen and control for temperature and humidity were possible. For testing the activity of I-BET in hypoxia-induced PH mice, three experimental groups—normoxia + vehicle control, hypoxia + vehicle control, and hypoxia + I-BET—were used. I-BET762 (30 mg/kg; Selleckchem) (*63*, *64*) was administered by daily oral gavage for the duration of hypoxic exposure.

Generation of the SU5416-hypoxia PH rat model was described previously (*17*). Briefly, 10-week-old male Sprague-Dawley rats were intraperitoneally injected with SU5416 (20 mg/kg; Sigma-Aldrich), placed in normobaric hypoxia (10% O_2_) for 3 weeks and then transferred to normoxia for 2 weeks. During hypoxic exposure, chambers were opened twice a week for cleaning and replenishment of food and water. For I-BET762 versus vehicle control dosing, daily intraperitoneal injections (30 mg/kg Selleckchem) were administered during the final 2 weeks of normoxia. For BRD2889 versus vehicle control dosing, intraperitoneal injections every 5 days (5 mg/kg) were administered during the final 2 weeks of normoxia. Oxygen concentrations were continuously monitored with blood gas analyzers.

For the MCT PH rat model, as previously described (*62*), male Sprague-Dawley rats (10 to 14 weeks old) were injected (intraperitoneally) with MCT (60 mg/kg) versus phosphate-buffered saline (PBS) and kept for 26 days in normoxia (*n* = 4 per group). For I-BET762 versus vehicle control dosing and BRD2889 versus vehicle control dosing experiments, dosing strategies similar to those in SU5416-chronic hypoxic rats were used from days 12 to 26 after MCT injection.

I-BET was dissolved in DMSO at 100× concentration and then made into a working solution in 20% PEG400 and 80% of 1× PBS. BRD2889 was made into a working solution of 2.5% DMSO.

*Lgals8*^−/−^ C57BL/6N mouse sperm was purchased from KOMP (14305A-F8) (*65*) and reconstituted in house and genotyped, as per KOMP’s instructions. Male and female 8-week-old *Lgals8^−/−^* mice were exposed to hypoxia for 3 weeks. Littermates were used as WT control when comparing with knockout mice. Before euthanasia, echocardiography was performed as described (*62*, *66*), followed by closed-chest right heart catheterization (*67*) to measure RVSP and heart rate. For rats, invasive catheterization of the abdominal aorta was performed to quantify systemic blood pressure. Following euthanasia, right ventricle/[left ventricle + septum] (RV/LV + S) mass ratio (Fulton index) was quantified, accompanied by Tissue-Tek OCT (VWR) tissue preparation for histologic staining, as described (*62*, *66*).

Pulmonary-specific IL-6 transgenic mice (C57BL/6 background) were described previously (*68*). These mice were bred in house, and 12-week-old male transgenic mice versus control littermates were compared. Mice were intraperitoneally injected every 3 days with vehicle control (2.5% DMSO) versus BRD2889 (5 mg/kg versus 10 mg/kg), followed by exposure to normobaric hypoxia (10% O_2_;OxyCycler chamber, Biospherix Ltd., Redfield, NY) for 14 days, as described (*68*).

Animal numbers were chosen to achieve 0.80 power for detecting >25% difference among means with an SD of 20%. All animal experiments were approved by the University of Pittsburgh (IACUC). Randomization of the animals assigned to different experimental groups was achieved. Briefly, populations of animals sharing the same gender, same genotype, and similar body weight were generated and placed in one container. Then, each animal was picked randomly and assigned in a logical fashion to different groups. For example, the first one is assigned to group A, second to group B, third to group A, fourth to group B, and so forth. No animals were excluded from analyses.

### Human samples

Human group 1 PH (PAH), group 3 PH, and nondiseased lung samples as well as peripheral plasma are described in table S5 and previously (*62*). The number of recruited patients was determined primarily by the availability of clinical samples. Experimental procedures involving human tissue were approved by institutional review boards at the University of Pittsburgh. Ethical approval for this study and informed consent conformed to the standards of the Declaration of Helsinki.

### BRD2889 synthesis

BRD2889 was prepared from commercially available piperlongumine via a reported two-step procedure (α-iodination and Sonogashira coupling) and purified by silica gel chromatography followed by recrystallization (*22*).

### Transfection

Human PAECs were transfected at about 70 to 80% confluency in Opti-MEM media (Thermo Fisher Scientific) with 6.25 nM scrambled (4390843) or Lgals8 (s8158), Brd2 (s12070), Brd4 (s23901), Jak1 (s7646), Jak2 (s7651), Stat1 (s279) silencer select siRNA (Thermo Fisher Scientific), 5 nM nontarget pool (D-001810-10-05), or 5 nM GSTP1 (J-011179-07-0010) siRNA (Dharmacon, a Horizon Discovery Group) and 5 nM ISCU (J-012837-11-0020) siRNA using Lipofectamine 2000, according to the manufacturer’s instructions (Thermo Fisher Scientific). After 6 hours, Opti-MEM was replaced by endothelial growth media, and cells were analyzed 48 hours after transfection. Similarly, HEK293 cells (American Type Culture Collection no. CRL 1573) were transfected with 0.5 μg of WT-ISCU, C69S-ISCU, C69A-ISCU, or pcDNA3.1 empty vector using Lipofectamine 2000, according to the manufacturer’s instructions (Thermo Fisher Scientific). After 48-hour transfection, the cells were exposed to hypoxia for 24 hours before harvesting for cellular lysate.

### Statistical analysis

Data are represented as means ± SEM. For cell culture data, these represent three independent experiments performed in triplicate. The normality of data distribution was confirmed by Shapiro-Wilk testing. For normally distributed data, a two-tailed Student’s *t* test was used for comparisons between two groups. For comparisons among groups, one-way analysis of variance (ANOVA) and post hoc Bonferroni testing were performed. A *P* value less than 0.05 was considered significant. Detailed descriptions of other standardized and published approaches are provided in the Supplementary Materials.

## Supplementary Materials

### This PDF file includes:

Supplementary Materials and Methods Figs. S1 to S10 Legends for tables S1 to S4, S6 and S7 Tables S5 and S8 References

## Notes

View/request a protocol for this paper from *Bio-protocol*.
