# Supplementary material for: Computational repurposing of therapeutic small molecules from cancer to pulmonary hypertension
Source: Sci Adv. 2021 Oct 20;7(43):eabh3794. doi: 10.1126/sciadv.abh3794 (PMC8528428; doi:10.1126/sciadv.abh3794)
Supplement: Supplementary file 1 — Supplementary Materials and Methods Figs. S1 to S10 Legends for tables S1 to S4, S6 and S7 Tables S5 and S8 References [file sciadv.abh3794_sm.pdf]

Science Advances
AAAS

# Supplementary Materials for

## Computational repurposing of therapeutic small molecules from cancer to pulmonary hypertension

Vinny Negi, Jimin Yang, Gil Speyer, Andres Pulgarin, Adam Handen, Jingsi Zhao, Yi Yin Tai, Ying Tang, Miranda K. Culley, Qiujun Yu, Patricia Forsythe, Anastasia Gorelova, Annie M. Watson, Yassmin Al Aaraj, Taijyu Satoh, Maryam Sharifi-Sanjani, Arun Rajaratnam, John Sembrat, Steeve Provencher, Xianglin Yin, Sara O. Vargas, Mauricio Rojas, Sébastien Bonnet, Stephanie Torrino, Bridget K. Wagner, Stuart L. Schreiber, Mingji Dai, Thomas Bertero, Imad Al Ghouleh, Seungchan Kim, Stephen Y. Chan*

*Corresponding author. Email: chansy@pitt.edu

Published 20 October 2021, *Sci. Adv.* **7**, eabh3794 (2021)
DOI: 10.1126/sciadv.abh3794

**The PDF file includes:**

Supplementary Materials and Methods
Figs. S1 to S10
Legends for tables S1 to S4, S6 and S7
Tables S5 and S8
References

**Other Supplementary Material for this manuscript includes the following:**

Tables S1 to S4, S6 and S7

## SUPPLEMENTARY MATERIALS AND METHODS

*Construction of the PH-extended gene network:* Adapted from a prior version of the PH gene network (*62*), the network was constructed with a set of 416 seed genes which were identified as related to PH from a contemporaneous curated literature review. Functional interactions for the network were collected from DIP (*69*), BioGRID (*70*), CORUM (*71*), InnateDB (*72*), IntAct (*73*), MINT (*74*), and MatrixDB (*75*). The total union of interactions from all these databases was referred to as the consolidated interactome (CI).

Not all 416 seed genes were interconnected into a single largest connected component (LCC). In order to ensure inclusion of all the seed genes within a single LCC, intermediate non-seed genes were included into an expanded network. The process began by finding the LCC seeds which would form the base network. Intermediate non-seed genes were added into the network if they would connect one of the yet to be included seeds into the existing network. This process was repeated until the maximum number of possible seeds was included. It included a vast majority of the original PH genes but yielded a very dense network of interactions. To decrease the network's density in order to ensure meaningful computational predictions, interactions were pruned based on the p-values assigned to each interaction computed using Monte Carlo simulation. 10,000 random networks were generated using random sets of 416 seed genes. The p-value of each interaction corresponded to its frequency of appearance in these random networks. Interactions were removed from the PH network in order of descending p-value until the point where removing any additional interactions would disconnect one of the seed genes. This process generated the pulmonary hypertension (PH)-extended network, consisting of 2,946 interactions among 747 genes. Finally, the PH-extended network was subdivided into 55 groups of related genes (**Table S1**), using the Map Equation (*76*) which clusters and divides genes based on density of interactions and ready to feed into EDDY-CTRP-PH.

*Development of EDDY-CTRP-PH pipeline:* First, RNA expression data from the CCLE was quantified using the transcript expression quantification tool Salmon (*77*), $\log_2$-transformed, and quantized using median absolute deviation (MAD) into under-expressed, intermediate, and over-expressed levels. Second, drug sensitivity data from the CTRP was used to group sensitive, intermediate and non-sensitive CCLE cell lines. Third, the PH-related gene clusters allowed the computational interrogation of gene co-expression to be constrained to subgroups of interest. Finally, previously known interactions mined from Pathway Commons 2 (www.pathwaycommons.org) were utilized as prior knowledge in EDDY analysis with a weight of 0.5, decreasing false-discovery rate without overly reducing differential dependency detection sensitivity (*78*).

Once these input sources were assembled, EDDY (*11*) was used to construct graphs for gene dependencies in cells sensitive and resistant to a given small molecules, where edges between nodes were defined by a pairwise independence test ($\chi^2$ test) of gene expression, with known interactions (edges) given a priority. By repeated resampling of each group (sensitive vs. resistant cells), multiple unique networks were constructed for each group, and upon scoring, each group was characterized with a network likelihood distribution. The significance of the divergence between the two distributions was assessed via permutation test. Thus, statistically significant differential rewiring among gene networks between cells resistant and sensitive to a small molecules was catalogued. These clusters were then visualized with a differential dependency network (DDN). Across 368 compounds, 810 cell line responses presented different sensitive and non-sensitive groupings, which, in turn, yielded specific significant PH cluster lists from EDDY. Of note, even when the same cluster was found to be statistically significant for two different compounds, its DDN often displayed substantially different wiring. For visualization, each characteristic line in DDNs indicated the identified relationship between nodes (genes): drug-sensitive (red), drug-resistant (blue), and both (gray) as well as known interactions with

directionality (solid edges +/- arrow) and previously unknown statistical dependencies (dashed edges).

For each DDN, genes important to the integrity of the network were identified by network analysis. Namely, the betweenness-centrality metric assessed a node's essentiality within a network (*79*) and was visualized in the condition-specific network through the node size. In each DDN, essentiality mediators were identified as those with the highest (top 10%) betweenness-centrality difference between the two condition-specific networks and the size of the nodes in each DDN represented the betweenness-centrality difference. The condition-specific rewiring metric identified genes with a significant proportion of condition-specific edges assessed against the binomial distribution of these edges across the entire graph. In each DDN, these specificity mediators were identified, highlighting particularly highly altered roles between conditions. Both essentiality and condition-specific mediators were indicated by square nodes.

Furthermore, in order to shortlist, the small molecules and clusters were ranked based on three criteria: 1) "average" p-value ($\bar{p}_{\cdot j}$ and $\bar{p}_{i\cdot}$; see the description below for detail), 2) frequency of mediator involvement, and 3) number (or frequency) of significantly rewired clusters for a given small molecule and number (or frequency) of small molecules linked to rewiring of a given cluster. Mathematically, let $C = [c_{i,j}]$, where $c_{i,j} = 1$ if a PH cluster $P_i$ is enriched for differentially dependencies for a drug $D_j$ with a p-value $p_{i,j}$; and $c_{i,j} = 0$ otherwise. Also, let $M = [m_{ij}]$, where $m_{ij}$ is the number of mediator genes identified for a PH cluster $P_i$ and a drug $D_j$. We also denote $m_{i\cdot}$ and $m_{\cdot j}$ as the number of *unique* mediator genes for a PH cluster $P_i$ and the number of unique mediator genes for a drug $D_j$, respectively. For each drug $D_j$, we also denote $N_C(D_j) = \sum_i c_{i,j}$ as the number of PH clusters associated with the drug $D_j$ and $\bar{p}_{\cdot j} = -\frac{1}{N_C(D_j)} \sum_i \log_{10} p_{ij}$ as the "average" p-value for the drug $D_j$. Finally, for each PH cluster $P_i$, we denote $N_D(C_i) = \sum_j c_{i,j}$ as the number of drugs the PH cluster is enriched for differentially dependencies, and $\bar{p}_{i\cdot} =$

$-\frac{1}{N_D(C_i)}\sum_j \log_{10} p_{ij}$ as the "average" p-value for the PH cluster $P_i$. We then rank a drug for $N_C(D_j)$, $m_{\cdot j}$ (both in descending order) and $\bar{p}_{\cdot j}$ (in ascending order), and denote them $r_C(D_j)$, $r_m(D_j)$, and $r_p(D_j)$, respectively. As shown in **Tables S1-S2**, ultimately, the overall rank score of a drug was the sum of these three ranks, ($r(D_j) = r_C(D_j) + r_m(D_j) + r_p(D_j)$), and a drug was ranked based on the overall rank score (ascending order), $r(D_j)$. Similarly, we ranked a PH cluster for $N_D(C_i)$, $m_{i\cdot}$ (both in descending order) and $\bar{p}_{i\cdot}$ (in ascending order), and denoted them $r_D(C_i)$, $r_m(C_i)$, and $r_p(C_i)$, respectively. The overall rank score of a PH cluster was the sum of these three ranks ($r(C_i) = r_D(C_i) + r_m(C_i) + r_p(C_i)$), and a PH cluster was ranked based on the overall rank score (ascending order), $r(C_i)$.

*Gene set enrichment analysis:* Gene set enrichment analysis (GSEA) was performed using fgsea (*80*), adapted from the original description (*81*). Gene Ontology (*82, 83*) biological processes were retrieved from the molecular signatures database (MSigDB) (*84, 85*).

*Global transcriptome analysis*: Total RNA isolated from PAECs treated with IL-1β and vehicle or I-BET762 using RNeasy kit (Qiagen) according to manufacturer's instruction and were analyzed to determine global transcriptome expression using Affymetrix Clariom S array. The gene expression was normalized with Robust Multiarray Analysis (RMA). For BRD2889, the same approach was employed using Affymetrix Clariom S array on RNA isolated from PAECs treated with BRD2889 or vehicle and subjected to either hypoxia or normoxia. Differentially expressed genes were defined as any gene for which the FDR adjusted p-value was below 0.05 (n=3/grp). Reversed genes were further defined as those genes that were differentially expressed in both (hypoxia versus normoxia) and in (hypoxia + BRD2889 versus hypoxia) + vehicle as well as were regulated in opposite directions. Similarly, for I-BET that were differentially expressed in IL-1β+VC versus control and IL-1β+I-BET versus IL-1β+VC and were regulated in opposite directions. The

data for both I-BET and BRD2889 have been submitted to the NCBI Gene Expression Omnibus with accession numbers GSE125508 and GSE160255, respectively.

*RT-qPCR analysis:* Cells were lysis in 1 ml of QiaZol reagent (Qiagen). Total RNA content was extracted using the RNeasy kit (Qiagen), according to the manufacturer's instructions. Total RNA concentration was determined using a BioTek Synergy multimode plate reader. Messenger RNAs were reverse transcribed to generate cDNA using the Multiscript RT kit (Thermo Fisher Scientific). cDNA for specific gene targets was amplified and quantified via fluorescently-labeled Taqman primer sets and Taqman fast advanced master mix (Thermo Fisher Scientific) using an Applied Biosystems QuantStudio 6 Flex Real-Time PCR System. Taqman primers used for RT-qPCR are listed in **Table S8.**

*Protein stability assay*: The proteasome inhibitor MG132 (M7449, Sigma) was stored at -20°C at a concentration of 10 mM diluted in dimethyl sulfoxide (DMSO). PAECs were exposed to MG132 (5μM) vs. DMSO vehicle control for 2hr under hypoxia, prior to harvesting of cellular lysate for immunoblotting.

*Proximity ligation assay:* A Duolink PLA assay was developed according to the manufacturer's instructions (Sigma Aldrich, DUO96020). Briefly, PAECs were grown on coverslips, and then blocked for 1 hr with Duolink Blocking Solution. Samples were then stained with the indicated antibodies (anti-Integrin α3 (E-8), Santa Cruz Biotechnology, sc-393298; anti-Galectin 8/Gal-8 antibody, Abcam, ab109519; and normal Goat IgG Control, R&D Systems, AB-108-C) overnight at 4°C, diluted 1:100 in Duolink Antibody Diluent. PLUS and MINUS secondary PLA probes against rabbit and mouse IgG in Duolink® Antibody Diluent were added, and the cells were incubated at 37°C for 1 h with, followed by incubation with ligation mix for 30 min at 37°C. Amplification mix was then applied for 100 min at 37°C. The coverslips were mounted on microscope slides with Duolink Mounting Medium with Dapi, and the cells photographed under a fluorescence microscope.

*Mitochondrial function assays:* Baseline mitochondrial function and mitochondrial stress response were measured by oxygen consumption rate (OCR) using the Cell Mito Stress Kit with a XF24 extracellular flux analyzer (SeaHorse Bioscience, North Billerica, MA) following manufacturer's instructions. Briefly, 30,000 cells per well were grown overnight and, for OCR measurements, washed with XF assay medium (SeaHorse Bioscience) containing 10mM Glucose, 1mM Sodium-Pyruvate and 2mM L-Glutamine set to pH=7.40. OCR was measured over time at baseline and following consecutive injections of 1µM Oligomycin, 1µM FCCP and a mix of 1µM Rotenone + 1µM Antimycin A. Following the manufacturer's instructions, maximal mitochondrial respiration was determined as OCR following FCCP (Carbonylcyan ideptrifluoromethoxyphenylhydrazone) injection. Spare respiratory capacity was defined as ΔOCRFCCP-baseline and mitochondrial ATP production as ΔOCR Baseline-Oligomycin. For extracellular acidification rate (ECAR) measurements cells were washed in glucose-free XF base medium (Seahorse Bioscience) containing 2mM L-Glutamine at pH 7.35. ECAR was determined after serial injections with 10mM D-Glucose, 1µM Oligomycin and 100mM 2-Deoxyglucose.

*Mitochondria isolation*: Mitochondrial fraction from cultured PAECs with drug and siGSTP1 using the Mammalian Mitochondria Isolation Kit for Tissue & Cultured Cells (Biovision, Inc.) according to the manufacturer's instructions.

*Measurement of mitochondrial complex activities:* Complex I activity of isolated mitochondria were measured using Complex I Enzyme Activity Microplate assay kit (Abcam, ab109721) according to the manufacturer's protocol. These enzymes were captured within the wells of the microplate coated by the corresponding complex enzyme antibody, and activities were detected colorimetrically.

*Flow cytometry detection of mitochondrial superoxide:* For measuring mitochondrial superoxide, cells were incubated with 5µM of MitoSOX Red mitochondrial superoxide indicator (Thermo Fisher Scientific) for 10 min in 37$^{o}$C $CO_2$ incubator. Next, cells were washed with 1X PBS,

trypsinized, and fluorescence (488nm/580nm) was measured on flow analyzer (BD LSR FORTESSA or BD LSRII) using BD FACSDIVA software.

*Cellular apoptosis:* Caspase-3/7 activity was quantified using the Caspase-Glo 3/7 Assay (Promega), according to manufacturer's instructions. Caspase-3/7 activity was normalized to total protein content determined by BCA method (Thermo Fisher Scientific).

*BrdU growth assay:*. Cell proliferation was assayed relative to day 0 using a BrdU Cell Proliferation Assay Kit (#6813, Cell Signaling) according to the manufacturer's protocol after incubation for 2 h with BrdU.

*Lentivirus production*: HEK293 cells were transfected using Lipofectamine 2000 (Invitrogen) with indicated lentiviral plasmids along with packaging plasmids (pPACK, System Biosciences), according to the manufacturer's instructions. Virus was harvested, sterile filtered (0.22 μm), titered via serial dilution and visualization of GFP expression, and utilized for subsequent infection of PAECs for gene transduction.

*Immunoblot and densitometry:* Cells were lysed in RIPA buffer (Sigma) along with 1X protease inhibitor cocktail (Sigma). Protein lysate was resolved by gradient 4%-15% SDS-PAGE gels and transferred onto a 0.2μm PVDF membrane (Bio-Rad). Membranes were blocked in 5% non-fat milk in 1X PBST buffer for one hour at room temperature. Later, they were incubated in the presence of the primary antibody overnight at $4^{o}C$ and then appropriate HRP-conjugated secondary antibodies (Life Technologies). The following antibodies were used: human LGALS8 (AF1305, RnD Systems), mouse/rat Lgals8 (ab69631, Abcam), pSTAT1 (ab29045, Abcam) and STAT1 (ab47425, Abcam), GSTP1 (ab153949, Abcam), ISCU (14812-1-AP, Prointech), Glutathione (ab19534, Abcam), HIF2A (NB100-122, Novous), α-Tubulin (CP06, Millipore sigma), and ACTB (sc-47778, Santa Cruz). The immunoreactive bands were visualized with the Chemidoc XRS+ system (Bio-Rad) using SuperSignal West Femto chemiluminescent substrates

(Thermo Fisher Scientific). The images were later quantified using AlphaEaseFC software (Alpha Innotech).

*Plasmids:* To construct a lentiviral plasmid carrying a GSTP1 transgene, oligonucleotides were synthesized by Integrated DNA Technologies. The primer sequences were as follows: forward, CGGGATCCCATGCCGCCCTACACCGTGGTG and reverse, AGTTTAGCGGCCGCGTCAGTGGTGGTGGTGGTGGTGCTGTTTCCCGTT. The GSTP1 PCR products were amplified from a purchased plasmid (RG2030086, OriGene Technologies) and cloned into the vector pCDH-CMV (CD511B-1, System Biosciences) at the *BamH*I(underlined)/*Not*I(underlined) sites. Appropriate GSTP1 sequence was confirmed in comparison to sequence reference GenBank: NM_000852.4. The control lentiviral vector expressing GFP was used as a negative control.

*Site-directed mutagenesis*: The full-length human ISCU sequence (NCBI accession number BC061903) was used as the reference sequence for wildtype (WT) ISCU and cloned using the primers:

forward, CCGGAATTCGACTACAAAGACGATGACGACAAGATGGCGGCGGCTGGGGCTTTC reverse, CGGGATCCCGTCATTTCTTCTCTGCCTCTCCTTTTTTGGGTTCTTG.

Via the QuikChange Primer Design tool (Agilent Technologies), mutagenesis forward primers and their reverse complement were then designed to replace the 69th cysteine residue position of ISCU to serine (5′- CATTACGTCACCACTTGCTGGAGCCCCCA -3′) or alanine (5′-TGCCGTCGACCCCATTGACGGCAGCACGCTGATGAGCAAG-3′). Specifically, the codon TGT for Cys69 was replaced by TCT for serine and by GCT for alanine. Site directed mutagenesis was carried out using the QuikChange Lightning Site-Directed Mutagenesis kit (Agilent Technologies, Santa Clara, CA). XL10-Gold Ultracompetent Cells were transformed with the PCR products. The cells were plated on LB agar containing ampicillin (100 μg/mL) and kept at 37°C overnight.

Plasmids were extracted and transformed into BL21 (DE3) competent cells for protein expression and purification. Mutagenesis was confirmed by sequencing and cloned into the vector pCDH-CMV-GFP (CD511B-1, System Biosciences) at the *EcoR*I(underlined)/*BamH*I(underlined) sites.

*LGALS8 (Galectin-8) ELISA:* Human LGALS8 ELISA quantification was performed using a kit (Sigma-Aldrich, RAB1050) according to the manufacturer's instructions.

*Measurement of GST activity:* The GST activity from lung tissue and PAECs was assayed using GST assay kit (Cayman, MI, USA) per the manufacturer's instructions.

*Rodent echocardiography:* Echocardiography was performed using a 15-45MHz transthoracic transducer and a Visual Sonics Vevo 3100 system (Fujifilm). Inhaled isoflurane anesthesia was used at 2% in 100% O2 during positioning and hair removal and then decreased to isoflurane 0.8% during imaging. Digital echocardiograms were analyzed off-line for quantitative analysis as previously described (*62*).

*Immunoprecipitation:* PAECs were transfected with the indicated combinations of siRNAs by Lipofectamine 2000 (Thermo Fisher Scientific) according to the manufacturer's instructions and/or treated with BRD2889 (1μM, 24h). Whole cell extract (200 μg) of cells were immunoprecipitated with IgG control (1 μg, Abcam), anti-GTSP1 Ab (1μg, Abcam), anti-GSH Ab (1μg, Abcam), or anti-ISCU (1μg, Abcam) and the immune complexes were pulled down with protein A/G agarose beads (Santa Cruz, sc-2003). After extensive washing, the immunoprecipitated proteins were analyzed by immunoblotting with the indicated antibodies.

*Immunofluorescent staining:* Cryostat sections were cut from OCT-embedded lung tissues at 5-10 μm and mounted on gelatin-coated histological slides. Slides were thawed at room temperature for 10-20 min and rehydrated in wash buffer for 10 minutes. All sections were blocked in 10% donkey serum and exposed to primary antibody and Alexa 488, 568 and 647-conjugated secondary antibodies (Thermo Fisher Scientific). The following primary antibodies were used:

Lgals8 (Ab69631, Abcam; 1/200), IL-1β (ab9722, Abcam; 1/200), GSTP1 (ab153949, Abcam; 1:100), ISCU (14812-1-AP, Proteintech; 1:100), cleaved caspase 3 (cs-9661, Cell Signaling; 1/400), α-SMA (F3777, Sigma; 1/200) and CD31 (ab7388, Abcam; 1/200). Images were obtained using Nikon A1 confocal microscope with 40X objective. Small pulmonary vessels (10 vessels/section) that were not associated with bronchial airways were selected for analysis. Intensity of staining was quantified using ImageJ software (NIH). Degree of pulmonary arteriolar muscularization was assessed in OCT lung sections stained for α-SMA by calculation of the proportion of fully and partially muscularized peripheral (<100 μm diameter) pulmonary arteriole to total peripheral pulmonary arterioles (*62*).

## SUPPLEMENTAL FIGURE LEGENDS

**Figure S1. I-BET alters C15 and protects from endothelial dysfunction by directly regulating LGALS8. (A)** By RT-qPCR, IL-1β-induced expression changes in Cluster 15 members (*SLC9A3R1, LGALS3*) are shown with respect to vehicle control (VC); these effects were reversed by both I-BET151 and I-BET762 (n=3/grp). **(B)** By global transcriptomic microarray analysis, a heatmap (left) displays average gene expression (n=3/grp) across 524 genes that were found to have significant expression alterations with respect to IL-1β vs. vehicle control (VC) and that were reversed by I-BET762 (IL-1β+BRD2889) as compared to IL-1β with vehicle control (IL-1β+VC). Gene set enrichment analysis (GSEA) of these differentially expressed genes revealed enrichment of pathways relevant to cell death, cell metabolism, and endothelial function. The heatmap (right) depicts gene membership in these Gene Ontology (GO) biologic processes of interest. **(C)** Representative scatter plot of MitoSOX Red staining followed by flow cytometry data (summarized in **Figure 2C**) showed IL-1β-driven upregulation of mitochondrial superoxide ($O_2^-$) levels reversed by I-BET762 (n=5/grp). **(D)** RT-qPCR analysis of C15 genes showed that the IL-1β-induced increase of *CD47* and decrease of *ABCC4* and *DAG1* were all rescued by I-BET762 (n=3/grp). The two isoforms of *LGALS8*, *LGALS8-L* and *LGALS8-M*, were increased and decreased by IL-1β, respectively; I-BET762 reversed the alterations of *LGALS8-L* expression but not *LGALS8-M* (n=3/grp). **(E)** By RT-qPCR, the expression of cluster 15 (C15) genes in IL-1β-exposed pulmonary artery smooth muscle cells (PASMCs) vs. vehicle control (VC) was assessed (n=3/grp). **(F)** RT-qPCR demonstrated siRNA specific to BRD2 (siBRD2) led to a ~40% reduction in *BRD2* transcript with no effect on *BRD4*, while siRNA specific to BRD4 (siBRD4) led to a ~90% reduction in *BRD4* transcript with no effect on *BRD2* and *LGALS8-L* was reduced by both siRNA alone and together – all compared to scrambled control (Scr; n=3/grp). **(G-H)** The IL-1β-induced expression of LGALS8-L was reversed by both Brd2 and 4 siRNA as confirmed by immunoblot at the protein level (n=3/grp). The data, except in (**B**) are plotted as mean ± SEM. Statistical

significance is indicated using one-way ANOVA with Bonferroni's multiple comparisons testing (*p<0.05, **<0.01, ***<0.001). See also **Tables S3-S4**.

**Figure S2. LGALS8 is upregulated in multiple animal and human examples of PH. (A)** By ELISA, the plasma level of LGALS8 expression was found to be unchanged among patients with Group 1 and 3 PH compare to non-PH controls (n=3-20/grp). **(B-E)** Similarly, via immunofluorescence staining (**B, D**), expression of LGALS8 was upregulated in whole-vessel in CD31+ cells (**C, E**) in lung sections from a SU5416-hypoxia (Su-Hyp) rat model of PH (n=5; **B**-**C**) and monocrotaline (MCT)-induced rat model of PH (n=4; **D-E**) as compared to control (n=4). **(F-I)** The increase of LGALS8 was also confirmed by immunoblot and quantification in whole lung homogenate from SU5416-hypoxia (Su-Hyp; n=4-5/grp; **F,H**) and monocrotaline (MCT; n=4-5/grp; **G,I**) rat models of PH. **(J–K)** In a chronic hypoxia mouse model of PH vs. control (n=6/grp), immunofluorescence staining (**J**) demonstrated increased LGALS8 in lung CD31+ endothelial cells (**K**). **(L-S)** Using immunofluorescence staining **(L)** and quantification, IL-1β expression was increased in lung CD31+ endothelial cells (**M**) of human patients with Group 1 and Group 3 PH compared to controls with non-PH (n=6-8/grp), rats with SU5416-hypoxia (Su-Hyp) (n=4-5/grp; **N-O**), rats with monocrotaline (MCT)-induced PH (n=4/grp; **P-Q**), and mice with hypoxia-induced PH (n=6/grp; **R-S**). Data are plotted as mean ± SEM. Statistical significance is indicated using one-way ANOVA with Bonferroni's multiple comparisons testing for (**A,M**) and Student's t-test for (**C-K**) and (**N-S**) (*p<0.05, **p<0.01, ***<0.001). Scale bar, 50 µm. See also **Table S5**.

**Figure S3. LGALS8 regulates endothelial apoptosis and function. (A-B)** Densitometry of immunoblots for integrin α3 (ITGA3) (**A**) and integrin β1 (ITGB1) (**B**) revealed increased expression of ITGA3 with IL-1β and no difference in expression of ITGB1, with 90% knockdown efficiency by their respective siRNAs. **(C)** siRNA knockdown of *LGALS8* as determined by RT-qPCR (n=3/grp) showed 90% efficiency. **(D)** Representative scatter plot of MitoSOX Red staining followed by flow cytometry demonstrated that IL-1β-dependent upregulation of mitochondrial

superoxide ($O_2^-$) was reversed by siRNA knockdown of *LGALS8* (siLGALS8; n=5/grp); data summarized in **Figure 3I**. **(E)** Representative scatter plot after flow cytometry showed that I-BET762-dependent reduction of IL-1β-induced mitochondrial superoxide ($O_2^-$) was attenuated by the presence of exogenous recombinant galectin-8 (rhGal8); data summarized in **Figure 3K** (n=3/grp). Statistical significance is indicated using one-way ANOVA with Bonferroni's multiple comparisons testing (*p<0.05, **p<0.01, ***p<0.001).

**Figure S4. Parameters of cardiovascular function in PAH rat models administered I-BET762. (A)** Heart rate of SU5416-hypoxic PAH rats administrated I-BET762 was not altered, as compared with vehicle control (VC) (n=4-5/grp). **(B-D)** SU5416-hypoxic rats administered I-BET762 did not exhibit altered left ventricular function compared to VC, as measured by left ventricle posterior wall (LVPW) thickness (**B**), ejection fraction (LVEF; **C**), and fractional shortening (LVFS; **D**) via transthoracic echocardiography (n=3-5/grp). (**E**) Aortic blood pressure (mean arterial pressure, MAP) of SU5416-hypoxic PAH rats administered I-BET762 was not altered, as compared with vehicle control (VC) (n=4-5/grp). **(F)** Heart rate of monocrotaline-exposed PAH rats administered I-BET762 was not altered, as compared with vehicle control (VC) (n=4-5/grp). (**G**) Aortic blood pressure (mean arterial pressure, MAP) of monocrotaline-exposed PAH rats administered I-BET762 was not altered, as compared with vehicle control (VC) (n=3-5/grp). The data are plotted as mean ± SEM. Statistical significance is indicated using Student's t-test (p>0.05 for all comparisons).

**Figure S5. IBET-762 and LGALS8 control oxidant and apoptotic endothelial pathways driven by hypoxia. (A)** In PAECs +/- hypoxic exposure, I-BET vs. vehicle controls (VC) reversed the hypoxia-induced increases of mitochondrial superoxide ($O_2^-$) levels as determined by flow cytometery of MitoSOX Red staining (n=5/grp). **(B)** In PAECs +/- hypoxic exposure, siLGALS8 compared with siRNA control (Scr) reversed the hypoxia-induced increases of mitochondrial superoxide ($O_2^-$) (n=5/grp). **(C-D)** Representative scatter plots of MitoSOX Red staining followed

by flow cytometry for experiments in (**A-B**). **(E)** In PAECs +/- hypoxic exposure, I-BET vs. VC (left graph) and siLGALS8 vs. Scr (right graph) reversed the hypoxia-induced increases of apoptosis, as assessed by caspase-3/7 activity (n=4-6/grp). The data are plotted as mean ± SEM. Statistical significance is indicated using two-way ANOVA with Bonferroni's multiple comparisons (*p<0.05, **<0.01, ***<0.001, ****<0.0001).

**Figure S6. Parameters of cardiovascular and pulmonary vascular expression and function in hypoxia-induced PH mice administered I-BET762 and in hypoxic *Lgals8-/-* mice. (A)** Heart rate of hypoxic mice administered I-BET762 was not altered compared with vehicle control (VC) (n=4-6/grp). **(B-D)** The knockout efficiency of *Lgals8*-/- mice was determined at the mRNA (**B**) and protein (**C-D**) level in whole lung lysate measured by RT-qPCR (n=6/grp) and immunoblot/densitometry (n=3/grp), respectively. **(E-G)** *Lgals8*-/- mice did not exhibit altered left ventricular function compared to their littermate controls (WT) as measured by left ventricle thickness (**E**), ejection fraction (LVEF; **E**), and fractional shortening (LVFS; **G**) via transthoracic echocardiography (n=7/grp). **(H)** Heart rate was also unchanged between *Lgals8-/-* mice and their littermate controls (WT) (n=6/grp). **(I-L)** To determine the effect of Lgals8 knockout on PH, *Lgals8*-/- mice were exposed to chronic hypoxia for 3 weeks. By immunofluorescence staining, IL-1β expression in the pulmonary vessels was not significantly altered in *Lgals8*-/- mice compared to WT mice (**I-J**, n=5/grp). Decreased apoptosis in whole lung homogenate was observed in *Lgals8-/-* mice as determined by immunoblot (**K**) and respective densitometry (**L**) of cleaved caspase 3 (n=3/grp).The data are plotted as mean ± SEM. Statistical significance is indicated using one-way ANOVA with Bonferroni's multiple comparisons testing for (**A**) and Student's t-test for (**B-L**) (*p<0.05, ***p≤0.001). Scale bar, 50 µm.

**Figure S7. BRD2889 controls C43 gene expression and improves mitochondrial function by targeting GSTP1.** (**A**) By RT-qPCR, in PAECs, expression of C43 genes *ISCU* and *mTOR* were decreased by hypoxia (48 hrs) and rescued by BRD2889 (5uM) vs. vehicle control (VC) (left

graph). Other C43 genes were either unaffected by hypoxia (*RECK*, *GOLGA*, and *RBL2*, middle graph) or unaffected in hypoxia by BRD2889 (*MID2, BANP, AGTRAP*, right graph); Un, non-vehicle exposed cells (n=4/grp). Of note, remaining C43 genes *KRT40* and *MT1G* were not assessed, given their disconnection from the BRD2889-specific DDN (**Fig. 5A**). **(B,C)** In PASMCs, by RT-qPCR, hypoxic expression of C43 genes *ISCU and mTOR* was unaffected by BRD2889 (5uM) (n=5/grp). **(D)** In PAECs, siRNA efficiency of GSTP1 was confirmed by RT-qPCR (n=4/grp). **(E)** In PAECs subjected to GSTP1 siRNA knockdown vs. scrambled siRNA control (Scr), a tracing was generated of oxygen consumption rate (OCR) using a Seahorse XFe96 flux analyzer recorded at baseline and following treatment with 1mM oligomycin, 0.5mM FCCP, and a 1mM rotenone and antimycin mixture (n=3/grp). **(F)** GSTP1 knockdown vs. Scr control resulted in an increase in basal, ATP-linked, and maximal respiration compared to Scr (n=3/grp). **(G-L)** After lentiviral forced expression of GSTP1 (LVGSTP1), GSTP1 transcript (by RT-qPCR, **G**, n=3/grp) and protein (by immunoblot, **H** and densitometry quantification, **I**, n=3/grp) were increased compared with vector control (LVCon). Forced GSTP1 expression downregulated ISCU protein (**J**, n=3/grp), increased apoptosis (by caspase 3,7 activity, **K**, n=4/grp), and decreased proliferation (by BrdU incorporation, **L**, n=4/grp). **(M-N)** Forced GSTP1 expression also decreased basal and mitochondrial OCR (n=3/grp). (**O-Q**) In hypoxic PAECs treated with BRD2889, siRNA knockdown of ISCU vs. Scr control siRNA was performed in normoxia or hypoxia; siRNA efficiency was confirmed by RT-qPCR (**O**) and immunoblot/densitometry (**P,Q**) (n=3/grp). These data are plotted as mean ± SEM. Statistical significance is indicated for multiple comparisons using one-way ANOVA with Bonferroni's multiple comparisons testing and for binary comparisons using Student's t-test (*p<0.05, **<0.01, ***<0.001, ****<0.0001).

**Figure S8. BRD2889 protects against endothelial-specific dysfunction induced by IL-6/soluble IL-6R+hypoxia. (A-C)** In hypoxic PAECs, proteasome inhibitor MG132 (5μM, 2h), BRD2889 (1μM, 24h), vehicle (VC), or no treatment (Un) were added, followed by (**A**) immunoblot

and densitometry of protein levels of ISCU (**B**) and GSH (glutathionylated-ISCU; **C**) (n=3/grp). MG132 reversed the hypoxia-induced reduction of ISCU without affecting GSTP1 or glutathionylation. BRD2889 also reversed the hypoxia-induced reduction of ISCU, but this was accompanied by a reversal of the hypoxic induction of ISCU glutathionylation. **(D-F)** In PAECs, BRD2889 reversed the IL-6/R+hypoxia-mediated increase of GST activity (**D**) without altering GSTP1 expression (**E**) (n=3/grp). In doing so, BRD2889 reversed the IL-6/R+hypoxic decrease of ISCU, as determined by immunoblot (**E**) and respective densitometry (**F**). **(G)** In PAECs, expression of proinflammatory genes in response to IL-6/soluble IL-6 receptor (IL-6/R) and chronic hypoxia in cultured PAECs. Expression of EDN1, VCAM1, and ICAM1 transcripts were analyzed by RT-qPCR. IL-6/sIL-6R+hypoxia treatment induced these inflammatory gene transcripts, but BRD2889 normalized this upregulation (n=3/grp). **(H-J)** BRD2889 improved endothelial function by reversing the IL-6/R+hypoxia-mediated decrease in mitochondrial Complex I activity (**H**), the increase in apoptotic caspase 3/7 activity (**I**), and the decrease in BrdU incorporation as a measure of proliferation (**J**) (n=6/grp). **(K-M)** PASMCs were exposed similarly to IL-6/R+hypoxia. Representative immunoblot (**K**) and densitometry (**L**) demonstrated hypoxic IL-6/R+hypoxia-induced ISCU reduction that was not rescued by BRD2889 (1uM). In addition, BRD2889 did not affect GSTP1 protein expression (**K**) or GST activity (**M**) (n=3/grp). **(N-P)** In PASMCs treated as in (**K**), BRD2889 did not affect the IL-6/sIL-6R+hypoxia-induced alterations of mitochondrial Complex I activity (**N**) and proliferation (**P**); and the modest alterations of apoptosis were only subtly changed by BRD2889 (**O**) (n=3/grp). **(Q-S)** siRNA knockdown of GSTP1 (siGSTP1) vs scrambled siRNA control (Scr) in PASMCs under normoxia or hypoxia. Despite knockdown of GSTP1 expression (by immunoblot and densitometry, **Q-R**), GST activity was not altered by siGSTP1 (**S**) (n=3/grp). These data are plotted as mean ± SEM. Statistical significance is indicated using one-way ANOVA with Bonferroni's multiple comparisons testing (*p<0.05, **<0.01, ***<0.001, ****<0.0001).

**Figure S9. Expression of ISCU and GSTP1 in human PH and parameters of cardiovascular function in hypoxic IL-6 Tg mice administered BRD2889. (A-E)** Using immunofluorescence staining and respective quantification, expression of ISCU was decreased (**A**) and GSTP1 (**B**) was increased in CD31+ (**C-D**) endothelium of lung sections from human patients with Group 1 (n=8) and Group 3 (n=8) PH as compared to non-PH controls (n=6). In addition, GST activity was increased in lung tissue of both Group 1 and Group 3 patients compared with non-PH controls (n=4/grp) (**E**). **(F)** Lung GST activity was also increased in hypoxic IL-6 Tg PAH mice vs. normoxic wildtype (WT) mice (n=4/grp). **(G-J)** No significant differences were observed in fractional shortening (**G**), ejection fraction (**H**), posterior wall (PW) thickness (**I**), and heart rate (**J**) of hypoxic IL-6 Tg mice following 10 days of treatment with either vehicle (n=3/grp) vs. BRD2889 (10mg/kg, n=4/grp). Statistical significance is indicated using Student's t test comparing vehicle-treated and BRD2889 treatment groups in mice. Statistical significance is indicated using one-way ANOVA with Bonferroni's multiple comparisons testing for comparing human samples (*p<0.05, **<0.01, ***<0.001, ****<0.0001). See also **Table S5**.

**Figure S10. Parameters of cardiovascular function in PAH rat models administered BRD2889. (A)** Heart rate of SU5416-hypoxic PAH rats administered BRD2889 was not altered, as compared with vehicle control (VC) (n=3-6/grp). **(B-D)** SU5416-hypoxic rats administered BRD2889 did not exhibit altered left ventricular function compared to VC, as measured by left ventricle posterior wall (LVPW) thickness (**B**), ejection fraction (LVEF; **C**), and fractional shortening (LVFS; **D**) via transthoracic echocardiography (n=5-6/grp). (**E**) Aortic blood pressure (mean arterial pressure, MAP) of SU5416-hypoxic PAH rats administered BRD2889 was not altered, as compared with vehicle control (VC) (n=3-6/grp). **(F-I)** By RT-qPCR, transcript levels of injury markers of the liver (*Got*, **F**; *Gpt1*, **G**) or kidney (*Cst3*, **H**; *Lcn2*, **I**) were not altered by BRD2889 vs. VC in SU5416-hypoxic PAH rats (n=4-6/grp). **(J)** Heart rate of monocrotaline-exposed PAH rats administered BRD2889 was not altered, as compared with vehicle control (VC)

(n=4-6/grp). **(K)** Aortic blood pressure (mean arterial pressure, MAP) of monocrotaline-exposed PAH rats administered BRD2889 was not altered, as compared with vehicle control (VC) (n=4-5/grp). **(L-P)** By RT-qPCR, transcript levels of injury markers of the liver (*Got*, **L**; *Gpt1*, **M**), kidney (*Cst3*, **N**; *Lcn2*, **O**), or intestines (*Ldha*, **P**) were not altered by BRD2889 vs. VC in monocrotaline-exposed PAH rats (n=3-4/grp). The data are plotted as mean ± SEM. Statistical significance is indicated using Student's t-test ($p>0.05$ for all comparisons).

## SUPPLEMENTAL TABLES

**Table S1. Cluster information and scores.** Clusters were sorted according to their rewiring scores as defined in **Methods**. This score considered the average p-value (mean(-log10(p))); 2) number (or frequency) of small molecules linked to rewiring of a given cluster (freq_drug); and 3) frequency of mediator involvement (freq_mediator). Rankings of clusters by these criteria individually (rank.pval, rank.freq_drug, rank.freq_mediator) as well as overall (rank.sum = sum of the ranks, rank.overall = rank based on sum.ranks) are listed. Genes in each cluster are also provided. This table is provided as separate excel sheet due to limited space here.

**Table S2. Small molecule scores.** Drugs were sorted according to their rewiring scores as defined in **Methods**. This score considered the average p-value (mean(-log10(p)); avg_pval); 2) number (or frequency) of rewired clusters linked to a given drug (freq_cluster); and 3) frequency of mediator involvement (freq_mediator). Rankings of clusters by these criteria individually (rank.pval, rank.freq_drug, rank.freq_mediator) as well as overall (rank.sum = sum of the ranks, rank.overall = rank based on sum.ranks) are listed. This table is provided as separate excel sheet due to limited space here.

**Table S3. Differential expression of PAEC genes altered by IL-1β and reversed by I-BET.** Affymetrix probe IDs and gene names are listed. Comparing IL-1β treatment vs. untreated (Un) as well as comparing I-BET vs. vehicle treatment (in the presence of IL-1β), $\log_2$(fold change), unadjusted p value, and adjusted p values are listed (n=3/grp). This table is provided as separate excel sheet due to limited space here.

**Table S4. Pathway enrichment in biologic processes relevant to Cell Death, Cell Metabolism, or Endothelial Function that are reversed by I-BET.** Gene set enrichment analysis was performed across those IL-1β-specific genes that were reversed by I-BET (**Table S3**). Gene Ontology (GO) pathways ranked by p value are listed, with significant enrichment noted

at adjusted p value < 0.05. Pathways, unadjusted p values (pval), and adjusted p values (padj) are listed, along with the total size of GO pathways and leading edge genes for each pathway. This table is provided as separate excel sheet due to limited space here.

**Table S5. Clinical characteristics of WSPH Group 1 PAH and Group 3 PH patients used for *in situ* staining and plasma profiling.** CHD, congenital heart disease; COPD, chronic obstructive pulmonary disease; CTD, connective tissue disease; IPAH, idiopathic pulmonary arterial hypertension; IPF, idiopathic pulmonary fibrosis; PH, pulmonary hypertension.

| **Age** | **Gender** | **mPAP* (mmHg)** | **Diagnosis** | **Clinical description** |
|---|---|---|---|---|
| **PAH patients** | | | | |
| 34 | Female | 50 | IPAH | Cardiopulmonary arrest (autopsy) |
| 64 | Female | 55 | IPAH | Cardiopulmonary arrest (autopsy) |
| 68 | Female | 44 | Scleroderma (PAH) | Bilateral lung transplant |
| 12 | Male | 53 | BMPR2 mutation (Hereditary PAH) | Bilateral lung transplant |
| 16 | Male | 62 | IPAH | Bilateral lung transplant |
| 1 | Male | 50 | Trisomy 21 (PAH) | Lung resection |
| 19 | Male | 48 | IPAH | Lung resection |
| 42 | Female | 57 | Scleroderma (PAH | Bilateral lung transplant |
| **Group 3 PH patients** | | | | |
| 62 | Male | 28 | IPF (Group 3 PH) | Bilateral lung transplant |
| 58 | Male | 28 | IPF (Group 3 PH) | Bilateral lung transplant |
| 63 | Male | 27 | IPF (Group 3 PH) | Bilateral lung transplant |
| 50 | Male | 30 | IPF (Group 3 PH) | Bilateral lung transplant |

| | | | | |
|---|---|---|---|---|
| 61 | Male | 37 | IPF (Group 3 PH) | Bilateral lung transplant |
| 69 | Female | 29 | IPF (Group 3 PH) | Bilateral lung transplant |
| 72 | Male | 46 | IPF (Group 3 PH) | Rapid autopsy |
| 66 | Male | 34 | IPF (Group 3 PH) | Bilateral lung transplant |
| **PERIPHERAL PLASMA SAMPLES** | | | | |
| 46 | Female | | Control | |
| 61 | Male | | Control | |
| 59 | Female | | Control | |
| 50 | Female | | Control | |
| 65 | Female | | Control | |
| 34 | Female | | Control | |
| 49 | Female | | Control | |
| 31 | Male | | Control | |
| 52 | Female | | Control | |
| 35 | Male | | Control | |
| 43 | Male | | Control | |
| 31 | Female | | Control | |
| 51 | Female | | Control | |
| 61 | Female | | Control | |
| 54 | Female | | Control | |
| 58 | Female | 53 | IPAH | |
| 47 | Male | 52 | IPAH | |
| 43 | Female | 53 | CHD (PAH) | |
| 83 | Male | 45 | IPAH | |
| 53 | Female | 54 | IPAH | |

| | | | | |
|---|---|---|---|---|
| 47 | Female | 32 | CTD (PAH) | |
| 73 | Female | 28 | IPAH | |
| 55 | Female | 52 | CTD (PAH) | |
| 41 | Male | 51 | IPAH | |
| 73 | Female | 41 | Scleroderma (PAH) | |
| 65 | Female | 32 | Scleroderma (PAH) | |
| 57 | Male | 53 | IPAH | |
| 26 | Female | 34 | IPAH | |
| 58 | Male | 61 | IPAH | |
| 58 | Female | 53 | CTD (PAH) | |
| 73 | Female | 32 | Scleroderma (PAH) | |
| 37 | Male | 37 | Scleroderma (PAH) | |
| 72 | Female | 27 | Scleroderma (PAH) | |
| 67 | Male | 49 | Portopulmonary hypertension (PAH) | |
| 58 | Male | 43 | CTD (PAH) | |
| 68 | Female | 25.3 | COPD (Group 3 PH) | |
| 70 | Male | 37 | COPD (Group 3 PH) | |
| 40 | Female | 29 | COPD (Group 3 PH) | |

*Mean pulmonary arterial pressure (mPAP)

**Table S6. Differential expression of PAEC genes altered by hypoxia and reversed by BRD2889.** Gene symbols are listed. Comparing hypoxia vs. normoxia as well as comparing BRD2889 vs. vehicle treatment (in hypoxia), $\log_2$(fold change), unadjusted p value, and adjusted p values are listed (n=3/grp). This table is provided as separate excel sheet due to limited space here.

**Table S7. Pathway enrichment in biologic processes relevant to Cell Cycle, Cell Death, or Metabolism that are reversed by BRD2889.** Gene set enrichment analysis was performed across those IL-1β-specific genes that were reversed by I-BET (**Table S6**). Gene Ontology (GO) pathways ranked by p value are listed, with significant enrichment noted at adjusted p value < 0.05. Pathways, unadjusted p values (pval), and adjusted p values (padj) are listed, along with the total size of GO pathways and leading edge genes for each pathway. This table is provided as separate excel sheet due to limited space here.

**Table S8. Key Resources.**

| Reagent/resource | Source | Identifier |
|---|---|---|
| PAECs | Lonza | CC-2530 |
| PASMCs | Lonza | CC-2581 |
| Endothelial cell growth media | Lonza | CC-3121, CC-4133 |
| Smooth muscle cell growth media | Lonza | CC-3182 |
| Human recombinant IL-1β | Peprotech | 200-01B |
| DMSO | Sigma | 41639 |
| I-BET151 | Selleckchem | S2780 |
| I-BET762 | Selleckchem | S7189 |
| Human recombinant galectin-8 | R&D Systems | 1305-GA-050 |
| IL-6/IL-6R alpha Protein Chimera | Millipore | 8954-SR |
| BRD2889 | Broad Institute | (*22*) |
| MG132 | Sigma Aldrich | 474791 |
| C57BL/6J mice | Jackson Laboratory | RRID: IMSR_JAX:000664 |

| | | |
|---|---|---|
| *Lgals8*-/- mice | KOMP | RRID: MGI:5824822 |
| Sprague-Dawley rats | Charles River Laboratory | RRID: RGD_10395233 |
| IL-6 transgenic mice | | (*68*) |
| human LGALS8 | RnD Systems | Cat# AF1305, RRID: AB_2137229 |
| mouse/rat Lgals8 | Abcam | Cat# ab69631, RRID: AB_1268941 |
| pSTAT1 | Abcam | Cat# ab29045, RRID: AB_778096 |
| STAT1 | Abcam | Cat# ab47425, RRID: AB_882708 |
| GSTP1 | Abcam | Cat# ab153949, RRID: AB_2877700 |
| ISCU | Prointech | Cat#14812-1-AP, RRID: AB_2280362 |
| Glutathione | Abcam | Cat# ab19534, RRID: AB_880243 |
| HIF2A | Novous | Cat# NB100-122, RRID: AB_10002593 |
| α-Tubulin | Millipore sigma | Cat# CP06, RRID: AB_2617116 |
| ACTB | Santa Cruz | Cat# sc-47778, RRID: AB_2714189 |
| IL-1β | Abcam | Cat# ab9722, RRID: AB_308765 |
| cleaved caspase3 | Cell Signaling | Cat# cs-9661, RRID: AB_2341188 |
| α-SMA | Sigma | Cat# F3777, RRID: AB_476977 |
| CD31 | Abcam | Cat# ab7388, RRID: AB_305905 |
| Taqman primers | Thermo Fisher Scientific | *LGALS8* (Hs01057135_m1), *LGALS3* (Hs00173587_m1), *NHERF1* (Hs00188594_m1), *NHERF2* (Hs01033104_g1), *ABCC4* (Hs00988721_m1), *CD47* |

| | | |
|---|---|---|
| | | (Hs00179953_m1), *DAG1* (Hs00189308_m1), *LGALS8-L* (Hs01062767_g1), *VCAM1* (Hs01003372_m1), *VEGF* (Hs00900055_m1), *CD31* (Hs00169777_m1), *CDH5* (Hs00901465_m1), *BRD2* (Hs01121986_g1), *BRD4* (Hs04188087_m1), *STAT1* (Hs01013996_m1), *GSTP1* (Hs04419827_g1), *ISCU* (Hs00384510_m1), *RECK* (Hs00221638_m1), *MT1G* (Hs02578922_g1), *MTOR* (Hs00234508_m1), *GOLGA2* (Hs01067737_m1), *MID2* (Hs00201978_m1), *KRT40* (Hs01057909_m1), *BANP* (Hs00215370_m1), *AGTRAP* (Hs01564425_m1), *RBL2* (Hs00180562_m1) |

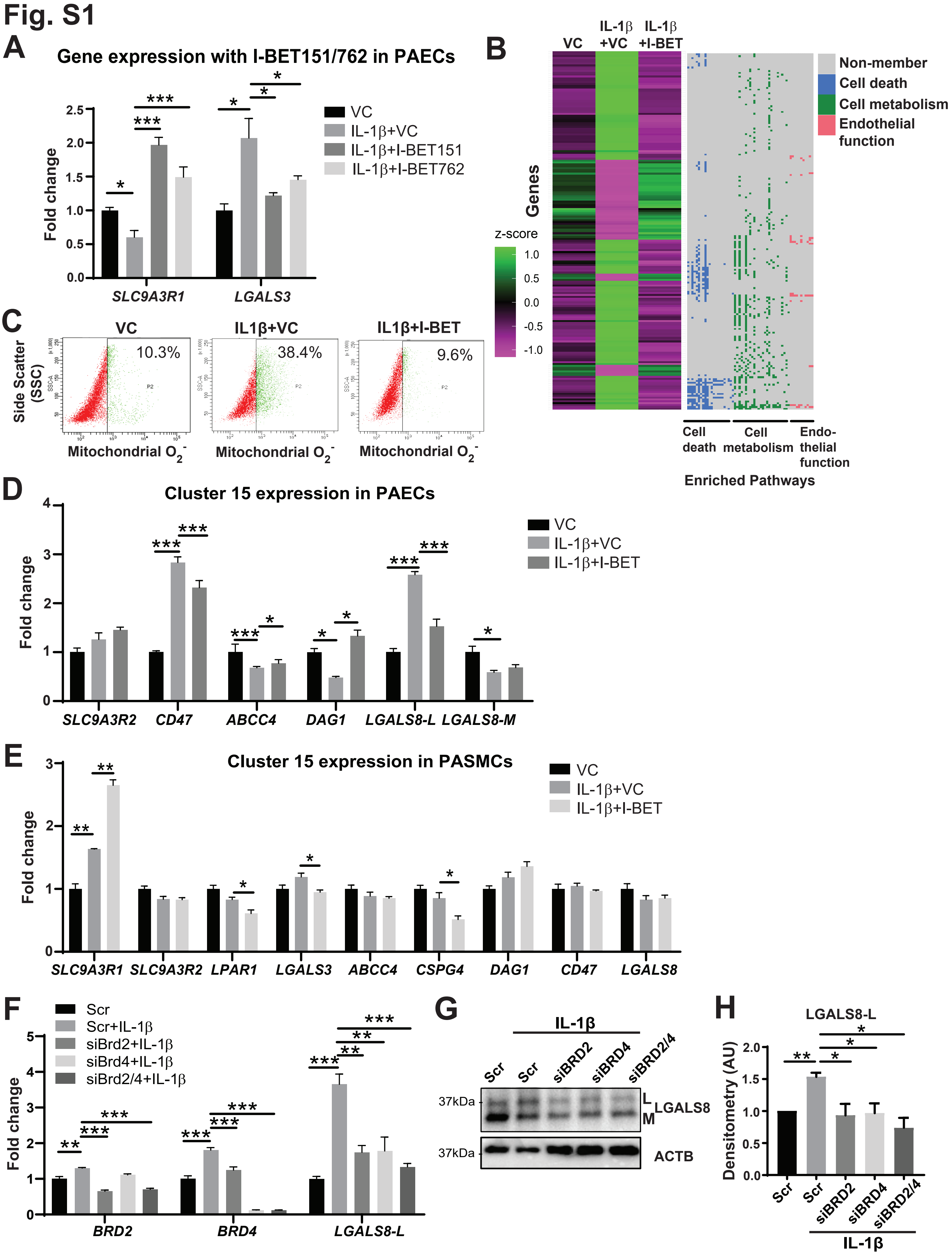
Fig. S1
A
Gene expression with I-BET151/762 in PAECs
Fold change
SLC9A3R1
LGALS3
VC
IL-1β+VC
IL-1β+I-BET151
IL-1β+I-BET762
B
VC
IL-1β +VC
IL-1β +I-BET
Genes
z-score
Non-member
Cell death
Cell metabolism
Endothelial function
Cell death
Cell metabolism
Endo-thelial function
Enriched Pathways
C
VC
IL1β+VC
IL1β+I-BET
10.3%
38.4%
9.6%
Side Scatter (SSC)
Mitochondrial O2-
D
Cluster 15 expression in PAECs
Fold change
SLC9A3R2
CD47
ABCC4
DAG1
LGALS8-L
LGALS8-M
VC
IL-1β+VC
IL-1β+I-BET
E
Cluster 15 expression in PASMCs
Fold change
SLC9A3R1
SLC9A3R2
LPAR1
LGALS3
ABCC4
CSPG4
DAG1
CD47
LGALS8
VC
IL-1β+VC
IL-1β+I-BET
F
Scr
Scr+IL-1β
siBrd2+IL-1β
siBrd4+IL-1β
siBrd2/4+IL-1β
Fold change
BRD2
BRD4
LGALS8-L
G
IL-1β
Scr
Scr
siBRD2
siBRD4
siBRD2/4
37kDa
L
M
LGALS8
37kDa
ACTB
H
LGALS8-L
Densitometry (AU)
Scr
Scr
siBRD2
siBRD4
siBRD2/4
IL-1β

Fig. S2

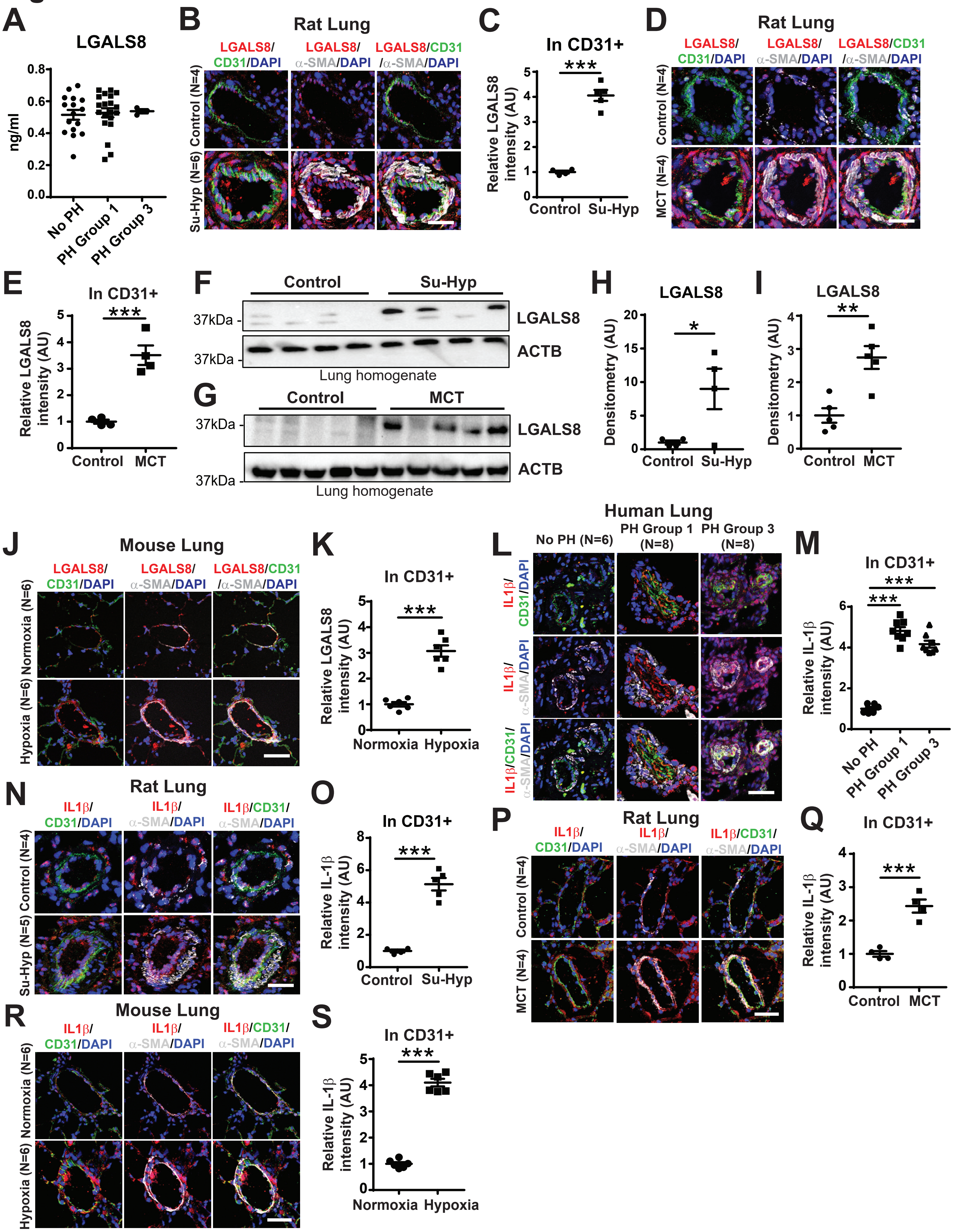
A
LGALS8
ng/ml
No PH
PH Group 1
PH Group 3
B
Rat Lung
LGALS8/CD31/DAPI
LGALS8/α-SMA/DAPI
LGALS8/CD31/α-SMA/DAPI
Control (N=4)
Su-Hyp (N=6)
C
In CD31+
***
Relative LGALS8 intensity (AU)
Control
Su-Hyp
D
Rat Lung
Control (N=4)
MCT (N=4)
E
In CD31+
***
Relative LGALS8 intensity (AU)
Control
MCT
F
Control
Su-Hyp
37kDa
LGALS8
ACTB
Lung homogenate
G
Control
MCT
37kDa
LGALS8
ACTB
Lung homogenate
H
LGALS8
*
Densitometry (AU)
Control
Su-Hyp
I
LGALS8
**
Densitometry (AU)
Control
MCT
J
Mouse Lung
Normoxia (N=6)
Hypoxia (N=6)
K
In CD31+
***
Relative LGALS8 intensity (AU)
Normoxia
Hypoxia
L
Human Lung
No PH (N=6)
PH Group 1 (N=8)
PH Group 3 (N=8)
IL1β/CD31/DAPI
IL1β/α-SMA/DAPI
IL1β/CD31/α-SMA/DAPI
M
In CD31+
***
***
Relative IL-1β intensity (AU)
No PH
PH Group 1
PH Group 3
N
Rat Lung
IL1β/CD31/DAPI
IL1β/α-SMA/DAPI
IL1β/CD31/α-SMA/DAPI
Control (N=4)
Su-Hyp (N=5)
O
In CD31+
***
Relative IL-1β intensity (AU)
Control
Su-Hyp
P
Rat Lung
Control (N=4)
MCT (N=4)
Q
In CD31+
***
Relative IL-1β intensity (AU)
Control
MCT
R
Mouse Lung
Normoxia (N=6)
Hypoxia (N=6)
S
In CD31+
***
Relative IL-1β intensity (AU)
Normoxia
Hypoxia

## Fig. S3

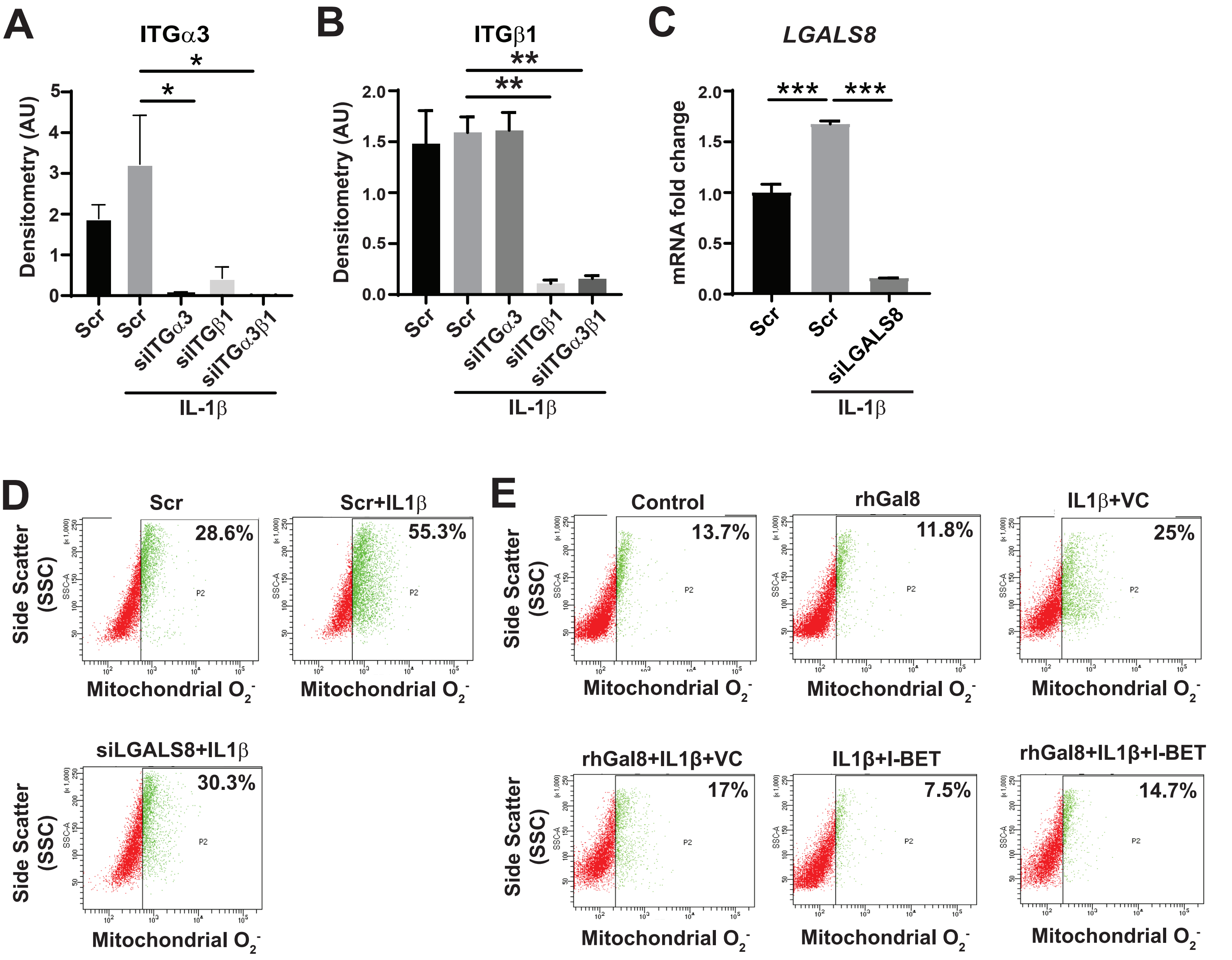
A
ITGα3
Densitometry (AU)
Scr
Scr
siITGα3
siITGβ1
siITGα3β1
IL-1β
B
ITGβ1
Densitometry (AU)
Scr
Scr
siITGα3
siITGβ1
siITGα3β1
IL-1β
C
LGALS8
mRNA fold change
Scr
Scr
siLGALS8
IL-1β
D
Scr
28.6%
Scr+IL1β
55.3%
siLGALS8+IL1β
30.3%
Side Scatter (SSC)
Mitochondrial O2-
E
Control
13.7%
rhGal8
11.8%
IL1β+VC
25%
rhGal8+IL1β+VC
17%
IL1β+I-BET
7.5%
rhGal8+IL1β+I-BET
14.7%
Side Scatter (SSC)
Mitochondrial O2-

Fig. S4

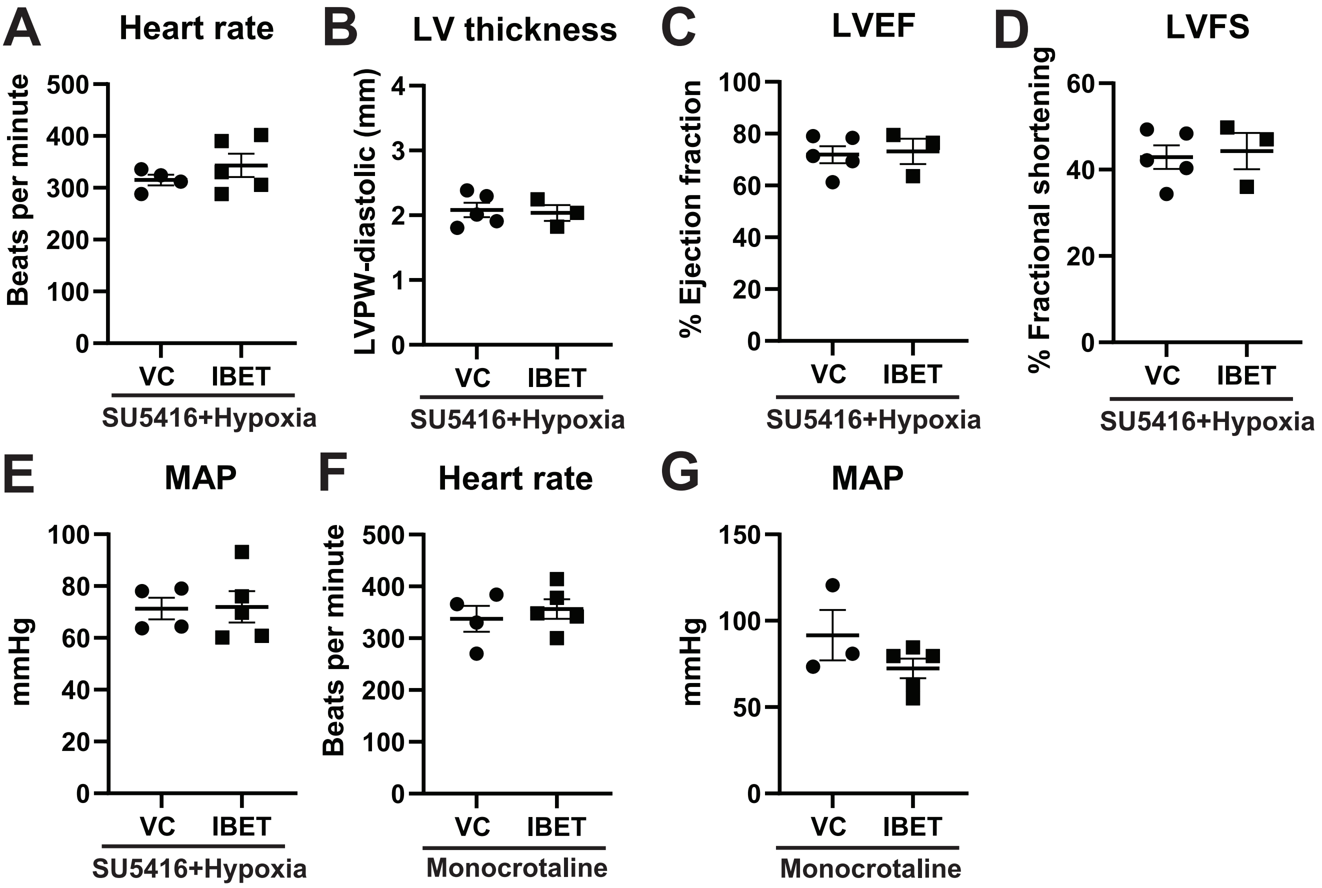
A
Heart rate
Beats per minute
500
400
300
200
100
0
VC
IBET
SU5416+Hypoxia
B
LV thickness
LVPW-diastolic (mm)
4
3
2
1
0
VC
IBET
SU5416+Hypoxia
C
LVEF
% Ejection fraction
100
80
60
40
20
0
VC
IBET
SU5416+Hypoxia
D
LVFS
% Fractional shortening
60
40
20
0
VC
IBET
SU5416+Hypoxia
E
MAP
mmHg
100
80
60
40
20
0
VC
IBET
SU5416+Hypoxia
F
Heart rate
Beats per minute
500
400
300
200
100
0
VC
IBET
Monocrotaline
G
MAP
mmHg
150
100
50
0
VC
IBET
Monocrotaline

# Fig. S5

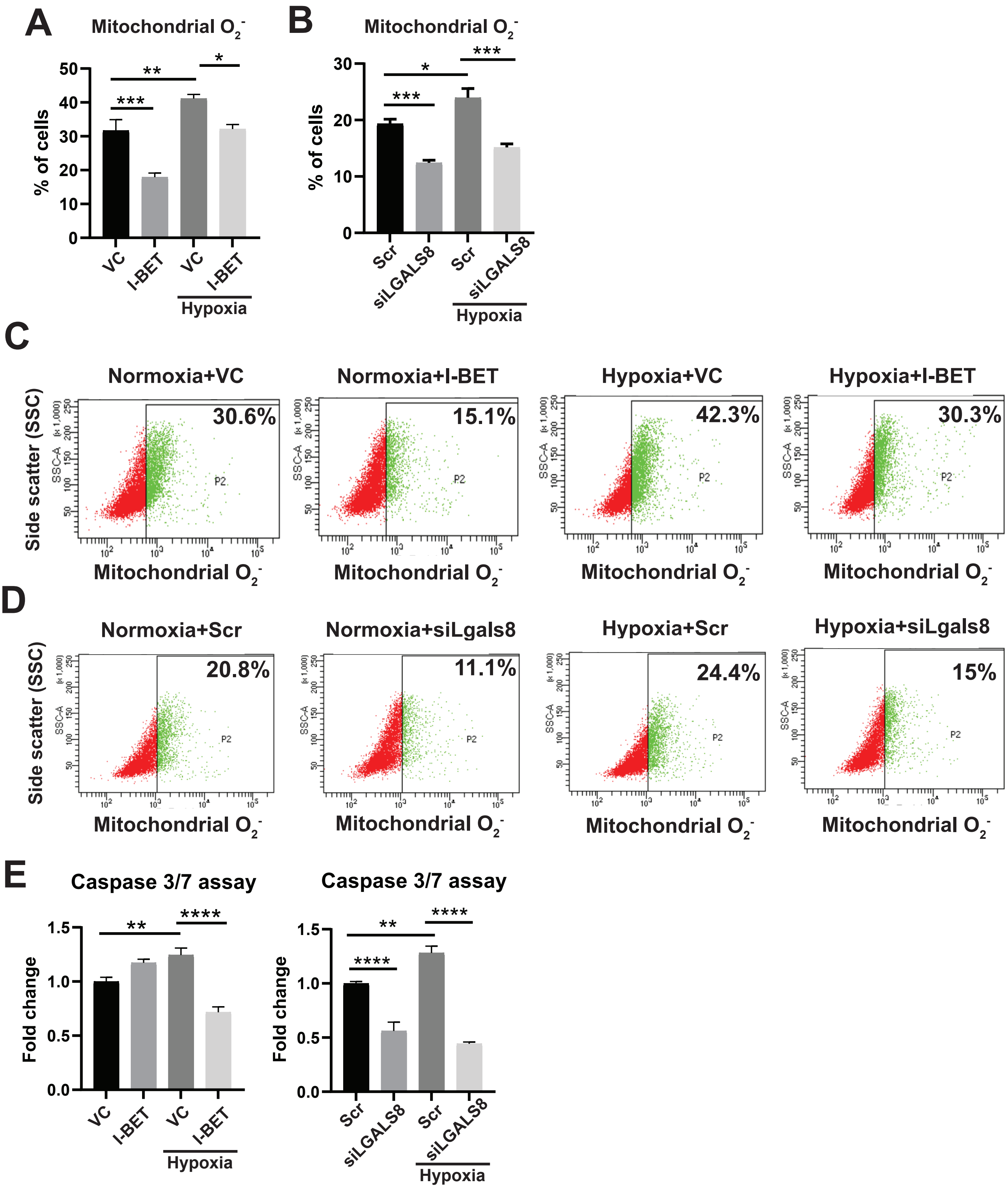

A
Mitochondrial $O_2^-$
% of cells
0
10
20
30
40
50
**
***
*
VC
I-BET
VC
I-BET
Hypoxia
B
Mitochondrial $O_2^-$
% of cells
0
10
20
30
*
***
***
Scr
siLGALS8
Scr
siLGALS8
Hypoxia
C
Normoxia+VC
Normoxia+I-BET
Hypoxia+VC
Hypoxia+I-BET
Side scatter (SSC)
30.6%
15.1%
42.3%
30.3%
P2
Mitochondrial $O_2^-$
D
Normoxia+Scr
Normoxia+siLgals8
Hypoxia+Scr
Hypoxia+siLgals8
Side scatter (SSC)
20.8%
11.1%
24.4%
15%
P2
Mitochondrial $O_2^-$
E
Caspase 3/7 assay
Fold change
0.0
0.5
1.0
1.5
**
****
VC
I-BET
VC
I-BET
Hypoxia
Caspase 3/7 assay
Fold change
0.0
0.5
1.0
1.5
**
****
****
Scr
siLGALS8
Scr
siLGALS8
Hypoxia

Fig. S6

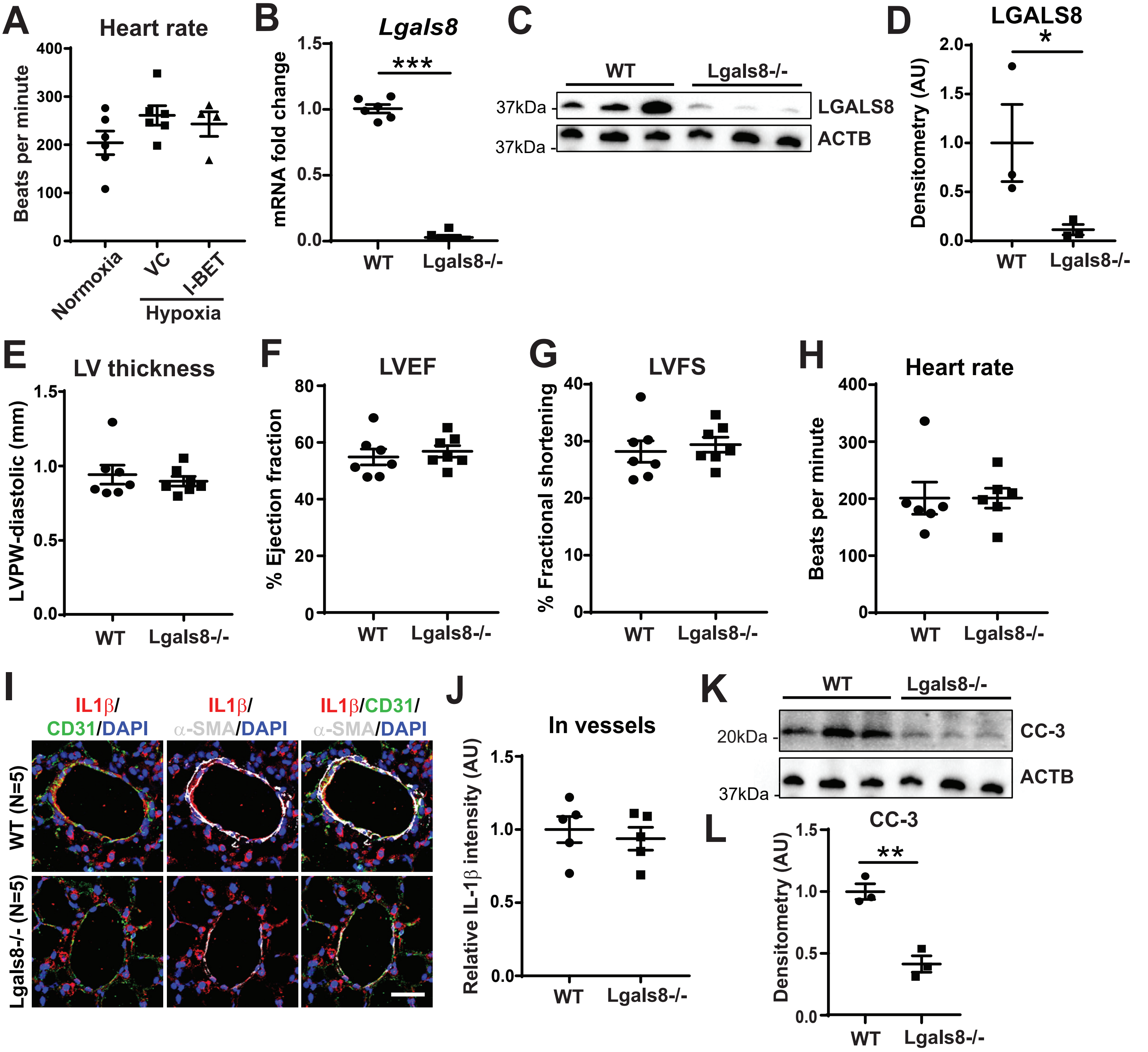

A
Heart rate
Beats per minute
400
300
200
100
0
Normoxia
VC
I-BET
Hypoxia
B
Lgals8
mRNA fold change
1.5
1.0
0.5
0.0
***
WT
Lgals8-/-
C
WT
Lgals8-/-
37kDa
LGALS8
37kDa
ACTB
D
LGALS8
*
Densitometry (AU)
2.0
1.5
1.0
0.5
0.0
WT
Lgals8-/-
E
LV thickness
LVPW-diastolic (mm)
1.5
1.0
0.5
0.0
WT
Lgals8-/-
F
LVEF
% Ejection fraction
80
60
40
20
0
WT
Lgals8-/-
G
LVFS
% Fractional shortening
40
30
20
10
0
WT
Lgals8-/-
H
Heart rate
Beats per minute
400
300
200
100
0
WT
Lgals8-/-
I
IL1β/CD31/DAPI
IL1β/α-SMA/DAPI
IL1β/CD31/α-SMA/DAPI
WT (N=5)
Lgals8-/- (N=5)
J
In vessels
Relative IL-1β intensity (AU)
1.5
1.0
0.5
0.0
WT
Lgals8-/-
K
WT
Lgals8-/-
20kDa
CC-3
37kDa
ACTB
L
CC-3
**
Densitometry (AU)
1.5
1.0
0.5
0.0
WT
Lgals8-/-

# Fig. S7

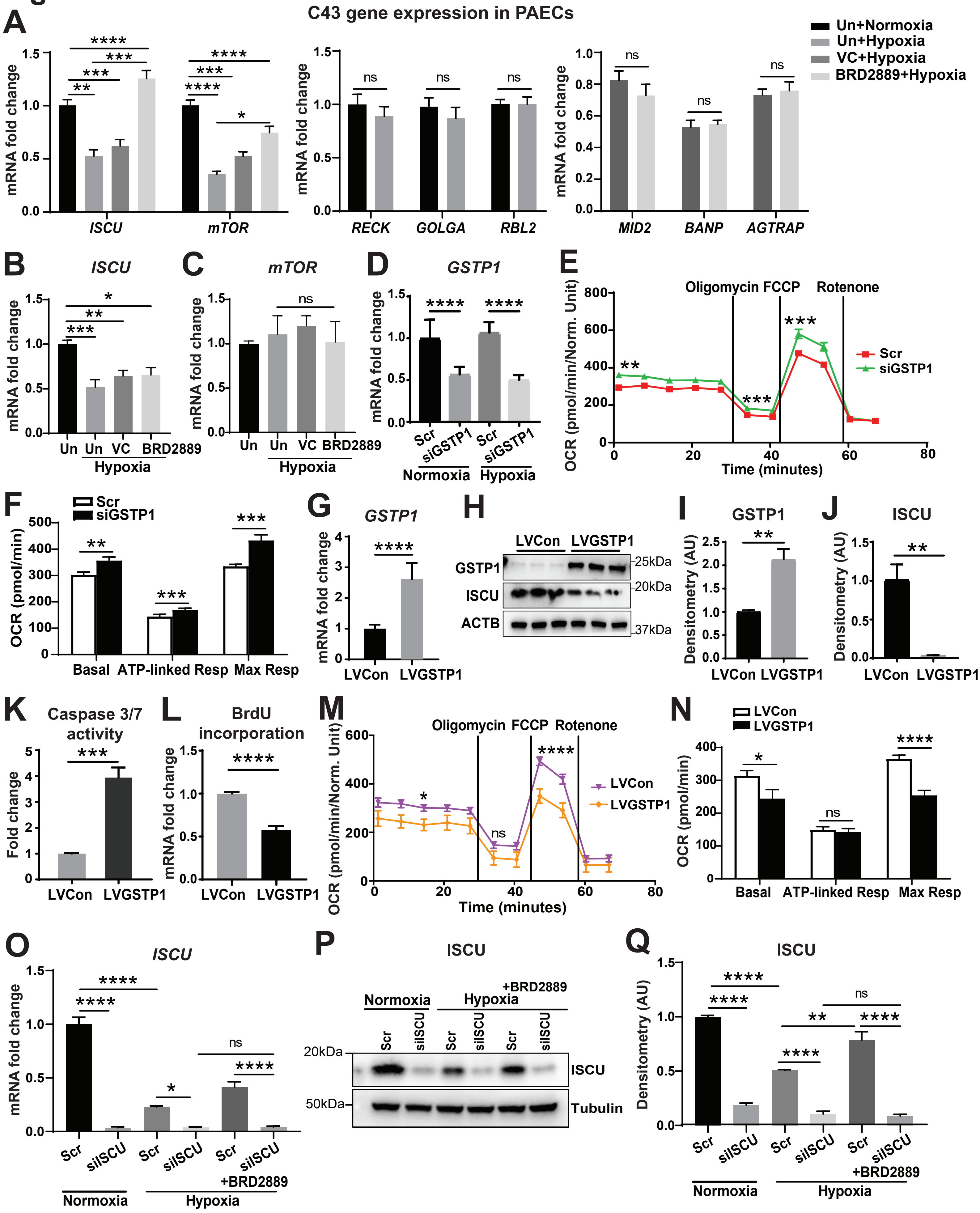

A
C43 gene expression in PAECs
Un+Normoxia
Un+Hypoxia
VC+Hypoxia
BRD2889+Hypoxia
mRNA fold change
ISCU
mTOR
RECK
GOLGA
RBL2
MID2
BANP
AGTRAP
ns
B
ISCU
Un
VC
BRD2889
Hypoxia
C
mTOR
D
GSTP1
Scr
siGSTP1
Normoxia
Hypoxia
E
Oligomycin FCCP
Rotenone
OCR (pmol/min/Norm. Unit)
Time (minutes)
F
OCR (pmol/min)
Basal
ATP-linked Resp
Max Resp
G
LVCon
LVGSTP1
H
GSTP1
ACTB
25kDa
20kDa
37kDa
I
Densitometry (AU)
J
K
Caspase 3/7 activity
Fold change
L
BrdU incorporation
M
Oligomycin FCCP Rotenone
N
O
siISCU
+BRD2889
P
20kDa
50kDa
Tubulin
Q

# Fig. S8

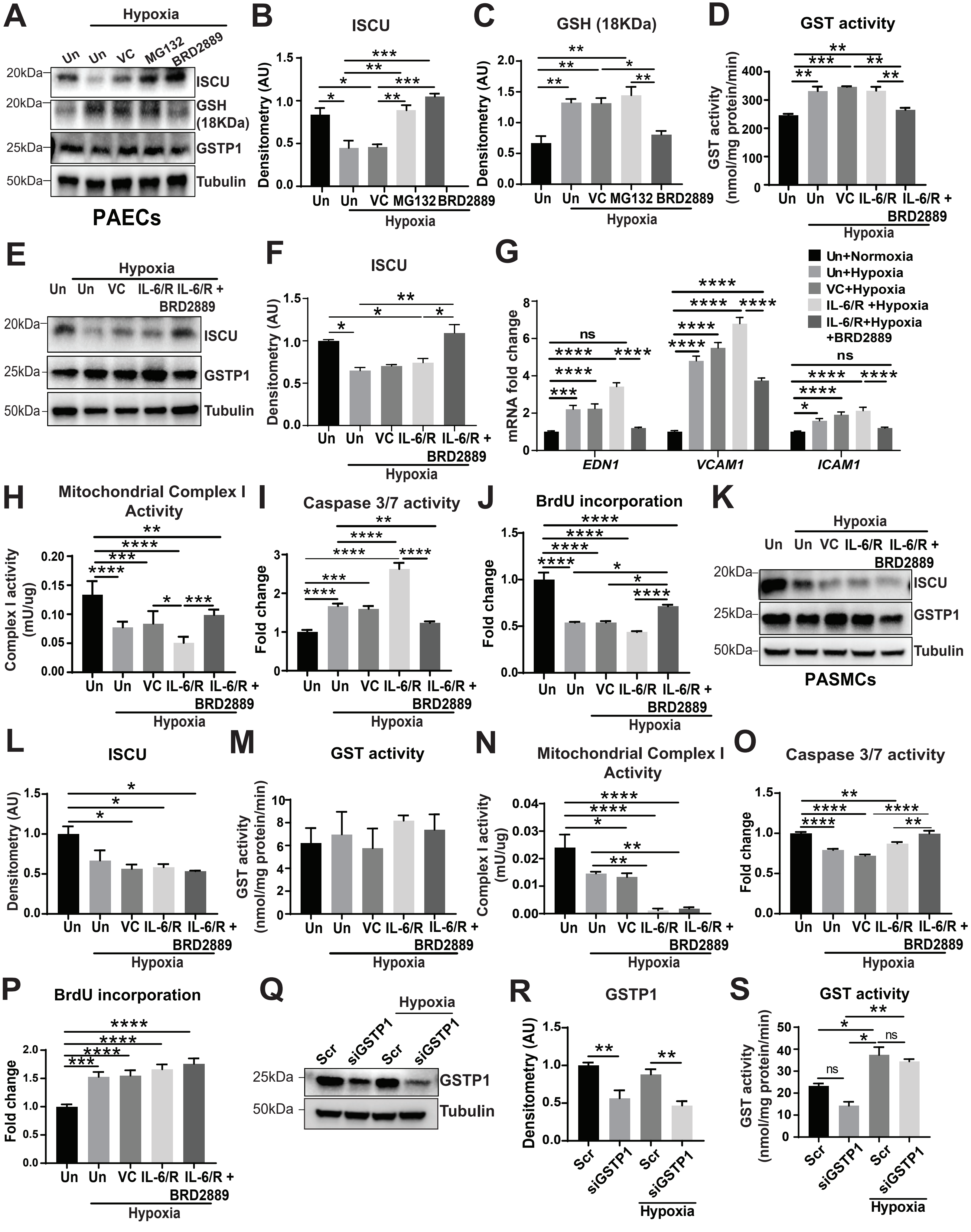

A
Hypoxia
Un Un VC MG132 BRD2889
20kDa ISCU
20kDa GSH (18KDa)
25kDa GSTP1
50kDa Tubulin
PAECs
B
ISCU
Densitometry (AU)
Un Un VC MG132 BRD2889
Hypoxia
C
GSH (18KDa)
Densitometry (AU)
Un Un VC MG132 BRD2889
Hypoxia
D
GST activity
GST activity (nmol/mg protein/min)
Un Un VC IL-6/R IL-6/R + BRD2889
Hypoxia
E
Hypoxia
Un Un VC IL-6/R IL-6/R + BRD2889
20kDa ISCU
25kDa GSTP1
50kDa Tubulin
F
ISCU
Densitometry (AU)
Un Un VC IL-6/R IL-6/R + BRD2889
Hypoxia
G
Un+Normoxia
Un+Hypoxia
VC+Hypoxia
IL-6/R +Hypoxia
IL-6/R+Hypoxia +BRD2889
mRNA fold change
EDN1 VCAM1 ICAM1
H
Mitochondrial Complex I Activity
Complex I activity (mU/ug)
Un Un VC IL-6/R IL-6/R + BRD2889
Hypoxia
I
Caspase 3/7 activity
Fold change
Un Un VC IL-6/R IL-6/R + BRD2889
Hypoxia
J
BrdU incorporation
Fold change
Un Un VC IL-6/R IL-6/R + BRD2889
Hypoxia
K
Hypoxia
Un Un VC IL-6/R IL-6/R + BRD2889
20kDa ISCU
25kDa GSTP1
50kDa Tubulin
PASMCs
L
ISCU
Densitometry (AU)
Un Un VC IL-6/R IL-6/R + BRD2889
Hypoxia
M
GST activity
GST activity (nmol/mg protein/min)
Un Un VC IL-6/R IL-6/R + BRD2889
Hypoxia
N
Mitochondrial Complex I Activity
Complex I activity (mU/ug)
Un Un VC IL-6/R IL-6/R + BRD2889
Hypoxia
O
Caspase 3/7 activity
Fold change
Un Un VC IL-6/R IL-6/R + BRD2889
Hypoxia
P
BrdU incorporation
Fold change
Un Un VC IL-6/R IL-6/R + BRD2889
Hypoxia
Q
Hypoxia
Scr siGSTP1 Scr siGSTP1
25kDa GSTP1
50kDa Tubulin
R
GSTP1
Densitometry (AU)
Scr siGSTP1 Scr siGSTP1
Hypoxia
S
GST activity
GST activity (nmol/mg protein/min)
Scr siGSTP1 Scr siGSTP1
Hypoxia

Fig. S9

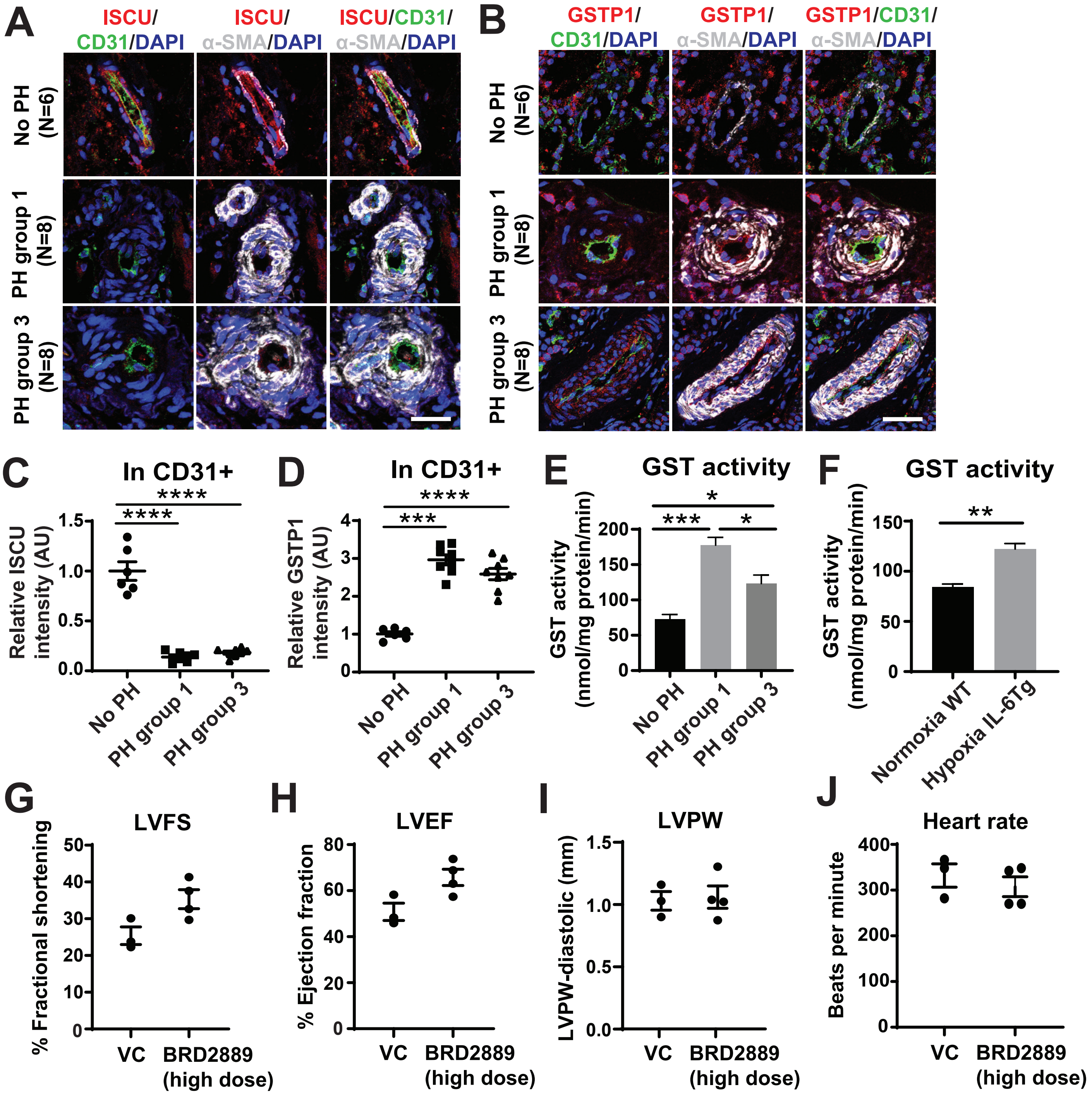

A
ISCU/CD31/DAPI
ISCU/α-SMA/DAPI
ISCU/CD31/α-SMA/DAPI
No PH (N=6)
PH group 1 (N=8)
PH group 3 (N=8)
B
GSTP1/CD31/DAPI
GSTP1/α-SMA/DAPI
GSTP1/CD31/α-SMA/DAPI
No PH (N=6)
PH group 1 (N=8)
PH group 3 (N=8)
C
In CD31+
Relative ISCU intensity (AU)
1.5
1.0
0.5
0.0
****
****
No PH
PH group 1
PH group 3
D
In CD31+
Relative GSTP1 intensity (AU)
4
3
2
1
0
***
****
No PH
PH group 1
PH group 3
E
GST activity
GST activity (nmol/mg protein/min)
200
150
100
50
0
*
***
*
No PH
PH group 1
PH group 3
F
GST activity
GST activity (nmol/mg protein/min)
150
100
50
0
**
Normoxia WT
Hypoxia IL-6Tg
G
LVFS
% Fractional shortening
50
40
30
20
10
0
VC
BRD2889 (high dose)
H
LVEF
% Ejection fraction
80
60
40
20
0
VC
BRD2889 (high dose)
I
LVPW
LVPW-diastolic (mm)
1.5
1.0
0.5
0.0
VC
BRD2889 (high dose)
J
Heart rate
Beats per minute
400
300
200
100
0
VC
BRD2889 (high dose)

Fig. S10

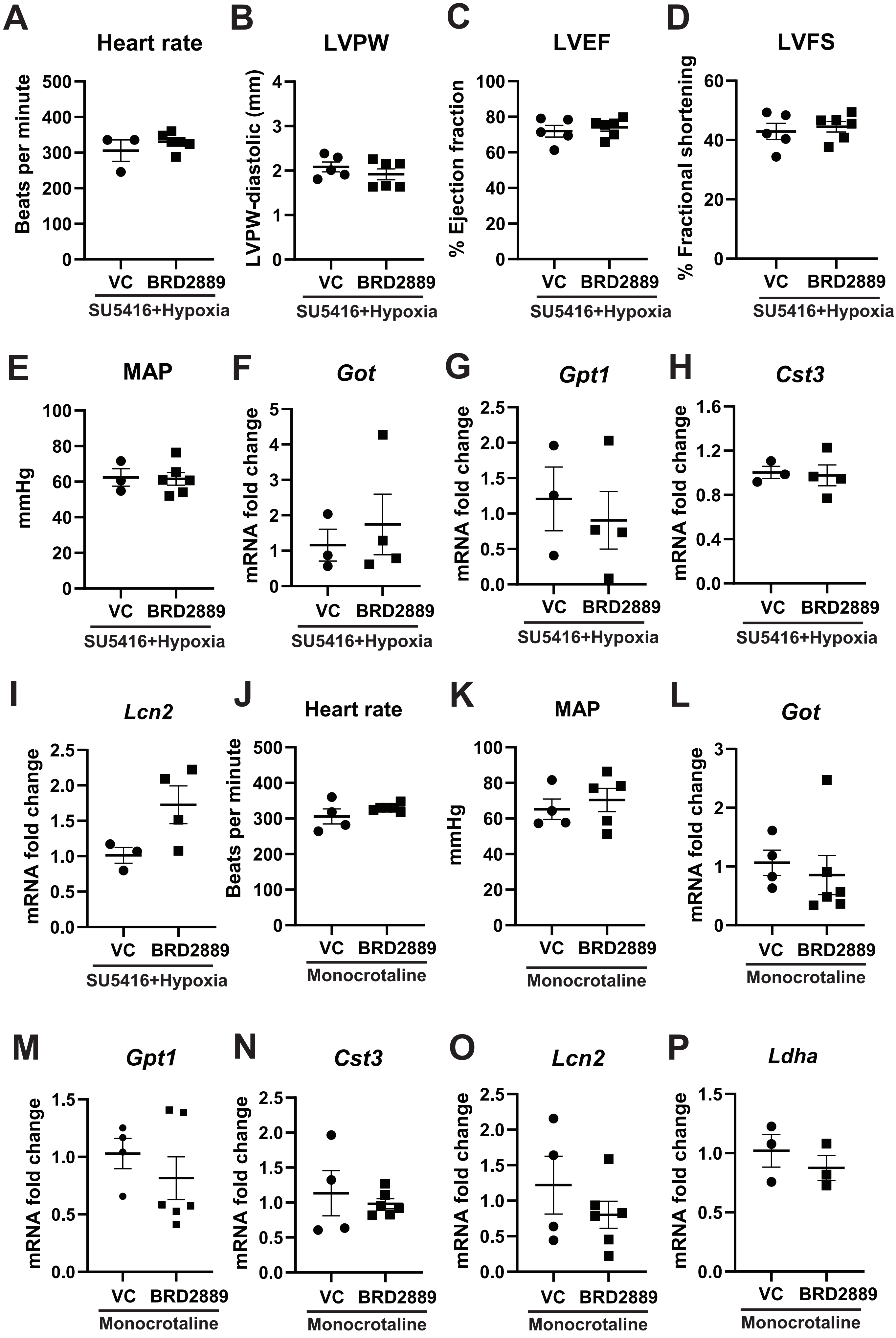
A
Heart rate
Beats per minute
500
400
300
200
100
0
VC
BRD2889
SU5416+Hypoxia
B
LVPW
LVPW-diastolic (mm)
4
3
2
1
0
VC
BRD2889
SU5416+Hypoxia
C
LVEF
% Ejection fraction
100
80
60
40
20
0
VC
BRD2889
SU5416+Hypoxia
D
LVFS
% Fractional shortening
60
40
20
0
VC
BRD2889
SU5416+Hypoxia
E
MAP
mmHg
100
80
60
40
20
0
VC
BRD2889
SU5416+Hypoxia
F
Got
mRNA fold change
5
4
3
2
1
0
VC
BRD2889
SU5416+Hypoxia
G
Gpt1
mRNA fold change
2.5
2.0
1.5
1.0
0.5
0.0
VC
BRD2889
SU5416+Hypoxia
H
Cst3
mRNA fold change
1.6
1.2
0.8
0.4
0.0
VC
BRD2889
SU5416+Hypoxia
I
Lcn2
mRNA fold change
2.5
2.0
1.5
1.0
0.5
0.0
VC
BRD2889
SU5416+Hypoxia
J
Heart rate
Beats per minute
500
400
300
200
100
0
VC
BRD2889
Monocrotaline
K
MAP
mmHg
100
80
60
40
20
0
VC
BRD2889
Monocrotaline
L
Got
mRNA fold change
3
2
1
0
VC
BRD2889
Monocrotaline
M
Gpt1
mRNA fold change
1.5
1.0
0.5
0.0
VC
BRD2889
Monocrotaline
N
Cst3
mRNA fold change
2.5
2.0
1.5
1.0
0.5
0.0
VC
BRD2889
Monocrotaline
O
Lcn2
mRNA fold change
2.5
2.0
1.5
1.0
0.5
0.0
VC
BRD2889
Monocrotaline
P
Ldha
mRNA fold change
1.5
1.0
0.5
0.0
VC
BRD2889
Monocrotaline

## REFERENCES AND NOTES


1. K. Park, A review of computational drug repurposing. *Transl. Clin. Pharmacol.* **27**, 59–63 (2019).

2. Q. Vanhaelen, P. Mamoshina, A. M. Aliper, A. Artemov, K. Lezhnina, I. Ozerov, I. Labat, A. Zhavoronkov, Design of efficient computational workflows for in silico drug repurposing. *Drug Discov. Today* **22**, 210–222 (2017).

3. G. Simonneau, D. Montani, D. S. Celermajer, C. P. Denton, M. A. Gatzoulis, M. Krowka, P. G. Williams, R. Souza, Haemodynamic definitions and updated clinical classification of pulmonary hypertension. *Eur. Respir. J.* **53**, 1801913 (2019).

4. C. E. Evans, N. D. Cober, Z. Dai, D. J. Stewart, Y.-Y. Zhao, Endothelial cells in the pathogenesis of pulmonary arterial hypertension. *Eur. Respir. J.*, 2003957 (2021).

5. E. D. Michelakis, Spatio-temporal diversity of apoptosis within the vascular wall in pulmonary arterial hypertension: Heterogeneous BMP signaling may have therapeutic implications. *Circ. Res.* **98**, 172–175 (2006).

6. H. Goldthorpe, J.-Y. Jiang, M. Taha, Y. Deng, T. Sinclair, C. X. Ge, P. Jurasz, K. Turksen, S. H. Mei, D. J. Stewart, Occlusive lung arterial lesions in endothelial-targeted, fas-induced apoptosis transgenic mice. *Am. J. Respir. Cell Mol. Biol.* **53**, 712–718 (2015).

7. S. S. Pullamsetti, R. Savai, W. Seeger, E. A. Goncharova, Translational advances in the field of pulmonary hypertension. From cancer biology to new pulmonary arterial hypertension therapeutics. Targeting cell growth and proliferation signaling hubs. *Am. J. Respir. Crit. Care Med.* **195**, 425–437 (2017).

8. S. S. Pullamsetti, B. Kojonazarov, S. Storn, H. Gall, Y. Salazar, J. Wolf, A. Weigert, N. El-Nikhely, H. A. Ghofrani, G. A. Krombach, L. Fink, S. Gattenlohner, U. R. Rapp, R. T. Schermuly, F. Grimminger, W. Seeger, R. Savai, Lung cancer-associated pulmonary hypertension: Role of microenvironmental inflammation based on tumor cell-immune cell cross-talk. *Sci. Transl. Med.* **9**, eaai9048 (2017).

9. K. W. Prins, T. Thenappan, E. K. Weir, R. Kalra, M. Pritzker, S. L. Archer, Repurposing medications for treatment of pulmonary arterial hypertension: What's old is new again. *J. Am. Heart Assoc.* **8**, e011343 (2019).

10. A. C. Dudley, Tumor endothelial cells. *Cold Spring Harb. Perspect. Med.* **2**, a006536 (2012).

11. S. Jung, S. Kim, EDDY: A novel statistical gene set test method to detect differential genetic dependencies. *Nucleic Acids Res.* **42**, e60 (2014).

12. R. S. Stearman, Q. M. Bui, G. Speyer, A. Handen, A. R. Cornelius, B. B. Graham, S. Kim, E. A. Mickler, R. M. Tuder, S. Y. Chan, M. W. Geraci, Systems analysis of the human pulmonary arterial hypertension lung transcriptome. *Am. J. Respir. Cell Mol. Biol.*, **60**, 637–649 (2019).

13. J. Barretina, G. Caponigro, N. Stransky, K. Venkatesan, A. A. Margolin, S. Kim, C. J. Wilson, J. Lehar, G. V. Kryukov, D. Sonkin, A. Reddy, M. Liu, L. Murray, M. F. Berger, J. E. Monahan, P. Morais, J. Meltzer, A. Korejwa, J. Jane-Valbuena, F. A. Mapa, J. Thibault, E. Bric-Furlong, P. Raman, A. Shipway, I. H. Engels, J. Cheng, G. K. Yu, J. Yu, P. Aspesi Jr., M. de Silva, K. Jagtap, M. D. Jones, L. Wang, C. Hatton, E. Palescandolo, S. Gupta, S. Mahan, C. Sougnez, R. C. Onofrio, T. Liefeld, L. MacConaill, W. Winckler, M. Reich, N. Li, J. P. Mesirov, S. B. Gabriel, G. Getz, K. Ardlie, V. Chan, V. E. Myer, B. L. Weber, J. Porter, M. Warmuth, P. Finan, J. L. Harris, M. Meye rson, T. R. Golub, M. P. Morrissey, W. R. Sellers, R. Schlegel, L. A. Garraway, The Cancer Cell Line Encyclopedia enables predictive modelling of anticancer drug sensitivity. *Nature* **483**, 603–607 (2012).

14. B. Seashore-Ludlow, M. G. Rees, J. H. Cheah, M. Cokol, E. V. Price, M. E. Coletti, V. Jones, N. E. Bodycombe, C. K. Soule, J. Gould, B. Alexander, A. Li, P. Montgomery, M. J. Wawer, N. Kuru, J. D. Kotz, C.-S. Hon, B. Munoz, T. Liefeld, V. Dancik, J. A. Bittker, M. Palmer, J. E. Bradner, A. F. Shamji, P. A. Clemons, S. L. Schreiber, Harnessing connectivity in a large-scale small-molecule sensitivity dataset. *Cancer Discov.* **5**, 1210–1223 (2015).

15. M. G. Rees, B. Seashore-Ludlow, J. H. Cheah, D. J. Adams, E. V. Price, S. Gill, S. Javaid, M. E. Coletti, V. L. Jones, N. E. Bodycombe, C. K. Soule, B. Alexander, A. Li, P. Montgomery, J. D. Kotz, C. S. Hon, B. Munoz, T. Liefeld, V. Dančík, D. A. Haber, C. B. Clish, J. A. Bittker, M. Palmer, B. K.

Wagner, P. A. Clemons, A. F. Shamji, S. L. Schreiber, Correlating chemical sensitivity and basal gene expression reveals mechanism of action. *Nat. Chem. Biol.* **12**, 109–116 (2016).

16. G. Speyer, D. Mahendra, H. J. Tran, J. Kiefer, S. L. Schreiber, P. A. Clemons, H. Dhruv, M. Berens, S. Kim, Differential pathway dependency discovery associated with drug response across cancer cell lines. *Pac. Symp. Biocomput.* **22**, 497–508 (2017).

17. J. Meloche, F. Potus, M. Vaillancourt, A. Bourgeois, I. Johnson, L. Deschamps, S. Chabot, G. Ruffenach, S. Henry, S. Breuils-Bonnet, E. Tremblay, V. Nadeau, C. Lambert, R. Paradis, S. Provencher, S. Bonnet, Bromodomain-containing protein 4: The epigenetic origin of pulmonary arterial hypertension. *Circ. Res.* **117**, 525–535 (2015).

18. C. Chabert, S. Khochbin, S. Rousseaux, S. Veyrenc, R. Furze, N. Smithers, R. K. Prinjha, U. Schlattner, C. Pison, H. Dubouchaud, Inhibition of BET proteins reduces right ventricle hypertrophy and pulmonary hypertension resulting from combined hypoxia and pulmonary inflammation. *Int. J. Mol. Sci.* **19**, 2224 (2018).

19. D. E. Van der Feen, K. Kurakula, E. Tremblay, O. Boucherat, G. P. L. Bossers, R. Szulcek, A. Bourgeois, M. C. Lampron, K. Habbout, S. Martineau, R. Paulin, E. Kulikowski, R. Jahagirdar, I. Schalij, H. J. Bogaard, B. Bartelds, S. Provencher, R. M. F. Berger, S. Bonnet, M. J. Goumans, Multicenter preclinical validation of BET inhibition for the treatment of pulmonary arterial hypertension. *Am. J. Respir. Crit. Care Med.* **200**, 910–920 (2019).

20. V. Cattaneo, M. V. Tribulatti, J. Carabelli, A. Carestia, M. Schattner, O. Campetella, Galectin-8 elicits pro-inflammatory activities in the endothelium. *Glycobiology* **24**, 966–973 (2014).

21. Y. R. Hadari, R. Arbel-Goren, Y. Levy, A. Amsterdam, R. Alon, R. Zakut, Y. Zick, Galectin-8 binding to integrins inhibits cell adhesion and induces apoptosis. *J. Cell Sci.* **113** (Pt. 13), 2385–2397 (2000).

22. D. J. Adams, M. Dai, G. Pellegrino, B. K. Wagner, A. M. Stern, A. F. Shamji, S. L. Schreiber, Synthesis, cellular evaluation, and mechanism of action of piperlongumine analogs. *Proc. Natl. Acad. Sci. U.S.A.* **109**, 15115–15120 (2012).

23. K. White, Y. Lu, S. Annis, A. E. Hale, B. N. Chau, J. E. Dahlman, C. Hemann, A. R. Opotowsky, S. O. Vargas, I. Rosas, M. A. Perrella, J. C. Osorio, K. J. Haley, B. B. Graham, R. Kumar, R. Saggar, R. Saggar, W. D. Wallace, D. J. Ross, O. F. Khan, A. Bader, B. R. Gochuico, M. Matar, K. Polach, N. M. Johannessen, H. M. Prosser, D. G. Anderson, R. Langer, J. L. Zweier, L. A. Bindoff, D. Systrom, A. B. Waxman, R. C. Jin, S. Y. Chan, Genetic and hypoxic alterations of the microRNA-210-ISCU1/2 axis promote iron-sulfur deficiency and pulmonary hypertension. *EMBO Mol. Med.* **7**, 695–713 (2015).

24. W. Harshbarger, S. Gondi, S. B. Ficarro, J. Hunter, D. Udayakumar, D. Gurbani, W. D. Singer, Y. Liu, L. Li, J. A. Marto, K. D. Westover, Structural and biochemical analyses reveal the mechanism of glutathione S-transferase Pi 1 inhibition by the anti-cancer compound piperlongumine. *J. Biol. Chem.* **292**, 112–120 (2017).

25. T. Bertero, K. Cottrill, A. Krauszman, Y. Lu, S. Annis, A. Hale, B. Bhat, A. B. Waxman, B. N. Chau, W. M. Kuebler, S. Y. Chan, The microRNA-130/301 family controls vasoconstriction in pulmonary hypertension. *J. Biol. Chem.* **290**, 2069–2085 (2015).

26. J. J. Ryan, S. L. Archer, Emerging concepts in the molecular basis of pulmonary arterial hypertension: Part I: Metabolic plasticity and mitochondrial dynamics in the pulmonary circulation and right ventricle in pulmonary arterial hypertension. *Circulation* **131**, 1691–1702 (2015).

27. A. Yamamura, E. Fujitomi, N. Ohara, K. Tsukamoto, M. Sato, H. Yamamura, Tadalafil induces antiproliferation, apoptosis, and phosphodiesterase type 5 downregulation in idiopathic pulmonary arterial hypertension in vitro. *Eur. J. Pharmacol.* **810**, 44–50 (2017).

28. S. Uppal, A. Gegonne, Q. Chen, P. S. Thompson, D. Cheng, J. Mu, D. Meerzaman, H. S. Misra, D. S. Singer, The Bromodomain protein 4 contributes to the regulation of alternative splicing. *Cell Rep.* **29**, 2450–2460.e5 (2019).

29. X. Tang, R. Peng, Y. Ren, S. Apparsundaram, J. Deguzman, C. M. Bauer, A. F. Hoffman, S. Hamilton, Z. Liang, H. Zeng, M. E. Fuentes, J. A. Demartino, C. Kitson, C. S. Stevenson, D. C. Budd, BET bromodomain proteins mediate downstream signaling events following growth factor stimulation in human lung fibroblasts and are involved in bleomycin-induced pulmonary fibrosis. *Mol. Pharmacol.* **83**, 283–293 (2013).

30. Y. M. Khan, P. Kirkham, P. J. Barnes, I. M. Adcock, Brd4 is essential for IL-1β-Induced inflammation in human airway epithelial cells. *PLOS ONE* **9**, e95051 (2014).

31. M. Rabinovitch, C. Guignabert, M. Humbert, M. R. Nicolls, Inflammation and immunity in the pathogenesis of pulmonary arterial hypertension. *Circ. Res.* **115**, 165–175 (2014).

32. O. Mirguet, R. Gosmini, J. Toum, C. A. Clement, M. Barnathan, J. M. Brusq, J. E. Mordaunt, R. M. Grimes, M. Crowe, O. Pineau, M. Ajakane, A. Daugan, P. Jeffrey, L. Cutler, A. C. Haynes, N. N. Smithers, C. W. Chung, P. Bamborough, I. J. Uings, A. Lewis, J. Witherington, N. Parr, R. K. Prinjha, E. Nicodeme, Discovery of epigenetic regulator I-BET762: Lead optimization to afford a clinical candidate inhibitor of the BET bromodomains. *J. Med. Chem.* **56**, 7501–7515 (2013).

33. Q. Yu, S. Y. Chan, Mitochondrial and metabolic drivers of pulmonary vascular endothelial dysfunction in pulmonary hypertension. *Adv. Exp. Med. Biol.* **967**, 373–383 (2017).

34. Y. Wang, X. Yu, H. Song, D. Feng, Y. Jiang, S. Wu, J. Geng, The STAT-ROS cycle extends IFN-induced cancer cell apoptosis. *Int. J. Oncol.* **52**, 305–313 (2018).

35. H. K. Eltzschig, P. Carmeliet, Hypoxia and inflammation. *N. Engl. J. Med.* **364**, 656–665 (2011).

36. J. D. Brown, C. Y. Lin, Q. Duan, G. Griffin, A. Federation, R. M. Paranal, S. Bair, G. Newton, A. Lichtman, A. Kung, T. Yang, H. Wang, F. W. Luscinskas, K. Croce, J. E. Bradner, J. Plutzky, NF-κB directs dynamic super enhancer formation in inflammation and atherogenesis. *Mol. Cell* **56**, 219–231 (2014).

37. S. Y. Chan, Y. Y. Zhang, C. Hemann, C. E. Mahoney, J. L. Zweier, J. Loscalzo, MicroRNA-210 controls mitochondrial metabolism during hypoxia by repressing the iron-sulfur cluster assembly proteins ISCU1/2. *Cell Metab.* **10**, 273–284 (2009).

38. J. Zhao, J. Florentin, Y.-Y. Tai, S. Torrino, L. Ohayon, T. Brzoska, Y. Tang, J. Yang, V. Negi, C. C. Woodcock, M. G. Risbano, S. M. Nouraie, P. Sundd, T. Bertero, P. Dutta, S. Y. Chan, Long range endocrine delivery of circulating miR-210 to endothelium promotes pulmonary hypertension. *Circ. Res.* **127**, 677–692 (2020).

39. K. D. Tew, D. M. Townsend, Regulatory functions of glutathione S-transferase P1-1 unrelated to detoxification. *Drug Metab. Rev.* **43**, 179–193 (2011).

40. Y. J. Chen, C. T. Lu, K. Y. Huang, H. Y. Wu, Y. J. Chen, T. Y. Lee, GSHSite: Exploiting an iteratively statistical method to identify s-glutathionylation sites with substrate specificity. *PLOS ONE* **10**, e0118752 (2015).

41. F. Virga, F. Cappellesso, B. Stijlemans, A. T. Henze, R. Trotta, J. Van Audenaerde, A. S. Mirchandani, M. A. Sanchez-Garcia, J. Vandewalle, F. Orso, C. Riera-Domingo, A. Griffa, C. Ivan, E. Smits, D. Laoui, F. Martelli, L. Langouche, G. Van den Berghe, O. Feron, B. Ghesquiere, H. Prenen, C. Libert, S. R. Walmsley, C. Corbet, J. A. Van Ginderachter, M. Ivan, D. Taverna, M. Mazzone, Macrophage miR-210 induction and metabolic reprogramming in response to pathogen interaction boost life-threatening inflammation. *Sci. Adv.* **7**, eabf0466 (2021).

42. P. Polamreddy, N. Gattu, The drug repurposing landscape from 2012 to 2017: Evolution, challenges, and possible solutions. *Drug Discov. Today*, **24**, 789–795 (2019).

43. D. E. Gordon, G. M. Jang, M. Bouhaddou, J. Xu, K. Obernier, K. M. White, M. J. O'Meara, V. V. Rezelj, J. Z. Guo, D. L. Swaney, T. A. Tummino, R. Huttenhain, R. M. Kaake, A. L. Richards, B. Tutuncuoglu, H. Foussard, J. Batra, K. Haas, M. Modak, M. Kim, P. Haas, B. J. Polacco, H. Braberg, J. M. Fabius, M. Eckhardt, M. Soucheray, M. J. Bennett, M. Cakir, M. J. McGregor, Q. Li, B. Meyer, F. Roesch, T. Vallet, A. Mac Kain, L. Miorin, E. Moreno, Z. Z. C. Naing, Y. Zhou, S. Peng, Y. Shi, Z. Zhang, W. Shen, I. T. Kirby, J. E. Melnyk, J. S. Chorba, K. Lou, S. A. Dai, I. Barrio-Hernandez, D. Memon, C. Hernandez-Armenta, J. Lyu, C. J. P. Mathy, T. Perica, K. B. Pilla, S. J. Ganesan, D. J. Saltzberg, R. Rakesh, X. Liu, S. B. Rosenthal, L. Calviello, S. Venkataramanan, J. Liboy-Lugo, Y. Lin, X. P. Huang, Y. Liu, S. A. Wankowicz, M. Bohn, M. Safari, F. S. Ugur, C. Koh, N. S. Savar, Q. D. Tran, D. Shengjuler, S. J. Fletcher, M. C. O'Neal, Y. Cai, J. C. J. Chang, D. J. Broadhurst, S. Klippsten, P. P. Sharp, N. A. Wenzell, D. Kuzuoglu-Ozturk, H.-Y. Wang, R. Trenker, J. M. Young, D. A. Cavero, J. Hiatt, T. L. Roth, U. Rathore, A. Subramanian, J. Noack, M. Hubert, R. M. Stroud, A. D. Frankel, O. S. Rosenberg, K. A. Verba, D. A. Agard, M. Ott, M. Emerman, N. Jura, M. von Zastrow, E. Verdin, A. Ashworth, O. Schwartz, C. d'Enfert, S. Mukherjee, M. Jacobson, H. S. Malik, D. G. Fujimori, T. Ideker, C. S. Craik, S. N. Floor, J. S. Fraser, J. D. Gross, A. Sali, B. L. Roth, D. Ruggero, J. Taunton, T.

Kortemme, P. Beltrao, M. Vignuzzi, A. García-Sastre, K. M. Shokat, B. K. Shoichet, N. J. Krogan, A SARS-CoV-2 protein interaction map reveals targets for drug repurposing. *Nature* **583**, 459–468 (2020).

44. R. Gramatica, T. Di Matteo, S. Giorgetti, M. Barbiani, D. Bevec, T. Aste, Graph theory enables drug repurposing–how a mathematical model can drive the discovery of hidden mechanisms of action. *PLOS ONE* **9**, e84912 (2014).

45. K. Shameer, G. Dow, B. S. Glicksberg, K. W. Johnson, Y. Ze, M. S. Tomlinson, B. Readhead, J. T. Dudley, I. J. Kullo, A network-biology informed computational drug repositioning strategy to target disease risk trajectories and comorbidities of peripheral artery disease. *AMIA Jt Summits Transl. Sci. Proc.* **2017**, 108–117 (2018).

46. F. Cheng, R. J. Desai, D. E. Handy, R. Wang, S. Schneeweiss, A. L. Barabási, J. Loscalzo, Network-based approach to prediction and population-based validation of in silico drug repurposing. *Nat. Commun.* **9**, 2691 (2018).

47. Y. Hara, Y. Sassi, C. Guibert, N. Gambaryan, P. Dorfmuller, S. Eddahibi, A. M. Lompre, M. Humbert, J. S. Hulot, Inhibition of MRP4 prevents and reverses pulmonary hypertension in mice. *J. Clin. Invest.* **121**, 2888–2897 (2011).

48. S. A. Barman, F. Chen, X. Li, S. Haigh, D. W. Stepp, D. Kondrikov, K. Mahboubi, Z. Bordan, P. Traber, Y. Su, D. J. R. Fulton, Galectin-3 promotes vascular remodeling and contributes to pulmonary hypertension. *Am. J. Respir. Crit. Care Med.* **197**, 1488–1492 (2018).

49. S. Crnkovic, B. Egemnazarov, R. Damico, L. M. Marsh, B. M. Nagy, P. Douschan, K. Atsina, T. M. Kolb, S. C. Mathai, J. E. Hooper, B. Ghanim, W. Klepetko, F. Fruhwald, D. Lassner, A. Olschewski, H. Olschewski, P. M. Hassoun, G. Kwapiszewska, Disconnect between fibrotic response and right ventricular dysfunction. *Am. J. Respir. Crit. Care Med.*, **199**, 1550–1560 (2019).

50. E. A. Goncharova, mTOR and vascular remodeling in lung diseases: Current challenges and therapeutic prospects. *FASEB J.* **27**, 1796–1807 (2013).

51. B. Wang, M. Zhang, T. Takayama, X. Shi, D. A. Roenneburg, K. C. Kent, L.-W. Guo, BET bromodomain blockade mitigates intimal hyperplasia in rat carotid arteries. *EBioMedicine* **2**, 1650–1661 (2015).

52. J. A. Mazurek, B. D. Horne, W. Saeed, M. R. Sardar, R. Zolty, Galectin-3 levels are elevated and predictive of mortality in pulmonary hypertension. *Heart Lung Circ.* **26**, 1208–1215 (2017).

53. L. Weise-Cross, T. C. Resta, N. L. Jernigan, Redox regulation of ion channels and receptors in pulmonary hypertension. *Antioxid. Redox Signal.* **31**, 898–915 (2019).

54. O. Y. Mian, M. H. Khattab, M. Hedayati, J. Coulter, B. Abubaker-Sharif, J. M. Schwaninger, R. K. Veeraswamy, J. D. Brooks, L. Hopkins, D. B. Shinohara, B. Cornblatt, W. G. Nelson, S. Yegnasubramanian, T. L. DeWeese, GSTP1 Loss results in accumulation of oxidative DNA base damage and promotes prostate cancer cell survival following exposure to protracted oxidative stress. *Prostate* **76**, 199–206 (2016).

55. Y. He, L. Liu, P. Xu, N. He, D. Yuan, L. Kang, T. Jin, Association between single nucleotide polymorphisms in ADRB2, GNB3 and GSTP1 genes and high-altitude pulmonary edema (HAPE) in the Chinese Han population. *Oncotarget* **8**, 18206–18212 (2017).

56. A. Mishra, Z. Ali, A. Vibhuti, R. Kumar, P. Alam, R. Ram, T. Thinlas, G. Mohammad, M. A. Pasha, CYBA and GSTP1 variants associate with oxidative stress under hypobaric hypoxia as observed in high-altitude pulmonary oedema. *Clin. Sci.* **122**, 299–309 (2012).

57. L. Zhong, Y. P. Zhang, W. P. Fu, L. M. Dai, C. Sun, Y. Q. Wang, The relationship between GSTP1 I105V polymorphism and COPD: A reappraisal. *Am. J. Respir. Crit. Care Med.* **181**, 763–765 (2010).

58. E. Sofianopoulou, S. Kaptoge, S. Graf, C. Hadinnapola, C. M. Treacy, C. Church, G. Coghlan, J. S. R. Gibbs, M. Haimel, L. S. Howard, M. Johnson, D. G. Kiely, A. Lawrie, J. Lordan, R. V. MacKenzie Ross, J. M. Martin, S. Moledina, M. Newnham, A. J. Peacock, L. C. Price, C. J. Rhodes, J. Suntharalingam, E. M. Swietlik, M. R. Toshner, J. Wharton, M. R. Wilkins, S. J. Wort, J. Pepke-Zaba, R. Condliffe, P. A. Corris, E. Di Angelantonio, S. Provencher, N. W. Morrell, Traffic exposures, air

pollution and outcomes in pulmonary arterial hypertension: A UK cohort study analysis. *Eur. Respir. J.* **53**, 1801429 (2019).

59. K. Piska, A. Gunia-Krzyzak, P. Koczurkiewicz, K. Wojcik-Pszczola, E. Pekala, Piperlongumine (piplartine) as a lead compound for anticancer agents– Synthesis and properties of analogues: A mini-review. *Eur. J. Med. Chem.* **156**, 13–20 (2018).

60. D. P. Bezerra, F. O. de Castro, A. P. Alves, C. Pessoa, M. O. de Moraes, E. R. Silveira, M. A. Lima, F. J. Elmiro, N. M. de Alencar, R. O. Mesquita, M. W. Lima, L. V. Costa-Lotufo, In vitro and in vivo antitumor effect of 5-FU combined with piplartine and piperine. *J. Appl. Toxicol.* **28**, 156–163 (2008).

61. A. P. Acharya, Y. Tang, T. Bertero, Y. Y. Tai, L. D. Harvey, C. C. Woodcock, W. Sun, R. Pineda, N. Mitash, M. Konigshoff, S. R. Little, S. Y. Chan, Simultaneous pharmacologic inhibition of yes-associated protein 1 and glutaminase 1 via inhaled poly(Lactic-co-Glycolic) acid-encapsulated microparticles improves pulmonary hypertension. *J. Am. Heart Assoc.* **10**, e019091 (2021).

62. T. Bertero, Y. Lu, S. Annis, A. Hale, B. Bhat, R. Saggar, R. Saggar, W. D. Wallace, D. J. Ross, S. O. Vargas, B. B. Graham, R. Kumar, S. M. Black, S. Fratz, J. R. Fineman, J. D. West, K. J. Haley, A. B. Waxman, B. N. Chau, K. A. Cottrill, S. Y. Chan, Systems-level regulation of microRNA networks by miR-130/301 promotes pulmonary hypertension. *J. Clin. Invest.* **124**, 3514–3528 (2014).

63. A. Wyce, Y. Degenhardt, Y. Bai, B. Le, S. Korenchuk, M. C. Crouthame, C. F. McHugh, R. Vessella, C. L. Creasy, P. J. Tummino, O. Barbash, Inhibition of BET bromodomain proteins as a therapeutic approach in prostate cancer. *Oncotarget* **4**, 2419–2429 (2013).

64. A. Chaidos, V. Caputo, K. Gouvedenou, B. Liu, I. Marigo, M. S. Chaudhry, A. Rotolo, D. F. Tough, N. N. Smithers, A. K. Bassil, T. D. Chapman, N. R. Harker, O. Barbash, P. Tummino, N. Al-Mahdi, A. C. Haynes, L. Cutler, B. Le, A. Rahemtulla, I. Roberts, M. Kleijnen, J. J. Witherington, N. J. Parr, R. K. Prinjha, A. Karadimitris, Potent antimyeloma activity of the novel bromodomain inhibitors I-BET151 and I-BET762. *Blood* **123**, 697–705 (2014).

65. W. S. Chen, Z. Cao, S. Sugaya, M. J. Lopez, V. G. Sendra, N. Laver, H. Leffler, U. J. Nilsson, J. Fu, J. Song, L. Xia, P. Hamrah, N. Panjwani, Pathological lymphangiogenesis is modulated by galectin-8-

dependent crosstalk between podoplanin and integrin-associated VEGFR-3. *Nat. Commun.* **7**, 11302 (2016).

66. T. Bertero, W. M. Oldham, K. A. Cottrill, S. Pisano, R. R. Vanderpool, Q. Yu, J. Zhao, Y. Tai, Y. Tang, Y.-Y. Zhang, S. Rehman, M. Sugahara, Z. Qi, J. Gorcsan III, S. O. Vargas, R. Saggar, R. Saggar, W. D. Wallace, D. J. Ross, K. J. Haley, A. B. Waxman, V. N. Parikh, T. De Marco, P. Y. Hsue, A. Morris, M. A. Simon, K. A. Norris, C. Gaggioli, J. Loscalzo, J. Fessel, S. Y. Chan, Vascular stiffness mechanoactivates YAP/TAZ-dependent glutaminolysis to drive pulmonary hypertension. *J. Clin. Invest.* **126**, 3313–3335 (2016).

67. Y. Song, L. Coleman, J. Shi, H. Beppu, K. Sato, K. Walsh, J. Loscalzo, Y. Y. Zhang, Inflammation, endothelial injury, and persistent pulmonary hypertension in heterozygous BMPR2-mutant mice. *Am. J. Physiol. Heart Circ. Physiol.* **295**, H677–690 (2008).

68. M. K. Steiner, O. L. Syrkina, N. Kolliputi, E. J. Mark, C. A. Hales, A. B. Waxman, Interleukin-6 overexpression induces pulmonary hypertension. *Circ. Res.* **104**, 236–244 (2009).

69. L. Salwinski, C. S. Miller, A. J. Smith, F. K. Pettit, J. U. Bowie, D. Eisenberg, The database of interacting proteins: 2004 Update. *Nucleic Acids Res.* **32**, D449–D451 (2004).

70. A. Chatr-Aryamontri, B. J. Breitkreutz, R. Oughtred, L. Boucher, S. Heinicke, D. Chen, C. Stark, A. Breitkreutz, N. Kolas, L. O'Donnell, T. Reguly, J. Nixon, L. Ramage, A. Winter, A. Sellam, C. Chang, J. Hirschman, C. Theesfeld, J. Rust, M. S. Livstone, K. Dolinski, M. Tyers, The BioGRID interaction database: 2015 Update. *Nucleic Acids Res.* **43**, D470–478 (2015).

71. A. Ruepp, B. Waegele, M. Lechner, B. Brauner, I. Dunger-Kaltenbach, G. Fobo, G. Frishman, C. Montrone, H. W. Mewes, CORUM: The comprehensive resource of mammalian protein complexes–2009. *Nucleic Acids Res.* **38**, D497–501 (2010).

72. K. Breuer, A. K. Foroushani, M. R. Laird, C. Chen, A. Sribnaia, R. Lo, G. L. Winsor, R. E. Hancock, F. S. Brinkman, D. J. Lynn, InnateDB: Systems biology of innate immunity and beyond—Recent updates and continuing curation. *Nucleic Acids Res.* **41**, D1228–D1233 (2013).

73. S. Orchard, M. Ammari, B. Aranda, L. Breuza, L. Briganti, F. Broackes-Carter, N. H. Campbell, G. Chavali, C. Chen, N. del-Toro, M. Duesbury, M. Dumousseau, E. Galeota, U. Hinz, M. Iannuccelli, S. Jagannathan, R. Jimenez, J. Khadake, A. Lagreid, L. Licata, R. C. Lovering, B. Meldal, A. N. Melidoni, M. Milagros, D. Peluso, L. Perfetto, P. Porras, A. Raghunath, S. Ricard-Blum, B. Roechert, A. Stutz, M. Tognolli, K. van Roey, G. Cesareni, H. Hermjakob, The MIntAct project—IntAct as a common curation platform for 11 molecular interaction databases. *Nucleic Acids Res.* **42**, D358–D363 (2014).

74. L. Licata, L. Briganti, D. Peluso, L. Perfetto, M. Iannuccelli, E. Galeota, F. Sacco, A. Palma, A. P. Nardozza, E. Santonico, L. Castagnoli, G. Cesareni, MINT, the molecular interaction database: 2012 Update. *Nucleic Acids Res.* **40**, D857–D861 (2012).

75. G. Launay, R. Salza, D. Multedo, N. Thierry-Mieg, S. Ricard-Blum, MatrixDB, the extracellular matrix interaction database: Updated content, a new navigator and expanded functionalities. *Nucleic Acids Res.* **43**, D321–D327 (2015).

76. L. Bohlin, D. Edler, A. Lancichinetti, M. Rosvall, Community detection and visualization of networks with the map equation framework, in *Measuring Scholarly Impact*, Y. Ding, R. Rousseau, D. Wolfram, Eds. (Springer, 2014), pp. 3–34.

77. R. Patro, G. Duggal, M. I. Love, R. A. Irizarry, C. Kingsford, Salmon provides fast and bias-aware quantification of transcript expression. *Nat. Methods* **14**, 417–419 (2017).

78. G. Speyer, J. Kiefer, H. Dhruv, M. Berens, S. Kim, Knowledge-assisted approach to identify pathways with differential dependencies. *Pac. Symp. Biocomput.* **21**, 33–44 (2016).

79. L. C. Freeman, A set of measures of centrality based on betweenness. *Sociometry* **40**, 35–41 (1977).

80. A. A. Sergushichev, An algorithm for fast preranked gene set enrichment analysis using cumulative statistic calculation. *bioRxiv* **2016**, 060012 (2016).

81. A. Subramanian, P. Tamayo, V. Mootha, S. Mukherjee, B. Ebert, M. Gillette, A. Paulovich, S. Pomeroy, T. Golub, E. Lander, J. Mesirov, Gene set enrichment analysis: A knowledge-based approach for interpreting genome-wide expression profiles. *Proc. Natl. Acad. Sci. U.S.A.* **102**, 15545–15550 (2005).

82. M. Ashburner, C. A. Ball, J. A. Blake, D. Botstein, H. Butler, J. M. Cherry, A. P. Davis, K. Dolinski, S. S. Dwight, J. T. Eppig, M. A. Harris, D. P. Hill, L. Issel-Tarver, A. Kasarskis, S. Lewis, J. C. Matese, J. E. Richardson, M. Ringwald, G. M. Rubin, G. Sherlock, Gene ontology: Tool for the unification of biology. *Nat. Genet.* **25**, 25–29 (2000).

83. The Gene Ontology Consortium, Expansion of the Gene Ontology knowledgebase and resources. *Nucleic Acids Res.* **45**, D331–D338 (2017).

84. A. Liberzon, A. Subramanian, R. Pinchback, H. Thorvaldsdóttir, P. Tamayo, J. P. Mesirov, Molecular signatures database (MSigDB) 3.0. *Bioinformatics* **27**, 1739–1740 (2011).
